# Model Error, Information Barriers, State Estimation and Prediction in Complex Multiscale Systems

**DOI:** 10.3390/e20090644

**Published:** 2018-08-28

**Authors:** Andrew J. Majda, Nan Chen

**Affiliations:** 1 Department of Mathematics and Center for Atmosphere Ocean Science, Courant Institute of Mathematical Sciences, New York University, New York, NY 10012, USA; 2 Center for Prototype Climate Modeling, New York University Abu Dhabi, Saadiyat Island, Abu Dhabi 129188, UAE

**Keywords:** information-theoretic framework, information barrier, model error, model sensitivity, state estimation and prediction, multiscale slow-fast systems, physics-constrained nonlinear stochastic model, reduced-order models, intermittent extreme events

## Abstract

Complex multiscale systems are ubiquitous in many areas. This research expository article discusses the development and applications of a recent information-theoretic framework as well as novel reduced-order nonlinear modeling strategies for understanding and predicting complex multiscale systems. The topics include the basic mathematical properties and qualitative features of complex multiscale systems, statistical prediction and uncertainty quantification, state estimation or data assimilation, and coping with the inevitable model errors in approximating such complex systems. Here, the information-theoretic framework is applied to rigorously quantify the model fidelity, model sensitivity and information barriers arising from different approximation strategies. It also succeeds in assessing the skill of filtering and predicting complex dynamical systems and overcomes the shortcomings in traditional path-wise measurements such as the failure in measuring extreme events. In addition, information theory is incorporated into a systematic data-driven nonlinear stochastic modeling framework that allows effective predictions of nonlinear intermittent time series. Finally, new efficient reduced-order nonlinear modeling strategies combined with information theory for model calibration provide skillful predictions of intermittent extreme events in spatially-extended complex dynamical systems. The contents here include the general mathematical theories, effective numerical procedures, instructive qualitative models, and concrete models from climate, atmosphere and ocean science.

1 Introduction32 Information Theory and Information Barriers with Model Error and Some Instructive Stochastic Models5  2.1 An Information-Theoretic Framework of Quantifying Model Error and Model Sensitivity5  2.2 Information Barriers in Capturing Model Fidelity . . . . . . . . . . . . . . . . . . . . . .7   2.2.1 First Information Barrier: Using Gaussian Approximation in Non-Gaussian Models8   2.2.2 Second Information Barrier: Using Single Point Correlation to Approximate Full CorrelationMatrix . . . . . . . . . . . . . . . . . . . . . . . . . . . . . . . . . . . . .13  2.3 Intrinsic Information Barrier in Predicting Mean Response to the Change of Forcing . .15  2.4 Slow-Fast System and Reduced Model . . . . . . . . . . . . . . . . . . . . . . . . . . . . .17  2.5 Fitting Autocorrelation Function of Time Series by a Spectral Information Criteria . . . .19

3 Quantifying Model Error with Information Theory in State Estimation and Prediction23  3.1 Kalman Filter, State Estimation and Linear Stochastic Model Prediction . . . . . . . . . .23  3.2 Asymptotic Behavior of Prediction and Filtering in One-Dimensional Linear Stochastic
   Models withModel Error . . . . . . . . . . . . . . . . . . . . . . . . . . . . . . . . . . . . .26   3.2.1 Prediction . . . . . . . . . . . . . . . . . . . . . . . . . . . . . . . . . . . . . . . . .26   3.2.2 Filtering . . . . . . . . . . . . . . . . . . . . . . . . . . . . . . . . . . . . . . . . . .27   3.2.3 Comparison . . . . . . . . . . . . . . . . . . . . . . . . . . . . . . . . . . . . . . . .27  3.3 An Information Theoretical Framework for State Estimation and Prediction . . . . . . .28   3.3.1 Motivation Examples . . . . . . . . . . . . . . . . . . . . . . . . . . . . . . . . . . .28   3.3.2 Assessing the Skill of Estimation and Prediction Using Information Theory . . .30  3.4 State Estimation and Prediction for Complex Scalar Forced Ornstein–Uhlenbeck (OU)
   Processes . . . . . . . . . . . . . . . . . . . . . . . . . . . . . . . . . . . . . . . . . . . . . .31  3.5 State Estimation and Prediction for Multiscale Slow-Fast Systems . . . . . . . . . . . . .36   3.5.1 A 3 × 3 Linear Coupled Multiscale Slow-Fast System . . . . . . . . . . . . . . . .37   3.5.2 ShallowWater Flows . . . . . . . . . . . . . . . . . . . . . . . . . . . . . . . . . . .434 Information, Sensitivity and Linear Statistical Response—Fluctuation–Dissipation Theorem (FDT)47  4.1 Fluctuation–Dissipation Theorem (FDT) . . . . . . . . . . . . . . . . . . . . . . . . . . . .48   4.1.1 The General Framework . . . . . . . . . . . . . . . . . . . . . . . . . . . . . . . . .48   4.1.2 Approximate FDT Methods . . . . . . . . . . . . . . . . . . . . . . . . . . . . . . .49  4.2 Information Barrier for Linear Reduced Models in Capturing the Response in the Second Order Statistics . . . . . . . . . . . . . . . . . . . . . . . . . . . . . . . . . . . . . . . . . .50  4.3 Information Theory for Finding the Most Sensitive Change Directions . . . . . . . . . .545 Given Time Series, Using Information Theory for Physics-Constrained Nonlinear Stochastic Model for Prediction59  5.1 A General Framework . . . . . . . . . . . . . . . . . . . . . . . . . . . . . . . . . . . . . .59  5.2 Model Calibration via Information Theory . . . . . . . . . . . . . . . . . . . . . . . . . .60  5.3 Applications: Assessing the Predictability Limits of Time Series Associated with Tropical Intraseasonal Variability . . . . . . . . . . . . . . . . . . . . . . . . . . . . . . . . . . . . .616 Reduced-Order Models (ROMs) for Complex Turbulent Dynamical Systems63  6.1 Strategies for Reduced-Order Models for Predicting the Statistical Responses and UQ .63   6.1.1 Turbulent Dynamical System with Energy-Conserving Quadratic Nonlinearity .63   6.1.2 Modeling the Effect of Nonlinear Fluxes . . . . . . . . . . . . . . . . . . . . . . . .65   6.1.3 A Reduced-Order Statistical Energy Model with Optimal Consistency and Sensitivity . . . . . . . . . . . . . . . . . . . . . . . . . . . . . . . . . . . . . . . .66   6.1.4 Calibration Strategy . . . . . . . . . . . . . . . . . . . . . . . . . . . . . . . . . . .67  6.2 Physics-Tuned Linear Regression Models for Hidden (Latent) Variables . . . . . . . . . .68  6.3 Predicting Passive Tracer Extreme Events . . . . . . . . . . . . . . . . . . . . . . . . . . .71   6.3.1 Approximating Nonlinear Advection Flow Using Physics-Tuned Linear Regression Model . . . . . . . . . . . . . . . . . . . . . . . . . . . . . . . . . . . . .73   6.3.2 Predicting Passive Tracer Extreme Events with Low-Order Stochastic Models . .767 Conclusions82A Derivations of Fisher Information from Relative Entropy83B Details of the Canonical Model for Low Frequency Atmospheric Variability84C Augmented System for Prediction and Filtering Distributions85  C.1 Augmented System for Prediction . . . . . . . . . . . . . . . . . . . . . . . . . . . . . . .85  C.2 Augmented System for Filtering . . . . . . . . . . . . . . . . . . . . . . . . . . . . . . . .86

D Possible Non-Gaussian PDFs of a Linear Model with Time-Periodic Forcing Based on the Sample Points in a Single Trajectory87

References91

## 1. Introduction

Complex multiscale turbulent dynamical systems are ubiquitous in geoscience, engineering, neural science and material science [1,2,3,4,5,6,7]. They are characterized by a large dimensional state space and a large dimension of strong instabilities which transfer energy throughout the system. Key mathematical issues are their basic mathematical structural properties and qualitative features [2,3,8,9], statistical prediction and uncertainty quantification (UQ) [10,11,12], state estimation or data assimilation [13,14,15,16,17], and coping with the inevitable model errors that arise in approximating such complex systems [10,18,19,20,21]. Understanding and predicting complex multiscale turbulent dynamical systems involve the blending of rigorous mathematical theory, qualitative and quantitative modelling, and novel numerical procedures [2,22].

One of the central difficulties in studying these complex multiscale turbulent dynamical systems is that either the dynamical equations for the truth are unknown due to the lack of physical understanding or the resolution in the models is inadequate due to the current computing power [1,13,18,23,24,25]. Therefore, understanding the model error from the imperfect dynamics as well as the coarse-grained processes becomes important. From both the theoretical and practical point of view, the following issues are of great interest.

1. How to measure the skill (i.e., the statistical accuracy) of a given imperfect model in reproducing the present states and predicting the future states in an unbiased fashion?
2. How to make the best possible estimate of model sensitivity to changes in external or internal parameters by utilizing the imperfect knowledge available of the present state? What are the most sensitive parameters for the change of the model status given uncertain knowledge of the present state?
3. How to design cheap and practical reduced models that are nevertheless able to capture both the main statistical features of nature and the correct response to external/internal perturbations?
4. How to develop a systematic data-driven nonlinear modeling and prediction framework that provides skillful forecasts and allows accurate quantifications of the forecast uncertainty?
5. How to build effective models, efficient algorithms and unbiased quantification criteria for online data assimilation (state estimation or filtering) and prediction especially in the presence of model error?

Recently, an information-theoretic framework has been developed and is applied together with other mathematical tools to address all the issues mentioned above [10,26,27,28,29,30,31,32,33,34]. This information-theoretic framework provides an unbiased way to quantify the model error and model fidelity [18,35,36,37] in complex nonlinear dynamical systems, which in turn offers a systematic procedure for model selection and parameter estimation within a given class of imperfect models [1,26,27,28]. The information-theoretic framework is also capable of estimating the model sensitivity in response to the changes in both internal and external parameters [27,28]. Practically, by incorporating the so-called fluctuation–dissipation theorem for the linear statistical response [38,39,40], the information-theoretic framework allows an extremely efficient approach to assess the model sensitivity [27,28,41]. Such a sensitivity analysis becomes particularly useful in for example detecting the climate change and preventing the occurrence of undesirable extreme events [42,43]. The combination of the model fidelity and the model sensitivity then provides important guidelines for developing reduced-order models [11,44,45] and data-driven prediction strategies using physics-constrained nonlinear stochastic models [46,47,48]. Applying the information-theoretic framework for model calibration, the reduced-order models with suitable model structures are able to capture both the key dynamical and statistical features as well as the crucial nonlinear and non-Gaussian characteristics such as intermittency and extreme/rare events as observed in nature.

Nevertheless, the choice of the reduced or simplified models plays a crucial role in approximating nature. Within an improper model family, even the best model with the most elaborate calibration will result in a large model error. This is known as the information barrier and can be quantified by the information-theoretic framework [27,49,50,51,52]. In fact, the information-theoretic framework allows a rigorous decomposition of the total model error into an intrinsic barrier and an actual model error. The latter can be eliminated or at least be minimized to a negligible level by the information-optimization criterion [27,28]. Quantifying such information barriers have both theoretic and practical importance. It indicates the futility of model calibration if the information barrier is significant. It can also be used as a guidance to expand the model family of reduced models for the improvement of imperfect models. Note that information barriers appear in both the model fidelity and model sensitivity. A model with perfect model fidelity can still have a significant information barrier in response to the internal/external perturbation and in short term predictions [27].

Another important application of the information-theoretic framework is that it provides a novel and unbiased approach to assess the online data assimilation/filtering and prediction skill in complex multiscale dynamical systems [31,53,54,55,56,57,58]. The traditional path-wise measurements such as the root-mean-square error and pattern correlation [59,60] are misleading in assessing the model error in both filtering and prediction [31,61]. In fact, these traditional measurements completely fail to quantify the ability of the imperfect models in reproducing the extreme events in nature even in the linear and Gaussian setup. In addiction, these traditional path-wise measurements take into account the information only up to the second order statistics and therefore they have no skill in quantifying the features of intermittency and non-Gaussian probability density functions (PDFs) as well as other salient characteristics in nonlinear multiscale turbulent dynamical systems. On the other hand, the information-theoretic framework combining different information measurements is able to quantify the model error in an unbiased fashion and succeeds in assessing the ability of imperfect models in reproducing both the Gaussian and non-Gaussian features in filtering and forecasting complex nonlinear dynamical systems [31,61].

In practice, due to the incomplete knowledge and the limited computing power for dealing with the complex nonlinear turbulent dynamical systems of nature, reduced-order models are often designed for the state estimation and prediction [62,63,64,65,66,67,68]. Parameterizations of unresolved variables and coarse-grained processes are typically involved in the reduced-order models [69,70,71,72], which result in large uncertainties. Therefore, the reduced-order models aim at capturing the statistical features rather than a single realization of random trajectories of the complex nonlinear turbulent dynamical systems. Among all types of the reduced-order models, linear tangential approximations and Gaussian closure models are widely used in approximating the time evolutions of the statistics of nature [71,73,74,75]. Despite their simplicity and skillful behavior in some scenarios, these crude approximations fail to capture many crucial features in nature that result from the nonlinear interactions between different variables or nonlinear feedback within different scales. Therefore, nonlinear and non-Gaussian closure becomes important in describing the turbulence [76,77,78,79]. Recently, a new framework of statistical closure models has been developed for improving the skill of the reduced-order models. The new reduced-order models take into account higher-order moments but nevertheless remain computationally efficient [1,45,50]. With the model calibration based on effective information criteria, these new reduced-order models succeed in capturing the non-Gaussian statistical characteristics including intermittency and extreme events as well as memory effect and temporal correlation. The new reduced-order models have also been used to provide accurate state estimation and prediction of many high-dimensional complex nonlinear turbulent systems [80,81,82].

This research expository article blends new viewpoints and results with a current research summary of a specific perspective. It focuses on both the development and applications of the information-theoretic framework as well as the new reduced-order nonlinear modeling strategies for dealing with model error, information barriers, state estimation and prediction in complex multiscale systems. The contents include the general mathematical framework and theory, effective numerical procedures, instructive qualitative models, and concrete models from climate, atmosphere and ocean science. The remaining of the article is organized as follows. The information-theoretic framework is developed in Section 2. In the same section, various information barriers in the presence of model error are shown via simple but instructive stochastic models. In Section 3, the information-theoretic framework is applied to assess model error in state estimation and prediction with examples coming from both complex scalar models and spatially-extended multiscale turbulent systems. The advantage of the information-theoretic framework over the traditional path-wise measurements are illustrated. Section 4 deals with sensitivity and linear statistical response using the fluctuation–dissipation theorem. An efficient and effective algorithm in finding the most sensitive change directions using information theory is also included in this section. Then, in Section 5, a novel framework of data-driven physics-constrained nonlinear stochastic models and predictions is developed and is applied to predicting the time series of an important atmospheric phenomenon with strong intermittent instabilities and extreme events. Section 6 includes the development of the new effective reduced-order models that involve higher order statistical features. These new models together with the information-optimization model calibration strategy are applied to predicting passive tracer extreme events driven by spatially-extended complex dynamical systems. The article is concluded in Section 7.

## 2. Information Theory and Information Barriers with Model Error and Some Instructive Stochastic Models

### 2.1. An Information-Theoretic Framework of Quantifying Model Error and Model Sensitivity

An information-theoretic framework has recently been developed and applied to quantify model error, model sensitivity and prediction skill [10,26,27,28,29,30,31,32,33,34]. The natural way to measure the lack of information in one probability density q(u) compared with the true probability density p(u) is through the relative entropy P(p,q) [26,32,40],

(1) $$P\left(p,q\right)=\int p\log\left(\frac{p}{q}\right),$$

which is also known as Kullback–Leibler divergence or information divergence [83,84,85]. Despite the lack of symmetry, the relative entropy has two attractive features. First, P(p,q)≥0 with equality if and only if p=q. Second, P(p,q) is invariant under general nonlinear changes of variables. These provide an attract framework for assessing model errors in many applications [23,26,33,34,86,87,88,89].

To quantify the model error and information barriers, let us denote π the probability density of the perfect model, which is actually unknown in practice. Nevertheless, the least biased estimate $\pi_{L}$ based on *L* measurements of the perfect model during the training phase is typically available. Therefore, $P(\pi ,\pi_{L})$ precisely quantifies the intrinsic error that is due to the ignorance of the information beyond the *L* measurements in the perfect model. On the other hand, denote $\pi^{M}$ the probability density associated with an imperfect model. Then, the model error in the imperfect model compared with the truth is given by the difference between $\pi^{M}$ and π, which is quantified by $P(\pi ,\pi^{M})$. Note that $P(\pi ,\pi^{M})$ quantifies the overall model error. Signal-dispersion (e.g., in Equation (6)) and other decomposition methods are often used to access different components of the model error. In addition, a general description of the model error in complex turbulent systems includes both the statistical information in terms of the PDFs and the dynamical information such as the temporal autocorrelation function. The latter will be emphasized in Section 2.3, Section 2.4 and Section 2.5.

In practice, $\pi^{M}$ is determined by no more information than the available in the prefect model. In addition, the imperfect model is typically defined on a subspace of the coarse-grained, resolved variables of the perfect model. Therefore, throughout the following analysis, we focus on characterizing the statistical departures of the imperfect model dynamics relative to the perfect model on these coarse-grained variables u.

Now, consider a class of imperfect models M. The best imperfect model for the coarse-grained variables u is the $M_{*}\in M$ so that the perfect model has the smallest additional information beyond the imperfect model distribution $\pi^{M_{*}}$, namely

(2) $$P\left(\pi ,\pi^{M_{*}}\right)=\min_{M\in M}P\left(\pi ,\pi^{M}\right).$$

The following general principle [26] facilitates the practical calculation of (2),

(3) $$\begin{array}{cc} P(\pi ,\pi_{L^{\prime }}^{M}) & =P\left(\pi ,\pi_{L}\right)+P\left(\pi_{L},\pi_{L^{\prime }}^{M}\right)\\ & =\left(S\left(\pi_{L}\right)-S\left(\pi \right)\right)+P\left(\pi_{L},\pi_{L^{\prime }}^{M}\right). \end{array}$$

In (3), we have assumed in practice that only *L* measurements are available in the perfect system and the imperfect model takes into accounts $L^{\prime }$ measurements with $L^{\prime }\leq L$. In (3),

(4) $$S\left(\pi_{L}\right)-S\left(\pi \right)=-\int \pi_{L}\log\pi_{L}+\int \pi \log\pi$$

is the entropy difference, which precisely measures an intrinsic error from the *L* measurements of the prefect system. Consequently, the optimization in (2) can be computed by replacing the unknown π by the hypothetically known $\pi_{L}$ so that the optimal model within the given class satisfies

(5) $$P\left(\pi_{L},\pi_{L^{\prime }}^{M_{*}}\right)=\min_{M\in M}P\left(\pi_{L},\pi_{L^{\prime }}^{M}\right).$$

The most practical setup for utilizing the framework of empirical information theory in many applications arises when both the measurements of the perfect system and its imperfect model involve only the mean and covariance of the resolved variables u so that $\pi_{L}\;=\pi_{G}∼N\left(\bar{u},R\right)$ and $\pi^{M}:=\pi_{G}^{M}∼N\left({\bar{u}}_{M},R_{M}\right)$ are both Gaussian. In this case, $P(\pi_{G},\pi_{G}^{M})$ has the explicit formula [2,26]

(6) $$P\left(\pi_{G},\pi_{G}^{M}\right)=\left[\frac{1}{2}{\left(\bar{u}-{\bar{u}}_{M}\right)}^{*}{\left(R_{M}\right)}^{-1}\left(\bar{u}-{\bar{u}}_{M}\right)\right]+\left[-\frac{1}{2}\log\det\left(RR_{M}^{-1}\right)+\frac{1}{2}\left(tr\left(RR_{M}^{-1}\right)-N\right)\right].$$

The first term in brackets in (6) is called ‘signal’, reflecting the model error in the mean but weighted by the inverse of the model variance, $R_{M}$, whereas the second term in brackets, called `dispersion’, involves only the model error covariance ratio, $RR_{M}^{-1}$. The signal and dispersion terms in (9) are individually invariant under any (linear) change of variables which maps Gaussian distributions to Gaussians. This property is very important for unbiased model calibration.

Next, we introduce the framework for improving model fidelity and model sensitivity [26,27]. Assume both the perfect and imperfect models are perturbed and both distributions vary smoothly with parameter δ, namely,

$$\begin{array}{cc} \pi_{L,\delta }\left(u\right) & =\pi_{L}\left(u\right)+\delta \pi_{L}\left(u\right),\;\int \delta \pi_{L}\left(u\right)du=0,\\ \pi_{\delta }^{M}\left(u\right) & =\pi^{M}\left(u\right)+\delta \pi^{M}\left(u\right),\;\int \delta \pi^{M}\left(u\right)du=0. \end{array}$$

Rigorous theorems guarantee this smooth dependence under minimal hypothesis for stochastic systems [90]. By assuming the parameter δ is small enough and doing leading order Taylor expansion of (3), we reach the following result:

(7) $$\begin{array}{cc} P(\pi_{\delta },\pi_{\delta }^{M})= & S\left(\pi_{L,\delta }\right)-S\left(\pi_{\delta }\right)+P\left(\pi_{L},\pi^{M}\right)+\int \log\left(\frac{\pi_{L}}{\pi^{M}}\right)\delta \pi_{L}-\frac{\pi_{L}}{\pi^{M}}\delta \pi^{M}\\ & +\frac{1}{2}\int \left[\pi_{L}^{-1}{\left(\delta \pi_{L}\right)}^{2}+\frac{\pi_{L}}{{\left(\pi^{M}\right)}^{2}}{\left(\delta \pi^{M}\right)}^{2}-2\frac{\delta \pi_{L}\delta \pi^{M}}{\pi^{M}}\right]+O\left(\delta^{3}\right). \end{array}$$

In the case with perfect model fidelity in terms of the *L* measurements, namely $P(\pi_{L},\pi^{M})=0$ or $\pi_{L}\left(u\right)=\pi^{M}\left(u\right)$, the expansion in (7) becomes

(8) $$P\left(\pi_{\delta },\pi_{\delta }^{M}\right)=S\left(\pi_{L,\delta }\right)-S\left(\pi_{\delta }\right)+\frac{1}{2}\int \pi_{L}^{-1}{\left(\delta \pi_{L}-\delta \pi^{M}\right)}^{2}+O\left(\delta^{3}\right),$$

where the quadratic discrepancy is measured in the Fisher information metric [91,92,93].

One important scenario in practice involves measuring only the mean and covariance for an imperfect model. We denote $\pi_{2,\delta }=\pi_{G,\delta }$ the unbiased Gaussian estimate of the perfect model. For the simplicity of statement, we further assume both the covariance of the perfect and imperfect models, $R_{\delta }$ and $R_{M,\delta }$, are diagonal such that $R_{\delta }=\left(R_{k}\right)+\left(\delta R_{k}\right)$ and $R_{M,\delta }=\left(R_{M,k}\right)+\left(\delta R_{M,k}\right)$, where |k|≤N and $\left(\delta R_{k}\right)$ and $\left(\delta R_{M,k}\right)$ are the covariance response to the external perturbation which are all scalar variances. In such a Gaussian setup, Equation (7) becomes

(9) $$\begin{array}{cc} P(\pi_{\delta },\pi_{\delta }^{M})= & S\left(\pi_{G,\delta }\right)-S\left(\pi_{\delta }\right)+P\left(\pi_{G},\pi^{M}\right)\\ & +\sum_{|k|\leq N}{\left(\delta u_{k}-\delta u_{M,k}\right)}^{*}R_{M,k}^{-1}\left(u_{k}-u_{M,k}\right)-\frac{1}{2}{\left(u_{k}-u_{M,k}\right)}^{*}\frac{\delta R_{M,k}}{R_{M,k}^{2}}\left(u_{k}-u_{M,k}\right)\\ & +\frac{1}{2}\sum_{|k|\leq N}\left[\frac{R_{k}}{R_{M,k}}-1\right]\left[\frac{\delta R_{k}}{R_{k}}-\frac{\delta R_{M,k}}{R_{M,k}}\right]+O\left(\delta^{2}\right). \end{array}$$

Under the same Gaussian assumptions and perfect model fidelity, the formula in (8) becomes

(10) $$\begin{array}{cc} P(\pi_{\delta },\pi_{\delta }^{M})= & S\left(\pi_{G,\delta }\right)-S\left(\pi_{\delta }\right)+\frac{1}{2}\sum_{|k|\leq N}{\left(\delta {\bar{u}}_{k}-\delta {\bar{u}}_{M,k}\right)}^{*}R_{k}^{-1}\left(\delta {\bar{u}}_{k}-\delta {\bar{u}}_{M,k}\right)\\ & +\frac{1}{4}\sum_{|k|\leq N}R_{k}^{-2}{\left(\delta R_{k}-\delta R_{M,k}\right)}^{2}+O\left(\delta^{3}\right), \end{array}$$

where the first summation represents the signal contribution whereas the second summation represents the dispersion contribution. The formula in (9) or (10) can be applied to quantify the information barrier in the model sensitivity using imperfect models (see, for example, Section 2.3 and Section 4).

The information theory developed here plays important roles in quantifying model error, model sensitivity and information barrier, assessing data assimilation and prediction skill as well as developing new reduced-order nonlinear modeling strategies. These topics will all be addressed with instructive examples in the following sections.

### 2.2. Information Barriers in Capturing Model Fidelity

Information barriers are defined broadly as the gap of information obtained from the imperfect model related to that from the perfect one that can never be overcome. In other words, the information barriers imply the impossibility of generating stochastic models in a given model family that capture the missing physics. For example, if the minimization of the information model error in (5) remains significant, then there always exists a portion of information in the perfect model that cannot be recovered by the reduced imperfect models. Such information barriers play important roles in both model fidelity and sensitivity. Quantifying the information barriers have both theoretic and practical importance. It indicates the futility of model calibration if the information barrier is significant. It can also be used as a guidance to expand the model family of reduced models for the improvement of imperfect models.

Below, two simple but illustrative examples will be shown for the information barriers in capturing model fidelity. The study of these information barriers to more sophisticated turbulent dynamical models can be found in [30,50]. The information barrier in the model sensitivity will be discussed in Section 2.3.

#### 2.2.1. First Information Barrier: Using Gaussian Approximation in Non-Gaussian Models

The first piece of information involves using linear Gaussian models to approximate non-Gaussian nature, which is a typical (crude) strategy in many real-world applications [30,94,95]. In addition to the intrinsic barrier in capturing the higher-order statistics (namely the non-Gaussian features), the goal here is to show that there exists an information barrier using the linear Gaussian models in capturing even the second order statistics of the truth in the presence of a time-periodic forcing.

As a simple but illustrative example, consider the following nonlinear dynamical system:

(11) $$\begin{array}{cc} \frac{du}{dt} & =-\gamma u+F\left(t\right)+\sigma_{u}{\dot{W}}_{u},\\ \frac{d\gamma }{dt} & =-d_{\gamma }\left(\gamma -\hat{\gamma }\right)+\sigma_{\gamma }{\dot{W}}_{\gamma }. \end{array}$$

This is a simplified version of the model named as “stochastic parameterized extended Kalman filter (SPEKF) model” that is widely used in nonlinear data assimilation and prediction [96,97,98,99]. Here, *u* can be regarded as a resolved variable and γ is an unresolved process which interacts with *u* in a nonlinear way. The external forcing F(t) is usually a periodic function that mimics the seasonal/annual cycle or any deterministic cycle that contributes to the system. In (11), the unresolved process γ plays the role of stochastic damping and therefore the statistics of *u* can be highly non-Gaussian with intermittent instabilities. One nice property of the model in (11) is that the time evolution of all the moments can be written down in closed analytical forms [15,97].

A natural way to approximate *u* without knowing the detailed structure of the unresolved process γ is the following mean stochastic model (MSm) [15,41],

(12) $$\frac{du^{M}}{dt}=-\hat{\gamma }u^{M}+F^{M}\left(t\right)+\sigma_{u}^{M}{\dot{W}}_{u}^{M},$$

where the mean value of the hidden process γ is used in the dynamics of the resolved variable *u*. The MSm in (12) is a linear model with additive noise and therefore it has Gaussian statistics. To understand the information barrier in using the linear and Gaussian MSm to approximate the nonlinear and non-Gaussian SPEKF-type model in (11), we study the following two dynamical regimes:

(13) $$\begin{array}{cc} \mathrm{Highly}\;\mathrm{Intermittent}\;\mathrm{Regime}:\; & \sigma_{u}=0.5,\;d_{\gamma }=1.2,\;\sigma_{\gamma }=1,\;\hat{\gamma }=1.5,\\ \mathrm{Nearly}\;\mathrm{Gaussian}\;\mathrm{Regime}:\; & \sigma_{u}=0.5,\;d_{\gamma }=1.2,\;\sigma_{\gamma }=1,\;\hat{\gamma }=5.0. \end{array}$$

**Figure 1:**
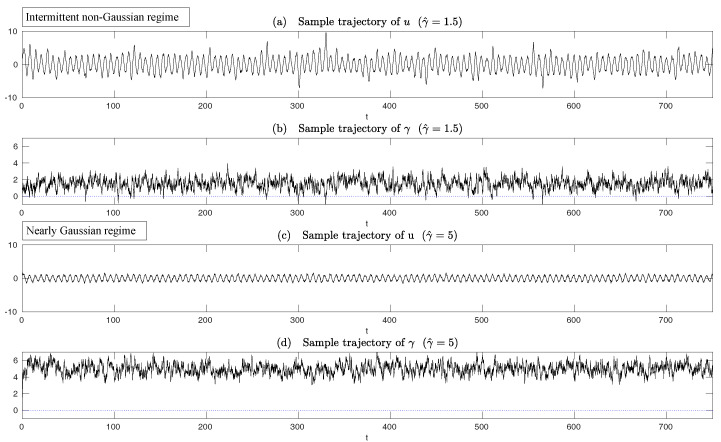
Sample trajectories of *u* and γ in the highly intermittent regime (**a**,**b**) and nearly Gaussian regime (**c**,**d**), respectively. The parameters are given in (13). In (**b**,**d**), the dotted line γ=0 indicates the instability threshold, where γ below zero corresponds to the unstable phases of *u*.

**Figure 2:**
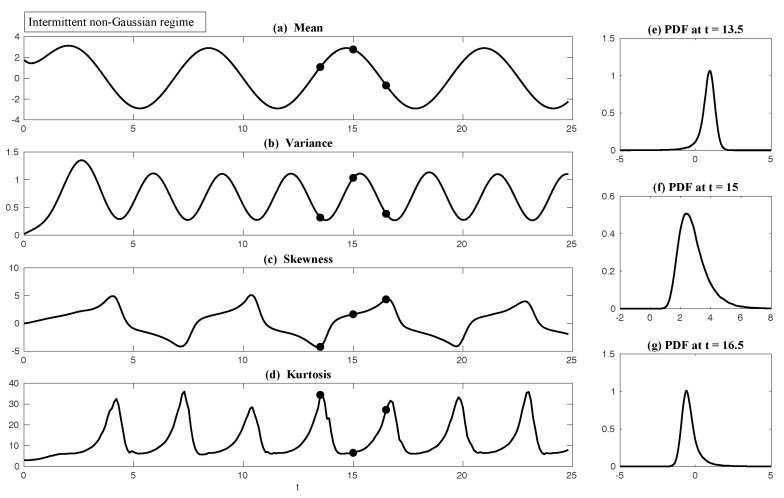
Highly intermittent regime. (**a**–**d**): the first four moments (mean, variance, skewness and kurtosis) of *u*. (**e**–**g**): Probability density functions (PDFs) of *u* at t=13.5,15 and 16.5. These simulations are based on Monte Carlo with 100,000 samples. Note that, due to the intermittent unstable events, the calculation of the high order moments of *u* becomes sensitive to the samples.

**Figure 3:**
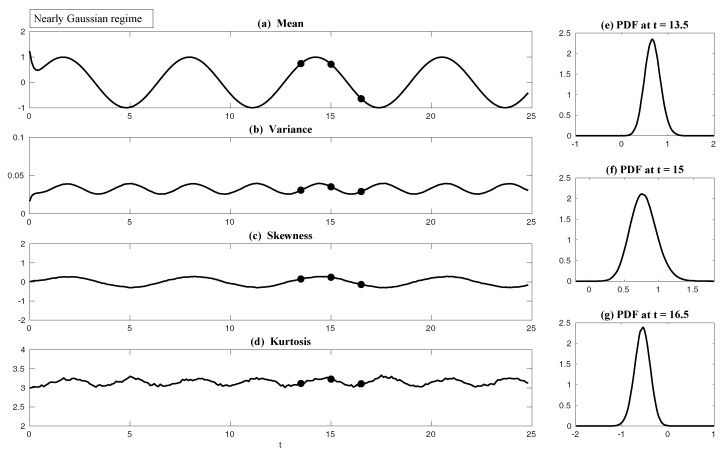
Nearly Gaussian regime. Same captions as in Figure 2.

In both regimes, the periodic forcing is given by F(t)=5sin(t). Figure 1 shows sample trajectories of *u* and γ in the two regimes, respectively. It is clear that in the highly intermittent regime, γ has a frequent transition to values below zero, which triggers large bursts in the signal of *u*, namely the intermittent instability. On the other hand, in the nearly Gaussian regime, γ stays positive and therefore the signal of *u* has no intermittent instability. In panels (a)–(d) of Figure 2, the time evolution of the first four moments of *u* is shown. Despite the strong nonlinear interactions between γ and *u*, the external forcing F(t) results in periodic behavior in all these statistics. In panels (e)–(g), the PDFs of *u* at three different time instants within one period t=13.5,15 and 16.5 are demonstrated, which are all highly non-Gaussian with significant skewness and kurtosis. As comparison, the skewness and kurtosis in the nearly Gaussian regime (Figure 3) are tiny and the amplitudes of the periodic oscillation in the variance, skewness and kurtosis become much weaker.

Let’s denote π the PDF of the nonlinear dynamics in (11) and $\pi_{G}^{M}=\pi_{2}^{M}$ that of the MSm in (12). It is clear according to (3) that there is an intrinsic barrier $S\left(\pi \right)-S\left(\pi_{G}\right)$ due to the non-Gaussian nature of π. Next, we show that there exists an information barrier in the statistics of MSm $\pi_{G}^{M}$ even compared with the Gaussian approximation of π. Below, the mean and variance of *u* from the perfect model are both computed using their closed analytical forms [97].

**Figure 4:**
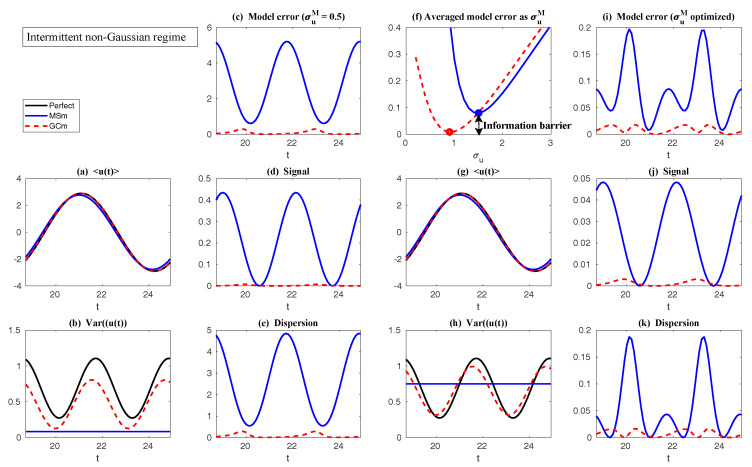
Highly intermittent regime. (**a**,**b**): time evolutions of the mean 〈u(t)〉 and variance Var(u(t)) within one period in the statistical equilibrium; (**c**–**e**): total model error, model error in the signal part and model error in the dispersion part. The results shown from both mean stochastic model (MSm) and Gaussian closure model (GCm) in (**a**–**e**) are equipped with the same parameters as those in the perfect model; (**f**): averaged model error $\bar{P(\pi ,\pi^{M})}$ as a function of $\sigma_{u}^{M}$; (**g**–**k**) are similar to those in (**a**–**e**) except that the stochastic forcing coefficient $\sigma_{u}^{M}$ is optimized.

**Figure 5:**
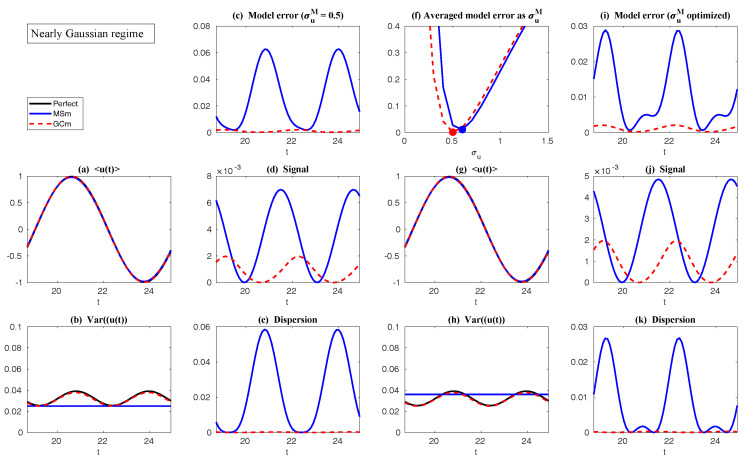
Nearly Gaussian regime. Same captions as those in Figure 4.

**Figure 6:**
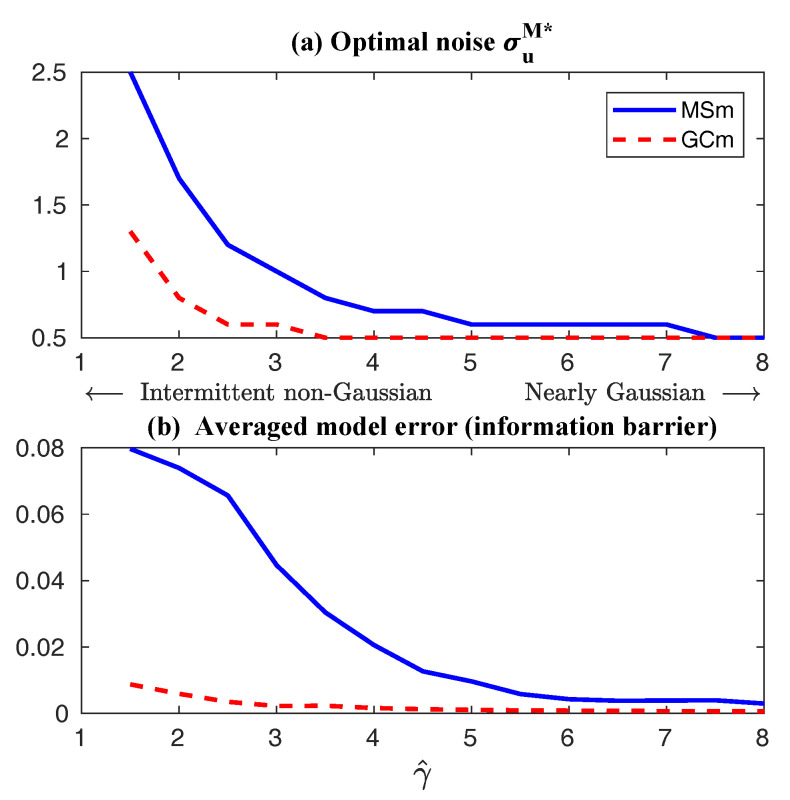
(**a**): optimal noise coefficient $\sigma_{u}^{M}$ in MSm and GCm as a function of $\hat{\gamma }$. The larger the $\hat{\gamma }$ is, the corresponding dynamical regime is more Gaussian; (**b**): the corresponding minimal information model error (information barrier) averaged over a period in the statistical equilibrium.

First, we take the same parameters $F^{M}\left(t\right)=F\left(t\right)$ and $\sigma_{u}^{M}=\sigma_{u}$ in the MSm (12) as those in the perfect model (11). The time evolutions of the mean 〈u(t)〉 and the variance Var(u(t)) within one period at the statistical equilibrium from the MSm (blue) are shown in panels (a) and (b) of Figure 4. Although the evolution of the mean using MSm is quite close to the truth (black), the variance is strongly underestimated. This is as expected since a large portion of the variance comes from the intermittent events while the MSm stabilizes the system and does not allow such large bursts. As a consequence, the dispersion part of the model error, as defined in (6), becomes huge. Interestingly, despite the small error in the time evolution of the mean (panel (a)), the signal part of the model error remains significant. In fact, according to (6), the signal part of the model error is weighted by the inverse of the model variance, the severe underestimation of which results in such a large error. To overcome the issue of underestimating the variance, a common strategy in improving imperfect model is to inflate the stochastic forcing coefficient $\sigma_{u}^{M}$ [29,100,101]. Here, the optimization is based on the minimization of the averaged information content $\bar{P(\pi ,\pi^{M})}$ between the perfect and imperfect models within one period at the statistical equilibrium. In panel (f), we show $\bar{P(\pi ,\pi^{M})}$ as a function of $\sigma_{u}^{M}$. With an inflation of $\sigma_{u}^{M}$, the model error does decrease significantly. However, even at the minimum of the curve where $\sigma_{u}^{M*}=1.5$, the model error is still far from zero. In fact, due to the linear nature of the MSm, the forcing $F^{M}\left(t\right)$ in the MSm only affects the evolution of the mean. The periodic behavior in the variance can never be captured by the MSm (see panel (h)), which leads to an information barrier. As comparison, the nonlinearity in the nearly Gaussian regime as shown in Figure 5 is much weaker and therefore the information barrier becomes insignificant. In Figure 6, the optimal stochastic forcing $\sigma_{u}^{M*}$ as well as the information barrier as a function of $\hat{\gamma }$ are shown. It is clear that the information barrier decreases when the dynamical regime goes more towards Gaussian (with an increase of $\hat{\gamma }$).

The above analysis indicates an important fact. That is, even if only the first two moments (mean and variance) are taken into account in the perfect model, the linear MSm still fails to capture the evolution of these Gaussian statistics, which evolve in a strongly nonlinear way driven by the underlying nonlinear perfect dynamics. Such an information barrier cannot be overcome unless the imperfect model contains nonlinear information. In practice, various closure models are used to approximate the nonlinear behavior in the underlying perfect model. Below, we briefly report the results using a simple Gaussian closure model (GCm) [73,102,103]. Recall the Reynolds’ decomposition

$$u=\bar{u}+u^{\prime },\;\gamma =\bar{\gamma }+\gamma^{\prime },$$

where $\bar{\cdot }$ is the ensemble mean and $\cdot^{\prime }$ is the fluctuation with $\bar{\cdot^{\prime }}=0$. With the Reynolds’ decomposition, the evolution equations of the mean $\langle u\rangle =\bar{u},\langle \gamma \rangle =\bar{\gamma }$, the variance $Var\left(u\right)=\bar{u^{\prime 2}},Var\left(\gamma \right)=\bar{\gamma^{\prime 2}}$ and the covariance $\mathrm{Cov}\left(u^{\prime },\gamma^{\prime }\right)=\bar{u^{\prime }\gamma^{\prime }}$ are given by

(14) $$\begin{array}{cc} d\bar{u} & =(-\bar{\gamma }\bar{u}-\bar{u^{\prime }\gamma^{\prime }}+F^{M}\left(t\right))dt,\\ d\bar{\gamma } & =-d_{\gamma }^{M}\left(\bar{\gamma }-{\hat{\gamma }}^{M}\right)dt,\\ d\bar{u^{\prime 2}} & =(-2\bar{u}\bar{u^{\prime }\gamma^{\prime }}-2\bar{u^{\prime 2}}\bar{\gamma }+{\left(\sigma_{u}^{M}\right)}^{2}-2\bar{u^{\prime 2}\gamma^{\prime }})dt,\\ d\bar{\gamma^{\prime 2}} & =(-2d_{\gamma }^{M}\bar{\gamma^{\prime 2}}+{\left(\sigma_{\gamma }^{M}\right)}^{2})dt,\\ d\bar{u^{\prime }\gamma^{\prime }} & =[-\left(\bar{\gamma }+d_{\gamma }^{M}\right)\bar{u^{\prime }\gamma^{\prime }}-\bar{u}\bar{u^{\prime 2}}-\bar{u^{\prime }\gamma^{\prime 2}}]dt. \end{array}$$

Note that the third and the fifth equations of (14) involve triad interactions $\bar{u^{\prime }\gamma^{\prime 2}}$ and $-2\bar{u^{\prime 2}\gamma^{\prime }}$, which represent the third order moments. These triad terms come from the nonlinearity of the underlying perfect system. In fact, the perfect model involves quadratic nonlinearity, and therefore the evolution of the *k*-th order moments always depend on the k+1-th order ones. To close the system, the Gaussian closure model assumes $\bar{u^{\prime }\gamma^{\prime 2}}=-2\bar{u^{\prime 2}\gamma^{\prime }}=0$. The resulting system then involves only the interactions between the mean and covariance,

(15) $$\begin{array}{cc} d\bar{u} & =(-\bar{\gamma }\bar{u}-\bar{u^{\prime }\gamma^{\prime }}+F^{M}\left(t\right))dt,\\ d\bar{\gamma } & =-d_{\gamma }^{M}\left(\bar{\gamma }-{\hat{\gamma }}^{M}\right)dt,\\ d\bar{u^{\prime 2}} & =(-2\bar{u}\bar{u^{\prime }\gamma^{\prime }}-2\bar{u^{\prime 2}}\bar{\gamma }+{\left(\sigma_{u}^{M}\right)}^{2})dt,\\ d\bar{\gamma^{\prime 2}} & =(-2d_{\gamma }^{M}\bar{\gamma^{\prime 2}}+{\left(\sigma_{\gamma }^{M}\right)}^{2})dt,\\ d\bar{u^{\prime }\gamma^{\prime }} & =[-\left(\bar{\gamma }+d_{\gamma }^{M}\right)\bar{u^{\prime }\gamma^{\prime }}-\bar{u}\bar{u^{\prime 2}}]dt. \end{array}$$

In Figure 4, it is clear from panel (b) that even using exactly the same parameters in GCm as in the perfect one and without optimizing $\sigma_{u}^{M}$, the variance recovered from the GCm is much improved compared with that from the MSm. This indicates the fact that the information barrier can be largely overcome by taking into account the nonlinearity in the imperfect model. The remaining error comes from ignoring the third order moments $\bar{u^{\prime }\gamma^{\prime 2}}$ and $-2\bar{u^{\prime 2}\gamma^{\prime }}$, which are nonzero in the intermittent non-Gaussian regime. More elaborate closure model techniques involve calibrating the third order moments using various approximations [11,44,104], which are not necessary in this simple example but have been shown to be crucial for more complex turbulent dynamical systems. Such topics will be discussed in detail in Section 6.3. Notably, the periodic behavior in the variance using the GCm is captured due to the nonlinear interactions between mean and variance. For example, the time-periodic mean $\bar{u}$ appears in the equation of $\bar{u^{\prime 2}}$. This is a significant difference compared with the linear MSm. Finally, with the optimal choice of $\sigma_{u}^{M}$ (panel (f)), the information model error here becomes negligible.

#### 2.2.2. Second Information Barrier: Using Single Point Correlation to Approximate Full Correlation Matrix

The strategy with single point statistics is widely used in climate science [105]. The single point statistics takes into account only the variance at each grid point and ignores the correlations between different grids. Despite the fact that both equilibrium consistency and sensitivity in response in single point statistics can be achieved by turning at most one parameter of the imperfect model, such a strategy is not enough for desirable model performance, which can be measured by the information barrier [10,26,27]. Such an information barrier was first quantified in [44,50]. In the following, we show this information barrier.

Let the PDF from the true model be π(u) with $u={\left(u_{0},u_{1},\ldots u_{J-1}\right)}^{T}$ as before. Consider a Gaussian imperfect model where we only measure pointwise marginal PDF $\pi_{1pt}^{M}\left(u_{j}\right)\equiv \pi_{1pt,j}^{M}$ at each grid pint j=0,…,J-1. Then, we construct the PDF with only single point statistics from the marginal distribution as $\pi_{1pt}^{M}=\prod_{j=0}^{J-1}\pi_{1pt,j}^{M}$. According to [34], the information distance between the truth and imperfect model prediction has the form:

(16) $$P\left(\pi ,\pi_{1pt}^{M}\right)=S\left(\pi_{G}\right)-S\left(\pi \right)+P\left(\pi_{G},\prod_{j=0}^{J-1}\pi_{1pt,j}^{G}\right)+\sum_{j=0}^{J-1}P\left(\pi_{1pt,j}^{G},\pi_{1pt,j}^{M}\right),$$

with $\pi_{1pt,j}^{G}=N\left({\bar{u}}_{j},R_{j}\right)$. The first part on the right-hand side of (16) is the intrinsic information barrier in Gaussian approximation. The third part is the model error in the imperfect model as compared to the single point statistics of the perfect model Gaussian fit, which can be vanished (or at least be minimized) by calibrating the imperfect model. The error from single point approximation by ignoring the cross-covariance then comes only from the information barrier in the marginal approximation as shown in the second part on the right-hand side of (16).

Below, we assume the true system is statistically homogeneous, which means the statistics is invariant at different grid points. This is in fact a typical feature in many real applications [2,106,107]. With statistical homogeneity, it is straightforward to show [50] that the diagonal entries of the covariance matrix corresponding to the single point approximation are all the same $R_{1pt}$. In addition, the covariance matrix $\hat{R}$ in the spectral space (associated with the Fourier modes of u) is also a diagonal matrix. Denote ${\hat{R}}_{j}$ the *j*-th diagonal entry of $\hat{R}$:

(17) $${\hat{R}}_{j}=J\sum_{n=0}^{J-1}\langle u_{0}^{\prime }u_{n}^{\prime }\rangle e^{2\pi inj/J},$$

where $u_{n}^{\prime }$ is the *n*-th component of u by subtracting the mean. Therefore, ${\hat{R}}_{1pt}=\frac{\sum_{j=0}^{J-1}{\hat{R}}_{j}}{J}$. By further assuming the same pointwise mean for $\pi_{G}$ and its single point approximation, the information barrier due to single point statistics approximation becomes

(18) $$\begin{array}{cc} P\left(\pi_{G},\prod_{j=0}^{J-1}\pi_{1pt,j}^{G}\right) & =\sum_{k=J/2+1}^{J/2}\left[-\log\det\left({\hat{R}}_{k}{\hat{R}}_{1pt}^{-1}\right)+tr\left({\hat{R}}_{k}{\hat{R}}_{1pt}^{-1}-I\right)\right]\\ & =-\sum_{k=J/2+1}^{J/2}\log\det\left({\hat{R}}_{k}{\hat{R}}_{1pt}^{-1}\right)+tr\left[\sum_{k=J/2+1}^{J/2}\left({\hat{R}}_{k}{\hat{R}}_{1pt}^{-1}-I\right)\right]\\ & =-\log\left(\prod_{k=J/2+1}^{J/2}\frac{\det{\hat{R}}_{k}}{\det{\hat{R}}_{1pt}}\right)\\ & =J\log\left[\frac{\det\left(\sum_{j=0}^{J-1}{\hat{R}}_{j}/J\right)}{{\left(\prod_{j=0}^{J-1}\det{\hat{R}}_{j}\right)}^{1/J}}\right], \end{array}$$

where the second equality just applies the definition of $R_{1pt}$ such that $\sum_{k=-J/2+1}^{J/2}\left(R_{k}R_{1pt}^{-1}-I\right)=0$.

In addition to compute the information barrier explicitly using (18), the following result provides an effective estimation of such an information barrier,

(19) $$P\left(\pi_{G},\prod_{j=0}^{J-1}\pi_{1pt,j}^{G}\right)∼O\left({\left(\sigma_{max}-\sigma_{min}\right)}^{2}\right),$$

where $\sigma_{max}$ and $\sigma_{min}$ are the largest and smallest variances in $\hat{R}$. See [50] for more details.

Below, we construct a simple linear system to illustrate the information barrier due to the single point statistics approximation and showing the calculation of the formula in (18). An illustration of such information barrier based on a more sophisticated (40-dimensional) turbulent system is included in [50]. Here, the truth is given by a two-dimensional linear model,

(20) $$\begin{array}{cc} \frac{du_{0}}{dt} & =L_{00}u_{0}+L_{01}u_{1}+\sigma_{u_{0}}{\dot{W}}_{u_{0}},\\ \frac{du_{1}}{dt} & =L_{10}u_{0}+L_{11}u_{1}+\sigma_{u_{1}}{\dot{W}}_{u_{1}}, \end{array}$$

with the following parameters

(21) $$L=\left(\begin{array}{cc} L_{00} & L_{01}\\ L_{10} & L_{11} \end{array}\right)=\left(\begin{array}{cc} -1 & 0.5\\ 0.5 & -1 \end{array}\right),\;\Sigma =\left(\begin{array}{cc} \sigma_{u_{0}} & \\ & \sigma_{u_{1}} \end{array}\right)=\left(\begin{array}{cc} 1 & \\ & 1 \end{array}\right).$$

**Figure 7:**
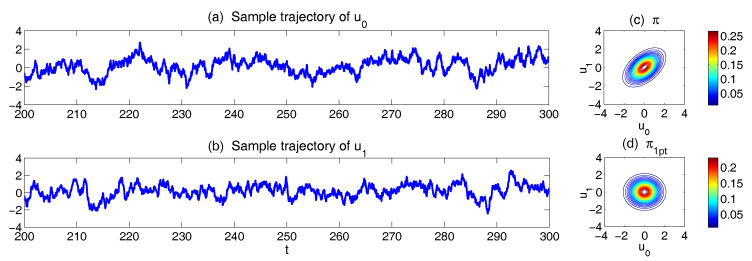
(**a**,**b**): sample trajectories of the two-dimensional model (20) with parameters (21); (**c**): true joint PDF associated with (**a**,**b**); (**d**): joint PDF with single point statistics approximation.

It is easy to check that the two eigenvalues of L are $\lambda_{1}=-1.5$ and $\lambda_{2}=-0.5$ and therefore the linear system (20) is stable. In addition, due to the non-zero coefficients $L_{01}$ and $L_{10}$, $u_{0}$ and $u_{1}$ are correlated. Figure 7 shows the sample trajectories of the two-dimensional model (20) with parameters (21). The covariance matrix and statistical equilibrium can be written down explicitly [15],

(22) $$R=\frac{1}{3}\left(\begin{array}{cc} 2 & 1\\ 1 & 2 \end{array}\right).$$

The non-zero off-diagonal entries clearly indicate the cross-covariance between $u_{0}$ and $u_{1}$. Now, we implement the single point statistics approximation, which ignores the off-diagonal entries in (22) and the result is

(23) $$R_{1pt}=\frac{1}{3}\left(\begin{array}{cc} 2 & \\ & 2 \end{array}\right).$$

Since this example is extremely simple, it is straightforward to compute the information barrier by plugging the covariance matrices (22) and (23) as well as the zero mean into the explicit formula of the relative entropy (6), which yields

(24) $$P\left(\pi_{G},\prod_{j=0}^{J-1}\pi_{1pt,j}^{G}\right)=0.1438.$$

Alternatively, according to (17), it is also easy to show that $\hat{R}$ is a diagonal matrix and is given by

(25) $$\hat{R}=\left(\begin{array}{cc} {\hat{R}}_{0} & \\ & {\hat{R}}_{1} \end{array}\right),$$

where

(26) $$\begin{array}{cc} {\hat{R}}_{0} & =2\left(\langle u_{0}u_{0}\rangle +\langle u_{0}u_{1}\rangle \right)=2,\\ {\hat{R}}_{1} & =2\left(\langle u_{0}u_{0}\rangle -\langle u_{0}u_{1}\rangle \right)=\frac{2}{3}. \end{array}$$

Plugging (26) into (18) gives the same result as in (24). This clearly shows the information barrier due to single point statistics approximation. Panels (c) and (d) in Figure 7 show true joint probability density function (PDF) $\pi (u_{0},u_{1})$ and the one with single point statistics approximation, the difference between which indicates the information barrier.

### 2.3. Intrinsic Information Barrier in Predicting Mean Response to the Change of Forcing

In Section 2.2, we have demonstrated the information barrier in the model fidelity via simple but illustrative examples. In this section, we aim at using a simple example to illustrate the intrinsic information barrier in the model sensitivity. Ref. [27] is a good reference for this topic. The example shown here also reveals the following fact. Even if the model fidelity represented by the equilibrium PDF is captured, the dynamical feature in the perfect model can still be missed if the model sensitivity is not recovered by the imperfect model. Therefore, both the model fidelity and model sensitivity are required in calibrating the imperfect model, which will be discussed with more details in Section 5 and Section 6.

Here, the focus is the mean response to the change of forcing in linear models. A general framework of any system response (e.g., variance or higher order moments) to different types of external perturbations (e.g., forcing, dissipation or phase) in complex nonlinear model will be developed in Section 4 using the so-called fluctuation–dissipation theorem (FDT).

Consider a general linear system with noise

(27) $$\frac{du}{dt}=Lu+F+\sigma \dot{W}.$$

In (27), L is a linear operator whose eigenvalues all have a negative real part, which guarantees the existence of a Gaussian statistical steady state of u. Here F is an external forcing, which can be a function of time *t*, and $\dot{W}$ is stochastic white noise. Now, we impose a forcing perturbation δF to the original system in (27)

(28) $$\frac{du^{\delta }}{dt}=Lu^{\delta }+F+\delta F+\sigma \dot{W}.$$

Since both (27) and (28) are linear models, the mean values 〈u〉 and $\langle u^{\delta }\rangle$ at the statistical steady state can be written explicitly,

$$\langle u\rangle =L^{-1}F,\;\mathrm{and}\;\langle u^{\delta }\rangle =L^{-1}\left(\delta F+F\right).$$

Therefore, the mean response of u to the forcing perturbation δF is given by

(29) $$\langle \delta u\rangle =\langle u^{\delta }-u\rangle =L^{-1}\delta F.$$

In practice, model error is usually inevitable. A suitable imperfect model is expected to generate at least the same mean response as in the perfect model in (29) in addition to the model fidelity.

A typical situation with model error for complex systems arises when the true system has additional degrees of freedom that are hidden from the family of imperfect models due to either the lack of scientific understanding or practical lack of computational resolution. The simple example below involves such features.

Consider the following perfect model with linear stochastic equations,

(30) $$\begin{array}{cc} \frac{du}{dt} & =au+v+F,\\ \frac{dv}{dt} & =qu+Av+\sigma \dot{W}, \end{array}$$

where $\dot{W}$ is white noise. The system in (30) has a smooth Gaussian statistical steady state provided that

(31) a+A<0,aA-q>0.

In (30), *u* can be treated as a resolved variable while *v* is a hidden one. All the imperfect models are given by the scalar stochastic equation that involves only the process of the observed variable,

(32) $$\frac{du_{M}}{dt}=-\gamma_{M}u_{M}+F_{M}+\sigma_{M}{\dot{W}}_{M}.$$

It is natural to require $\gamma_{M}>0$ such that the imperfect model (32) has a Gaussian statistical steady state. Next, the imperfect model (32) is tuned to capture the model fidelity in the perfect system (30) by matching the equilibrium mean and variance of *u* and $u_{M}$. This implies

(33) $$\frac{F_{M}}{\gamma_{M}}=-\frac{AF}{aA-q},\;\frac{\sigma_{M}^{2}}{2\gamma_{M}}=\frac{\sigma^{2}}{2(a+A)(aA-q)}\equiv E$$

with a suitable choice of the three tuning parameters $F_{M},\sigma_{M}$ and $\gamma_{M}\left(>0\right)$, it is clear that the conditions in (33) can always be satisfied.

In addition to the model fidelity, an important and practical issue is to understand the response of the system to the external forcing perturbation δF. Therefore, it is crucial to have an imperfect model that has the same forcing response as the perfect model. To test the response of the external forcing, we replace *F* and $F_{M}$ by F+δF and $F_{M}+\delta F$ in the two linear systems (30) and (32), respectively. Note that the external forcing will not change the variance in linear systems. The only change in the equilibrium response is through the change in mean,

(34) $$\delta u=-\frac{A}{aA-q}\delta F,\;\delta u_{M}=\frac{1}{\gamma_{M}}\delta F.$$

Now assume A>0 in the perfect model (30). We claim that no model in the family (32) can match the response of *u* correctly. In fact, with A>0 and aA-q>0 as required in (31), δu∝-δF. However, $\gamma_{M}>0$ implies $\delta u_{M}\propto \delta F$. In other words, the responses in the perfect and imperfect models are always anti-correlated, which implies an information barrier. To quantify this information barrier, we insert the response of mean (34) into (10) and make use of the fact that the response in the variance is always zero. Then, (10) yields

(35) $$P\left(\pi_{\delta },\pi_{\delta }^{M}\right)=\frac{1}{2}E^{-1}\left|-\frac{A}{aA-q}-\frac{1}{\gamma_{M}}\right|{\left|\delta F\right|}^{2}.$$

It is clear that with A>0 there is no finite minimum over $\gamma_{M}$ of (34) and necessarily $\gamma_{M}\to \infty$ in the approach to this minimum value. Thus, there is an intrinsic information barrier to skill in the mean response that cannot be overcome with the imperfect models in (32) even if they satisfy perfect model fidelity (33). On the other hand, if A<0, then (35) indicates a unique minimum with $\gamma_{M}^{*}=-A^{-1}\left(aA-q\right)$, in which case both the model fidelity and mean response are captured.

### 2.4. Slow-Fast System and Reduced Model

An important practical issue for complex dynamical systems is how to account for the indirect influence of the unresolved variables $u_{\mathrm{II}}$ on the response of the resolved variables $u_{I}$ beyond bare truncation formulae. The importance of this has already been in Section 2.2 and Section 2.3 for calibrating the model fidelity and predicting the mean response using linear imperfect models. Understanding this issue also has practical significance since simplified models are always preferred to decrease the computational cost in solving the complex multiscale dynamical systems. Therefore, developing reduced stochastic models for the variables $u_{I}$ with high skill for the low-frequency response is a central issue. While nature can be highly nonlinear and non-Gaussian, the focus here is linear systems with slow-fast multiscale features. Below, we will show that the stochastic mode reduction techniques [40,108,109,110] are able to produce a reduced stochastic model for the low-frequency variables $u_{I}$. Despite its simplicity, such a reduced stochastic model has exactly the same mean response operator as that in the complete stochastic system! More Gaussian and non-Gaussian examples can be found in [111].

Consider a linear multiscale stochastic model for variables $u={\left(u_{I},u_{\mathrm{II}}\right)}^{T}$ given by

(36) $$\begin{array}{cc} \frac{du_{I}}{dt} & =L_{11}u_{I}+L_{12}u_{\mathrm{II}}+F_{I},\\ \frac{du_{\mathrm{II}}}{dt} & =L_{21}u_{I}-\frac{\Gamma }{\epsilon }L_{22}u_{\mathrm{II}}+F_{\mathrm{II}}+\frac{\sigma }{\epsilon^{1/2}}\dot{W}, \end{array}$$

which can also be written in a compact form

(37) $$\frac{du}{dt}=L^{\epsilon }u+\sigma^{\epsilon }\dot{W}+F,\;\mathrm{with}\;L^{\epsilon }=\left(\begin{array}{cc} L_{11} & L_{12}\\ L_{21} & -\frac{\Gamma }{\epsilon } \end{array}\right).$$

The parameter ϵ>0 in (36) can be large or small. Here, we require that $L^{\epsilon }$ has eigenvalues with a negative real part for all ϵ and in particular

(38) $$\left(L_{11}u,u\right)<0,\;\left(\Gamma u,u\right)>0.$$

for u≠0. These requirements guarantee that $L^{\epsilon }$ is invertible and the climate mean state is given by $\langle u\rangle =-{\left(L^{\epsilon }\right)}^{-1}\langle F\rangle$. This together with (37) and (38) implies in particular that the change in the first components of the climate mean state, $\delta \langle u_{I}\rangle$, in response to a change in forcing, $\delta F_{1}$, is given exactly by

(39) $$\begin{array}{cc} \delta \langle u_{I}\rangle & ={\left(L^{\epsilon }\right)}_{11}^{-1}\delta F_{I},\\ {\left(L^{\epsilon }\right)}_{11}^{-1} & ={\left(L_{11}+\epsilon L_{12}\Gamma^{-1}L_{21}\right)}^{-1}. \end{array}$$

Stochastic mode reduction techniques [40,108,109,110] systematically produce a reduced stochastic model for the variables $u_{I}$ alone, which is a valid model in the limit ϵ→0; such models often have significant skill for moderate variables of ϵ [112,113]. Here, we focus on their skill in producing infinite time-mean response in (39) of the full dynamics from (36) independent of ϵ.

First, the local equations in (36) can be rewritten exactly as an equivalent equation with memory in time for the $u_{I}$ variable alone [114] given by

(40) $$\frac{du_{I}}{dt}=L_{11}u_{I}+F_{I}+L_{12}\int_{0}^{t}e^{-(\Gamma /\epsilon )(t-s)}\left[L_{12}u_{I}\left(s\right)+F_{\mathrm{II}}\left(s\right)\right]ds+L_{12}\epsilon^{-1/2}\int_{0}^{t}e^{-(\Gamma /\epsilon )(t-s)}\sigma dW\left(s\right).$$

For simplicity in exposition, zero initial data are assumed for $u_{\mathrm{II}}$. As discussed in detail [40,109], the second and third terms in (40) simplify in the limit ϵ→0 and yield reduced simplified local stochastic dynamics for $u_{I}$ alone given by

(41) $$\frac{d{\tilde{u}}_{I}}{dt}=\left(L_{11}+\epsilon L_{12}\Gamma^{-1}L_{21}\right){\tilde{u}}_{I}+\epsilon^{1/2}L_{12}{\left(-\Gamma \right)}^{-1}\sigma \dot{W}+F_{I}+\epsilon L_{12}{\left(-\Gamma \right)}^{-1}F_{\mathrm{II}}.$$

This is an explicit example of stochastic mode reduction where the variables $u_{\mathrm{II}}$ have been eliminated and there is a reduced local stochastic equation for ${\tilde{u}}_{I}$ alone with explicit corrections that reflect the interaction with the unresolved variables. Here, we address the skill of the approximation in (41) in recovering the exact mean climate response in (39) independent of ϵ. Reasoning as discussed earlier in general below (38), the response of the climate mean in (41) to a change in forcing is given exactly by

(42) $$\delta \langle {\tilde{u}}_{I}\rangle ={\left(L_{11}+\epsilon L_{12}\Gamma^{-1}L_{21}\right)}^{-1}\delta F_{I}.$$

Remarkably, the mean response operator in (42) coincides exactly with the projected mean climate response operator in (39) for the complete stochastic system in (36) for any value of ϵ>0! This general result points to the high skill of the response for the reduced stochastic model in calculating the mean climate response. Note that the asymptotic behavior and the filtering skill of the linear multiscale stochastic model in (36) have both been studied in [115].

We wrap up this subsection with the following remark. Unlike the linear models as shown in Section 2.3 and Section 2.4, direct calculations of the response in general nonlinear models become a great challenge. Nevertheless, fluctuation–dissipation theorem (FDT) provides an efficient and practical way for computing the response in nonlinear systems. The general framework of FDT will be developed in Section 4. Note that low-frequency regimes of general circulation models (GCMs) typically exhibit subtle but systematic departures from Gaussianity. In [41], the stochastic mode reduction technique is applied to a simple prototype nonlinear stochastic model that mimics structural features of low-frequency variability of GCMs with non-Gaussian features [116]. FDT is then used to study the skill of the resulting reduced nonlinear stochastic models.

### 2.5. Fitting Autocorrelation Function of Time Series by a Spectral Information Criteria

As was seen in the previous subsections, the model sensitivity is applied to quantify the information of the temporal evolution of the system. In fact, the autocorrelation function of a given stochastic system is a simple and easily computed measurement that can be used for accessing the model sensitivity. In this subsection, we make use of the autocorrelation to quantify the model sensitivity based on a new information-theoretic framework. Autocorrelation is the correlation of a signal with a delayed copy of itself, as a function of delay. For a zero mean and stationary random process *u*, the autocorrelation function can be calculated as

(43) $$R\left(t\right)=\lim_{T\to \infty }\frac{1}{T}\int_{0}^{T}\frac{u\left(t+\tau \right)u^{*}\left(\tau \right)}{Var(u)}d\tau .$$

Clearly, a linear Gaussian process is completely determined by its mean and autocorrelation function, where the autocorrelation function characterizes the memory of the system. Therefore, an accurate estimation of the autocorrelation function in the imperfect models plays an important role in prediction. In many applications, the integral of the autocorrelation function,

(44) $$\tau =\int_{0}^{\infty }R\left(t\right)dt,$$

which is known as the decorrelation time, is used for model calibration. Although fitting the decorrelation time in the imperfect model is a simpler strategy, it is however insufficient for pointwise agreement with the true autocorrelation. In particular, if the underlying nonlinear turbulent dynamics has a slow mixing rate and involves wave-like behavior, then the profile of the true autocorrelation function is very likely to be a damped oscillation. As a consequence, fitting only the decorrelation time in the imperfect model results in a large model error due to the failure of capturing the detailed oscillation structures of the autocorrelation function, which severely deteriorates the prediction skill. Thus, it is of practical importance to calibrate the autocorrelation function in imperfect models in order to capture the dynamical features beyond the equilibrium statistics of the truth. The autocorrelation function is also directly linked with the model sensitive in terms of the mean response as well as the prediction skill.

Information theory provides a rigorous and practical way to quantify the error in the two autocorrelation functions associated with the perfect and imperfect models respectively [81,117]. However, direct application of the information distance in (1) is not suitable for measuring the difference between the two autocorrelation functions. This is because the autocorrelation function R(t) may oscillate with negative values while π and $\pi^{M}$ have to be positive in (1). To generalize the information-theoretic framework to include the autocorrelation functions, the theory of spectral representation of stationary random fields [118] is exploited here. It is proved by Khinchin’s formula [118] that if the autocorrelation function R(t) is smooth and rapid-decay, which is the typical property for most systems, then there exists a non-negative function E(λ)≥0 such that

(45) $$R\left(t\right)=\int_{-\infty }^{\infty }e^{i\lambda t}dF\left(\lambda \right),$$

with dF(λ)=E(λ)dλ a non-decreasing function. Therefore, the spectral representation of the stationary process of *u* can be constructed as

(46) $$u\left(t\right)=\int_{-\infty }^{\infty }e^{i\lambda t}\hat{Z}\left(d\lambda \right).$$

The exact spectral random measure $\hat{Z}\left(d\lambda \right)$ has independent increments whose energy spectrum can be represented by E(λ) or dF(λ)

$$dF\left(\lambda \right)=E\left(\lambda \right)d\lambda =E{\left|\hat{Z}\left(d\lambda \right)\right|}^{2}.$$

Applying the theory for spectra representation of stationary processes, an one-to-one correspondence between the autocorrelation function R(t) and non-negative energy spectra E(λ) together with the spectral representation $\hat{Z}\left(d\lambda \right)$ of the process u(t) can be found. Consider the approximation of this random process with only second order statistics by a lattice random field with spacing Δλ. By independence, the true increment $\hat{Z}\left(\Delta \lambda_{j}\right)=\hat{Z}\left(\lambda_{j}+\Delta \lambda \right)-\hat{Z}\left(\lambda_{j}\right)$ has the second order Gaussian probability density function approximation

$$\hat{Z}\left(\Delta \lambda \right)∼p_{G}\left(x;\lambda \right)\Delta \lambda =N\left(0,E\left(\lambda \right)\Delta \lambda \right),$$

and the corresponding spectral representation from the imperfect model also has the density function

$${\hat{Z}}^{M}\left(\Delta \lambda \right)∼p_{G}^{M}\left(x;\lambda \right)\Delta \lambda =N\left(0,E^{M}\left(\lambda \right)\Delta \lambda \right),$$

where $N(\mu ,\sigma^{2})$ denotes a Gaussian random variable with mean μ and variance $\sigma^{2}$. Since the spectral measure has independent increment, we approximate the true and imperfect model Gaussian random fields by

$$p_{G}=\prod_{j}N\left(0,E\left(\lambda_{j}\right)\Delta \lambda \right),\;p_{G}^{M}=\prod_{j}N\left(0,E^{M}\left(\lambda_{j}\right)\Delta \lambda \right).$$

Then, the normalized relative entropy between these two Gaussian fields becomes

$$\begin{array}{cc} P(p_{G},p_{G}^{M}) & =\sum_{j}P\left(p_{G}\left(x;\lambda_{j}\right),p_{G}^{M}\left(x;\lambda_{j}\right)\right)\Delta \lambda ,\\ & \to \int_{-\infty }^{\infty }P\left(p_{G}\left(x;\lambda \right),p_{G}^{M}\left(x;\lambda \right)\right)d\lambda ,\;\mathrm{as}\;\Delta \lambda \to 0 \end{array}$$

Therefore, given spectral density E(λ) and $E^{M}\left(\lambda \right)$, the spectral relative entropy is given by

(47) $$P\left(p_{G},p_{G}^{M}\right)=P\left(E\left(\lambda \right),E^{M}\left(\lambda \right)\right):=\int_{-\infty }^{\infty }P\left(p_{G}\left(x;\lambda \right),p_{G}^{M}\left(x;\lambda \right)\right)d\lambda ,$$

where we slightly abuse the notion above by using the spectra E(λ) to denote density functions. Since *E* and $E^{M}$ are variances for the spectral random variables, it is well-defined in the last part of the above formula (47) using the information distance formula (1). Through measuring the information distance in the spectral coefficients $\hat{Z}\left(\lambda \right)$, we arrive at the lack of information in the autocorrelation function R(t) from the model. See [81] for more details as well as an efficient algorithm of solving (47).

Finally, let there be the set of parameters θ for the imperfect model. Minimum relative entropy criterion implies the process of minimizing the lack of spectral information distance by picking the optimal parameter set $\theta^{*}$ for the imperfect model so that

(48) $$P\left(E\left(\lambda \right),E^{M}\left(\lambda ,\theta^{*}\right)\right)=\min_{\theta }P\left(E\left(\lambda \right),E^{M}\left(\lambda ,\theta \right)\right).$$

The following example makes use of the above spectral information criteria to reveal the importance in calibrating the autocorrelation function in the imperfect linear prediction model. The perfect model considered here is a noisy version of the so-called Lorenz 84 model [119,120],

(49) $$\begin{array}{cc} \frac{dx}{dt} & =-\left(y^{2}+z^{2}\right)-a\left(x-f\right)+\sigma_{x}{\dot{W}}_{x},\\ \frac{dy}{dt} & =-bxz+xy-y+g+\sigma_{y}{\dot{W}}_{y},\\ \frac{dz}{dt} & =bxy+xz-z+\sigma_{z}{\dot{W}}_{z}. \end{array}$$

This model is an extremely simple analogue of the global atmospheric circulation and the noise-free version can be derived as a Galerkin truncation of the two-layer quasigeostrophic potential vorticity equations in a channel [121]. In (49), *x* represents the intensity of the mid-latitude westerly wind current while *y* and *z* represent the cosine and sine phases of a chain of vortices superimposed on the zonal flow.

With g=0, the processes *y* and *z* in (49) form a pair of stochastic nonlinear oscillator through the skew-symmetric terms -bxz and bxy, where the frequency of the oscillation is stochastic and it depends on the amplitude of *x*. Meanwhile, *x* also plays the role of stochastic damping, which can be seen in the nonlinear terms xy and xz that modify the wave amplitudes.

The parameters used in this test are as follows:

(50) $$a=5,\;b=2,\;f=1,\;g=0,\;\mathrm{and}\;\sigma_{x}=\sigma_{y}=\sigma_{z}=0.1.$$

**Figure 8:**
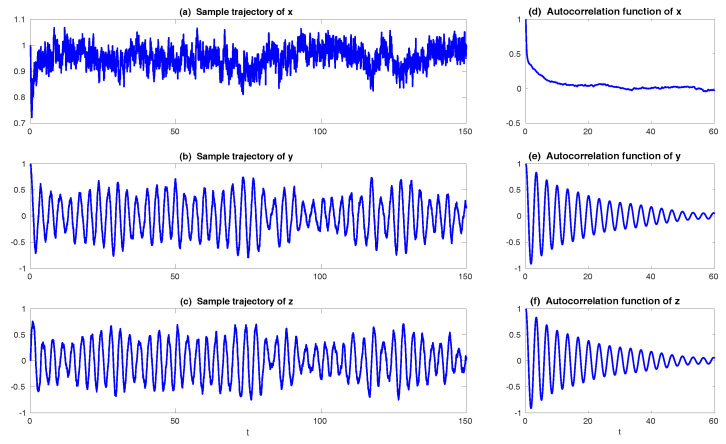
(**a**–**c**): sample trajectories of noisy Lorenz 84 model in (49); (**d**–**f**): the corresponding autocorrelation functions.

In Figure 8, sample trajectories and the corresponding autocorrelation functions associated with x,y and *z* in Equation (49) with parameters (50) are shown. It is clear that the autocorrelation functions associated with *y* and *z* oscillate and decay to zero, which satisfies the features of wave pairs.

The goal here is to predict the vortex variables *y* and *z*. The imperfect model for prediction is a mean stochastic model (MSm) with a constant phase,

(51) $$\frac{du^{M}}{dt}=\left(-d_{u}^{M}+i\omega_{u}^{M}\right)u^{M}+\sigma_{u}^{M}\dot{W}.$$

Note that $u^{M}$ is a complex process and its real and imaginary parts correspond to the pair of vortex *y* and *z* in (49), respectively. Due to the simple linear structure, the autocorrelation function $R^{M}\left(t\right)$ and spectra density $E^{M}\left(\lambda \right)$ of the MSm in (51) can be written down explicitly,

(52) $$R^{M}\left(t\right)=\exp\left((-d_{u}^{M}+i\omega_{u}^{M})t\right),\;\mathrm{and}\;E^{M}\left(\lambda \right)=\frac{2d_{u}^{M}}{{\left(d_{u}^{M}\right)}^{2}+{\left(\lambda -\omega_{u}^{M}\right)}^{2}}.$$

The model (51) has three parameters to determine: $d_{u}^{M},\omega_{u}^{M}$ and $\sigma_{u}^{M}$. Now, we apply the spectral relative entropy in (47) and make use of the analytic formula in (52) for $E^{M}\left(\lambda \right)$ to implement the optimization (48). Note that the spectra density $E^{M}\left(\lambda \right)$ in (52) does not depend on the stochastic forcing coefficient $\sigma_{u}^{M}$. Therefore, the optimization in (48) is over all the possible choices of $d_{u}^{M}$ and $\omega_{u}^{M}$. This gives the following results:

(53) $$\begin{array}{c} \mathrm{Optimal}\;\mathrm{parameters}\;\mathrm{by}\;\mathrm{fitting}\;\mathrm{the}\;\mathrm{autocorrelation}\;\mathrm{function}:\\ d_{u}^{M}=0.1,\;\mathrm{and}\;\omega_{u}^{M}=1.85. \end{array}$$

For comparison, we also adopt the traditional parameter estimation strategy in MSm by fitting only the decorrelation time (44) [15]:

(54) $$\begin{array}{c} \mathrm{Optimal}\;\mathrm{parameters}\;\mathrm{by}\;\mathrm{fitting}\;\mathrm{only}\;\mathrm{the}\;\mathrm{decorrelation}\;\mathrm{time}:\\ d_{u}^{M}=5.0,\;\mathrm{and}\;\omega_{u}^{M}=1.85. \end{array}$$

**Figure 9:**
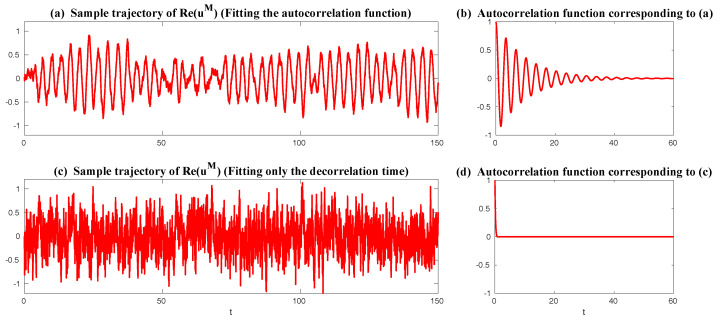
Panels (**a**,**b**): Sample trajectories and the corresponding autocorrelation functions of Re$\left(u^{M}\right)$ with parameters in (53). Panels (**c**,**d**) those with parameters in (54).

The remaining parameter $\sigma_{u}^{M}$ is calibrated by matching the variance of *y* and Re$\left(u^{M}\right)$ at the statistical steady state, which results in $\sigma_{u}^{M}=0.205$ in Case (53) and $\sigma_{u}^{M}=1.850$ in Case (54). In Figure 9, two sample trajectories of Re$\left(u^{M}\right)$ and the corresponding autocorrelation functions with parameters in (53) and (54) are shown. It is clear that the sample trajectory of Re$\left(u^{M}\right)$ and the corresponding autocorrelation function with parameters in (53) highly resemble those of *y* in Figure 8. On the other hand, the decorrelation time of *y* is very short, which is due to the canceling out of the oscillation patterns with positive and negative values by integrating the autocorrelation function. Thus, the oscillation patterns in the trajectory of Re$\left(u^{M}\right)$ are overwhelmed by the noise due to the strong mixing property when the model is calibrated using only the decorrelation time (Panels (c) in Figure 9). This example indicates the necessity of using the information criterion developed here for calibrating the autocorrelation function rather than simply matching the decorrelation time as used in many earlier works.

**Figure 10:**
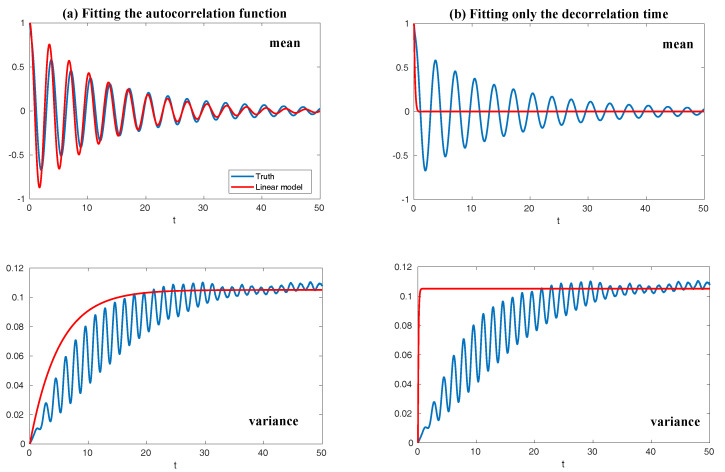
Time evolutions of mean and variance in the perfect model of *y* and imperfect model of the real part of $u^{M}$. (**a**): the parameters in the imperfect model are calibrated by matching the autocorrelation function (53); (**b**): the parameters in the imperfect model are calibrated by matching only the decorrelation time (54).

Finally, in Figure 10, we show the prediction of the time evolution of the mean and variance of *y* and Re$\left(u^{M}\right)$ starting from the same initial value y= Re$(u^{M})=1$ and others =0. In column (a), the mean evolution of Re$\left(u^{M}\right)$ from the linear model (51) captures that of *y* in the nonlinear Lorenz 84 model (49) quite accurately with a significant oscillation structure. The trend of the variance using the linear model is also quite similar to that using the Lorenz 84 model, though the oscillation structure in the variance due to the fact that the nonlinearity in the Lorenz 84 model is not predicted by the linear MSm. The latter has already been discussed in [111] and in Section 2.2.1 as an information barrier. On the other hand, by calibrating only the decorrelation time (Column (b) in Figure 10), the time evolutions of the mean and variance have dramatically fast relaxations towards the statistical steady state, which are completely different from the truth.

In [81], the information theory as shown above has been applied to calibrate more complicated reduced order models. The calibrated models succeed in predicting fat-tailed intermittent PDFs in passive scalar turbulence.

## 3. Quantifying Model Error with Information Theory in State Estimation and Prediction

### 3.1. Kalman Filter, State Estimation and Linear Stochastic Model Prediction

Filtering (also known as data assimilation or state estimation) is the process of obtaining the optimal statistical estimate (based on a Bayesian framework for example) of a natural system from partial observations of the true signal. Important contemporary examples involve the real-time filtering and prediction of weather and climate as well as the spread of hazardous plumes or pollutants [13,14,15,16,122,123,124].

**Figure 11:**
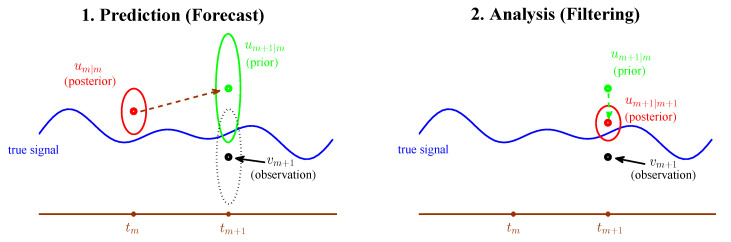
Illustration of the prediction-filtering procedure.

The general procedure of filtering complex turbulent dynamical systems with partial and noisy observations contains two steps at each time step t=mΔt. The first step involves a statistical prediction of a probability distribution $u_{m+1|m}$ starting from the initial value $u_{m|m}$ using the given dynamical model. Then, in the second step, $u_{m+1|m}$ is corrected on the basis of the statistical input of noisy observation $v_{m+1}$, which results in $u_{m+1|m+1}$. See the illustration of Figure 11.

For linear system with Gaussian noise, the above procedure is known as the Kalman filter [125,126,127]. Below, we summarize the Kalman filter for a one-dimensional complex variable [13,15,17].

Let $u_{m}\in C$ be a complex random variable whose dynamics are given by the following:

(55) $$u_{m+1}=Fu_{m}+F_{m+1}+\sigma_{m+1},$$

where $\sigma_{m+1}$ is a complex Gaussian noise with $\sigma_{m+1}=\left(\sigma_{1,m+1}+i\sigma_{2,m+1}\right)/\sqrt{2}$ and it has zero mean and variance $r=\langle \sigma_{m+1}\sigma_{m+1}^{*}\rangle =\frac{1}{2}\sum_{j=1}^{2}\langle \sigma_{j,m+1}^{2}\rangle$. Here, *F* is a complex number known as the forward operator and F is an external forcing which can vary in time. The goal of the Kalman filter is to estimate the unknown true state $u_{m+1}$, given noisy observations

(56) $$v_{m+1}=gu_{m+1}+\sigma_{m+1}^{o},$$

where *g* is a linear observation operator and $\sigma_{m}^{o}\in C$ is an unbiased Gaussian noise with variance $r^{o}=\langle \sigma_{m}^{o}{\left(\sigma_{m}^{o}\right)}^{*}\rangle$. The Kalman filter is the optimal (in the least-squares sense) solution found by assuming that the model and the observation operator that relates the model state with the observation variables are both linear and both the observation and prior forecast error uncertainties are Gaussian, unbiased and uncorrelated. In particular, the observation error distribution of *v* at time $t_{m+1}$ is a Gaussian conditional distribution

(57) $$p\left(v_{m+1}|u_{m+1}\right)∼N\left(gu_{m+1},r^{o}\right),$$

which depends on the true state $u_{m+1}$ through (55). In (57), $p(v_{m+1}|u_{m+1})$ is known as the likelihood of estimating $u_{m+1}$ given observation $v_{m+1}$.

Assume the filter model is perfectly specified [128]. An estimate of the true state prior to knowledge of the observation at time $t_{m+1}$, which is known as the prior state or forecast state, is given by

(58) $$u_{m+1|m}=Fu_{m|m}+F_{m+1}+\sigma_{m+1}.$$

See the first step in Figure 11. From the probabilistic point of view, we can represent this prior estimate with a probability density $p(u_{m+1})$. This prior distribution acounts only for the earlier observations up to time $t_{m}$,

(59) $$p\left(u_{m+1}\right)∼N\left({\bar{u}}_{m+1|m},r_{m+1|m}\right),$$

where the prior mean and prior variance

(60) $$\begin{array}{cc} {\bar{u}}_{m+1|m} & \equiv \langle u_{m+1|m}\rangle ,\\ r_{m+1|m} & \equiv \langle \left(u_{m+1}-{\bar{u}}_{m+1|m}\right){\left(u_{m+1}-{\bar{u}}_{m+1|m}\right)}^{*}\rangle , \end{array}$$

are solved via

(61) $$\begin{array}{cc} {\bar{u}}_{m+1|m} & =F{\bar{u}}_{m|m}+F_{m+1},\\ r_{m+1|m} & =Fr_{m|m}F^{*}+r, \end{array}$$

with $r_{m|m}=\langle \left(u_{m}-{\bar{u}}_{m|m}\right){\left(u_{m}-{\bar{u}}_{m|m}\right)}^{*}\rangle$. Note that in order to solve the prior distribution $p(u_{m+1|m})$, the posterior information in the previous step ${\bar{u}}_{m|m},r_{m|m}$ has been used.

Next, we derive the posterior state (or the filtered state) that combines the prior information $p(u_{m+1|m})$ with the observation $v_{m+1}$ at $t_{m+1}$. This estimate is given in the probabilistic sense by the Bayesian update through maximizing the following conditional density,

(62) $$p\left(u_{m+1}|v_{m+1}\right)∼p\left(u_{m+1}\right)p\left(v_{m+1}|u_{m+1}\right)=e^{-\frac{1}{2}J\left(u_{m+1}\right)},$$

which is equivalent to minimizing

$$J\left(u\right)=\frac{{\left(u-{\bar{u}}_{m+1|m}\right)}^{*}\left(u-{\bar{u}}_{m+1|m}\right)}{r_{m+1|m}}+\frac{{\left(v_{m+1}-gu\right)}^{*}\left(v_{m+1}-gu\right)}{r^{o}}.$$

The value of *u* at which J(u) attains its minimum is the estimate for the mean and is given by

(63) $${\bar{u}}_{m+1|m+1}=\left(1-K_{m+1}g\right){\bar{u}}_{m+1|m}+K_{m+1}v_{m+1},$$

where

(64) $$K_{m+1}=\frac{gr_{m+1|m}}{r^{o}+g^{2}r_{m+1|m}}$$

is the Kalman gain. Note that $0\leq K_{m+1}g\leq 1$. The filter fully weighs to the model or prior forecast when $K_{m+1}g=0$ and fully weighs to the observation when $K_{m+1}g=1$. Such weights depend on the ratio of the uncertainty (reflected by the noise) in the observations and the model. Finally, the posterior variance is calculated via the following:

(65) $$\begin{array}{cc} u_{m+1}-{\bar{u}}_{m+1|m+1} & =u_{m+1}-{\bar{u}}_{m+1|m}-K_{m+1}\left(v_{m+1}-gu_{m+1}-g\left({\bar{u}}_{m+1}-u_{m+1}\right)\right)\\ e_{m+1|m+1} & =\left(1-K_{m+1}g\right)e_{m+1|m}-K_{m+1}\sigma_{m+1}^{o}. \end{array}$$

These result in the expression of the posterior variance

(66) $$r_{m+1|m+1}=\left(1-K_{m+1}g\right)r_{m+1|m}.$$

Note that the above Kalman filter is designed for a linear system with Gaussian noise. In practice, different generalizations of the Kalman filter and various nonlinear filters such as the ensemble Kalman filter, particle filter and blended filtering techniques are applied to nonlinear and non-Gaussian systems. See [13,14,15,16,17,101,129,130] for more details.

### 3.2. Asymptotic Behavior of Prediction and Filtering in One-Dimensional Linear Stochastic Models with Model Error

Recall in Section 3.1 that the true underlying linear stochastic model is given by

(67) $$u_{m+1}=Fu_{m}+F_{m+1}+\sigma_{m+1}.$$

However, the true underlying dynamics is typically unknown in practice. Therefore, imperfect forecast models are used in the prediction stage. Now, let’s assume the forecast model has the following form

(68) $$u_{m+1}^{M}=F^{M}u_{m}^{M}+F_{m+1}^{M}+\sigma_{m+1}^{M},$$

where the model error comes from the imperfect forward operator, forcing and noise coefficient. Due to the appearance of such model errors, the updates of prediction and filtering distributions become

(69) $$\begin{array}{cc} {\bar{u}}_{m+1|m}^{M} & =F^{M}\left(1-gK_{m}^{M}\right){\bar{u}}_{m|m-1}^{M}+F^{M}K_{m}^{M}gu_{m}+F^{M}K_{m}^{M}\sigma_{m}^{o}+F_{m+1}^{M},\\ r_{m+1|m}^{M} & =\left(1-K_{m}^{M}g\right){\left|F^{M}\right|}^{2}r_{m|m-1}^{M}+r^{M}, \end{array}$$

and

(70) $$\begin{array}{cc} {\bar{u}}_{m+1|m+1}^{M} & =F^{M}\left(1-gK_{m}^{M}\right){\bar{u}}_{m|m}^{M}+F^{M}K_{m+1}^{M}gu_{m+1}+F^{M}K_{m+1}^{M}\sigma_{m+1}^{o}+F_{m+1}^{M},\\ r_{m+1|m+1}^{M} & =\left(1-K_{m}^{M}g\right)\left(\right|F^{M}{|}^{2}r_{m|m}^{M}+r^{M}), \end{array}$$

respectively.

Now, we study the asymptotic behavior of the updates (69) and (70) with model error compared with the truth based on (67). The detailed calculations are included in Appendix C, which exploit the augmented system involving the truth and the prediction/filtering with model error [31]. Here, we summarize the results.

From (67) and (68), it is easy to show the equilibrium mean estimates of the perfect and imperfect model

(71) $${\bar{u}}_{eq}=\frac{F_{\infty }}{1-F},\;{\bar{u}}_{eq}^{M}=\frac{F_{\infty }^{M}}{1-F^{M}}.$$

#### 3.2.1. Prediction

The asymptotic prediction mean of ${\bar{u}}_{m+1|m}^{M}$ is given by

(72) $$\lim_{m\to \infty }E\left({\bar{u}}_{m+1|m}^{M}\right)=\frac{1}{\left(1-F^{M}\right)+F^{M}K_{\infty }^{M}g}\left(F^{M}K_{\infty }^{M}g{\bar{u}}_{eq}+\left(1-F^{M}\right){\bar{u}}_{eq}^{M}\right).$$

Clearly, the asymptotic mean of the prediction state is a linear combination of the equilibrium mean of original true model ${\bar{u}}_{eq}$ and that of the forecast model of the mean ${\bar{u}}_{eq}^{M}$. With (67), the error in the asymptotic prediction mean of ${\bar{u}}_{m+1|m}^{M}$ yields,

(73) $$\lim_{m\to \infty }E\left(u_{m+1}-{\bar{u}}_{m+1|m}^{M}\right)=\frac{1-F^{M}}{\left(1-F^{M}\right)+F^{M}K_{\infty }^{M}g}\left({\bar{u}}_{eq}-{\bar{u}}_{eq}^{M}\right).$$

The asymptotic prediction mean of ${\bar{u}}_{m+1|m}^{M}$ is equal to the equilibrium mean of the perfect model if and only if the imperfect model has the same equilibrium mean as the perfect model, namely ${\bar{u}}_{eq}={\bar{u}}_{eq}^{M}$.

On the other hand, the asymptotic prediction variance $r_{\infty }^{P}=\lim_{m\to \infty }r_{m+1|m}$ is given by

(74) $$r_{\infty }^{P}=|F^{M}{|}^{2}\left(1-K_{\infty }^{M}g\right)r_{\infty }^{P}+r^{M}={\left|F^{M}\right|}^{2}\frac{r^{o}r_{\infty }^{P}}{g^{2}r_{\infty }^{P}+r^{o}}+r^{M},$$

where the asymptotic Kalman gain is

$$K_{\infty }^{M}=\frac{gr_{\infty }^{P}}{g^{2}r_{\infty }^{P}+r^{o}}.$$

Therefore, the asymptotic prediction variance simplifies to

(75) $$r_{\infty }^{P}=\frac{|F^{M}{|}^{2}r^{o}}{g}K_{\infty }^{M}+r^{M}.$$

#### 3.2.2. Filtering

The asymptotic filtering mean of ${\bar{u}}_{m+1|m+1}^{M}$ is given by

(76) $$\lim_{m\to \infty }{\bar{u}}_{m+1|m+1}^{M}=\frac{1}{\left(1-F^{M}\right)+F^{M}K_{\infty }^{M}g}\left(K_{\infty }^{M}g{\bar{u}}_{eq}+\left(1-F^{M}\right)\left(1-K_{\infty }^{M}g\right){\bar{u}}_{eq}^{M}\right)$$

with (67), the error in the asymptotic filtering mean of ${\bar{u}}_{m+1|m+1}^{M}$ yields,

(77) $$\lim_{m\to \infty }E\left(u_{m+1}-{\bar{u}}_{m+1|m+1}\right)=\frac{\left(1-F^{M}\right)\left(1-K_{\infty }^{M}g\right)}{\left(1-F^{M}\right)+F^{M}K_{\infty }^{M}g}\left({\bar{u}}_{eq}-{\bar{u}}_{eq}^{M}\right).$$

The asymptotic prediction mean of ${\bar{u}}_{m+1|m+1}^{M}$ is equal to the equilibrium mean of the perfect model provided that (1) the imperfect model has the same equilibrium mean as the perfect model, namely ${\bar{u}}_{eq}={\bar{u}}_{eq}^{M}$, or (2) $K_{\infty }^{M}g=1$, namely the observational noise is zero and the filter trusts completely towards the observations.

On the other hand, the dynamics of prediction variance $r_{\infty }^{A}=\lim_{m\to \infty }r_{m+1|m+1}$ are given by

(78) $$r_{\infty }^{A}=\left(1-K_{m+1}^{M}g\right)\left(\right|F^{M}{|}^{2}r_{\infty }^{A}+r^{M})$$

with direct manipulations, the asymptotic analysis variance becomes

(79) $$r_{\infty }^{A}=\frac{r^{o}}{g}K_{\infty }^{M}.$$

#### 3.2.3. Comparison

Comparing the asymptotic prediction mean (73) and filtering mean (77), we have

(80) $$\lim_{m\to \infty }E\left(u_{m+1}-{\bar{u}}_{m+1|m+1}\right)=\left(1-K_{\infty }^{M}g\right)\lim_{m\to \infty }E\left(u_{m+1}-{\bar{u}}_{m+1|m}\right),$$

which indicates that the error in the filtering mean is always smaller than that in the prediction mean in the sense of standard mean deviation, with the existence of observational error.

Next, comparing the asymptotic prediction variance (75) and filtering variance (79), we have

(81) $$r_{\infty }^{A}-r_{\infty }^{P}=\frac{r^{o}}{g}K_{\infty }^{M}-\frac{|F^{M}{|}^{2}r^{o}}{g}K_{\infty }^{M}-r^{M}=\left(1-\right|F^{M}{|}^{2})r_{\infty }^{P}-r^{M}<0.$$

See Appendix C for the detailed derivations. This implies that filtering state always results in a smaller uncertainty (variance) than the prediction state. Such an uncertainty reduction is due to the extra information in the noisy observations.

Note that the conclusions made from (80) and (81) are valid only when full observations are available. In high dimensional situations, if the observations are only available on part of the variables (known as partial observations), then the prediction error can be smaller than filtering error. See Section 3.5.1 for simple examples.

### 3.3. An Information Theoretical Framework for State Estimation and Prediction

#### 3.3.1. Motivation Examples

To illustrate the importance and necessity of developing an information theoretical framework in assessing the filtering and predicting skill, let us first review the two traditional path-wise measurements that are widely used in filtering and prediction [13,131,132,133,134,135]. Denote $u_{i}$, i=1,…,n the true signal and ${\hat{u}}_{i}$ the filtering/prediction estimate. These measurements are given by

1. The root-mean-square error (RMSE):
  (82) $$RMSE=\sqrt{\frac{\sum_{i=1}^{n}{\left({\hat{u}}_{i}-u_{i}\right)}^{2}}{n}}.$$
2. The pattern correlation (PC):
  (83) $$PC=\frac{\sum_{i=1}^{n}\left({\hat{u}}_{i}-{\bar{\hat{u}}}_{i}\right)\left(u_{i}-{\bar{u}}_{i}\right)}{\sqrt{\sum_{i=1}^{n}{\left({\hat{u}}_{i}-{\bar{\hat{u}}}_{i}\right)}^{2}}\sqrt{\sum_{i=1}^{n}{\left(u_{i}-{\bar{u}}_{i}\right)}^{2}}},$$
  where ${\bar{\hat{u}}}_{i}$ and ${\bar{u}}_{i}$ denotes the mean of ${\hat{u}}_{i}$ and $u_{i}$, respectively.

While these two path-wise measurements are easy to implement and are able to quantify the filtering/prediction skill to some extent, they have fundamental limitations. To see this, let us consider a simple motivation example, where the true dynamics is given by

(84) $$\frac{du}{dt}=-\gamma u+f_{0}+f_{1}e^{i\omega_{1}t}+\sigma \dot{W}.$$

with parameters

(85) $$\gamma =1,\;f_{0}=0,\;f_{1}=1,\;\omega_{1}=1,\;\sigma =2.$$

For the imperfect forecast model, we assume the same ansatz as the prefect one in (84) but the parameters related to the forcing amplitudes contain model errors. Consider the following two imperfect models:

(86) $$\begin{array}{cc} \mathrm{Imperfect}\;\mathrm{forecast}\;\mathrm{model}\;(a):\; & \gamma =1,\;f_{0}^{M}=0,\;f_{1}^{M}=0,\;\omega_{1}=1,\;\sigma =2,\\ \mathrm{Imperfect}\;\mathrm{forecast}\;\mathrm{model}\;(b):\; & \gamma =1,\;f_{0}^{M}=0,\;f_{1}^{M}=2,\;\omega_{1}=1,\;\sigma =2. \end{array}$$

**Figure 12:**
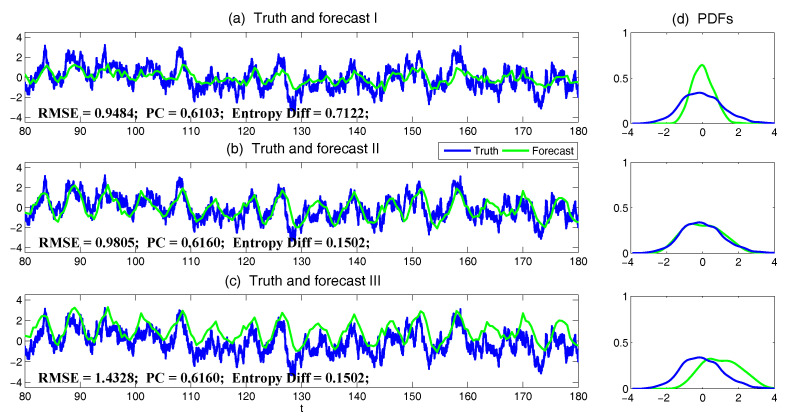
Motivation examples for the limitations of assessing the prediction error based only the Root-mean-square error (RMSE) and the pattern correlation (**a**,**b**) and based only on the Shannon entropy difference (**b**,**c**). Here, the truth is the same in (**a**–**c**), which is generated from (84) and (85). The three imperfect forecast models are given by (86) and (89). In column (**d**), the associated PDFs are shown. In all the panels, only the real part of *u* is shown.

In panels (a) and (b) of Figure 12, we show the predictions using these two imperfect forecast model (green curve) and they are compared with the truth (blue curve). Here, the observational time step is $\Delta t_{obs}=0.5$ which is less than the decorrelation time τ=1/γ=1, and the observational noise level is $r^{o}=1$. In terms of the RMSE and PC, the two predictions are comparable with each other and the one in panel (a) is even slightly more skillful. However, the prediction in panel (a) by intuition is worse than that in panel (b). In fact, the amplitude of the prediction using the imperfect model (a) is severely underestimated. The consequence is that the prediction fails to capture all the important extreme events in the true signal. On the other hand, the prediction using the imperfect model (b) results in a time series which has the same amplitude as the truth. See the PDFs associated with the time series in panel (d) for estimating the amplitudes. Therefore, the two traditional path-wise measurements—RMSE and PC—are misleading here in providing the prediction skill. In addition, according to the definitions of the RMSE and the PC in (82) and (83), both the measurements take into account the information only up to the second-order statistics. Therefore, they are not able to capture the information beyond Gaussian statistics and are not suitable to assess the filtering/prediction skill for any non-Gaussian models as in nature.

Due to the above fundamental limitations of these two traditional path-wise measurements, various information measurements become useful in assessing the filtering/predictino skill. In [34,56,136,137,138], an information measurement called Shannon entropy difference was introduced and was used to assess the filtering/prediction skill. The Shannon entropy difference is defined as

(87) $$S\left(\pi \right)-S\left(\pi^{M}\right)=-\int \pi \log\pi +\int \pi^{M}\log\pi^{M}.$$

In particular, if both $\pi \equiv \pi_{G}$ and $\pi^{M}\equiv \pi_{G}^{M}$ are Gaussian (as in the linear models), then the Shannon entropy difference has the following explicit formula:

(88) $$\begin{array}{cc} S\left(\pi_{G}\right)-S\left(\pi_{G}^{M}\right) & =\left(\frac{1}{2}\log\det R+\frac{1}{2}\left(1+\log\left(2\pi \right)\right)\right)-\left(\frac{1}{2}\log\det R^{M}+\frac{1}{2}\left(1+\log\left(2\pi \right)\right)\right)\\ & =\frac{1}{2}\log\det\left(RR^{M}\right), \end{array}$$

where *R* and $R^{M}$ are the covariance of $\pi_{G}$ and $\pi_{G}^{M}$. Intuitively, the Shannon entropy difference quantifies the uncertainty between π and $\pi^{M}$. For Gaussian distributions, the uncertainty is reflected by the variance. Connecting the Shannon entropy difference with the two predictions in panels (a) and (b) of Figure 12, it is expected that the Shannon entropy difference is able to distinguish the two predictions since the associated PDFs of the two predictions have different variances. In fact, the Shannon entropy difference in the imperfect model (a) (0.7122) is much larger than that in the imperfect model (b) (0.1502), which indicates that the prediction in panel (b) is more skillful than that in panel (a).

However, relying solely on the Shannon entropy difference in assessing the filtering/prediction skill is also misleading. Consider an imperfect model with the following parameters:

(89) $$\mathrm{Imperfect}\;\mathrm{forecast}\;\mathrm{model}\;\left(c\right):\;\gamma =1,\;f_{0}^{M}=2,\;f_{1}^{M}=2,\;\omega_{1}=1,\;\sigma =2.$$

Comparing with the perfect model and the other two imperfect models in (86), a non-zero constant forcing $f_{0}^{M}=2$ is imposed in (89). The prediction results are shown in panel (c) of Figure 12. Since the Shannon entropy difference in Gaussian framework (88) takes into account only the the variance but completely ignores the information in the mean, the resulting Shannon entropy difference using the imperfect models (b) and (c) give exactly the same value. In addition, the pattern correlations in these two models are also identical to each other. However, the prediction using the imperfect model (c) has an obvious mean bias and therefore the prediction is not as skillful as that using the imperfect model (b).

From these simple motivation examples, it seems that the combination of the RMSE, the PC and the Shannon entropy difference can overcome the fundamental limitations as discussed above. However, there are at least two extra shortcomings even in the combination of these three measurements. First, two different PDFs associated with the imperfect model, namely $\pi_{\left\{1\right\}}^{M}$ and $\pi_{\left\{2\right\}}^{M}$, may result in the same Shannon entropy difference compared with the truth. For example, such situation happens when $\pi_{\left\{2\right\}}^{M}$ has a mean shift compared with $\pi_{\left\{1\right\}}^{M}$. This is because the Shannon entropy difference computes the uncertainty of the two distributions separately rather than considering the pointwise difference between the two PDFs. Therefore, a more sophisticated measurement should take into account the relative difference between the PDFs associated with the perfect and imperfect models. Second, as has been discussed above, the RMSE and PC only make use of the information up to the second order statistics. The important non-Gaussian features as they appeared in many realistic applications are not reflected in these path-wise measurements.

#### 3.3.2. Assessing the Skill of Estimation and Prediction Using Information Theory

Due to the fundamental limitations in the two classical path-wise measurement, RMSE and PC, as well as those in the Shannon entropy difference, a new information-theoretic framework [102] has developed to assess the filtering/prediction skill. Again, denote π≡π(u) and $\pi^{M}\equiv \pi \left(u^{M}\right)$ the PDFs associated with truth *u* and the filtering/prediction estimate $u^{M}$, respectively. Denote $p(u,u^{M})$ the joint PDF of *u* and $u^{M}$. Let $U=u-u^{M}$ be the residual between the truth and the estimate. This information-theoretic framework involves three information measurements:

1. The Shannon entropy residual,
  (90) S(U)=-∫p(U)logp(U).
2. The mutual information,
  (91) $$M\left(\pi ,\pi^{M}\right)=\;\int \int \;p\left(u,u^{M}\right)\log\left(\frac{p(u,u^{M})}{\pi \left(u\right)\pi \left(u^{M}\right)}\right).$$
3. The relative entropy,
  (92) $$R\left(\pi ,\pi^{M}\right)=-\int \pi \log\left(\frac{\pi }{\pi^{M}}\right).$$

The Shannon entropy residual quantifies the uncertainty in the point-wise difference between *u* and $u^{M}$. It is an information surrogate of the RMSE in the Gaussian framework. The mutual information quantifies the dependence between the two processes. It measures the lack of information in the factorized density $\pi \left(u\right)\pi \left(u^{M}\right)$ relative to the joint density $p(u,u^{M})$, which follows the identity,

(93) $$M\left(\pi ,\pi^{M}\right)=P\left(p\left(u,u^{M}\right),\pi \left(u\right)\pi \left(u^{M}\right)\right).$$

The mutual information is an information surrogate of the PC in the Gaussian framework. On the other hand, the relative entropy quantifies the lack of information in $\pi^{M}$ related to π and it is a good indicator of the skill of $u^{M}$ in capturing the peaks and extreme events of *u*. It also takes into account the pointwise discrepancy between π and $\pi^{M}$ rather than only computing the difference between the uncertainties associated with the two individual PDFs (as in the Shannon entropy difference). Therefore, the combination of these three information measurements is able to capture all the features in assessing the filtering/prediction skill and overcomes the shortcomings as discussed in the previous subsection.

Note that when $\pi ∼N(\bar{u},R)$ and $\pi^{M}∼N\left({\bar{u}}^{M},R_{M}\right)$ are both Gaussian, then the above three information measurements have explicit expressions:

1. The Shannon entropy residual (Gaussian framework),
  (94) $$S\left(U\right)=\frac{1}{2}\log\det\left(R+R^{M}-2Re\left[Cov\left(u,u^{M}\right)\right]\right).$$
2. The mutual information (Gaussian framework),
  (95) $$M\left(\pi ,\pi^{M}\right)=-\frac{1}{2}\log\det\left(I-R_{M}^{-1}Cov^{*}\left(u,u^{M}\right)R^{-1}Cov\left(u,u^{M}\right)\right).$$
3. The relative entropy (Gaussian framework),
  (96) $$R\left(\pi ,\pi^{M}\right)=\frac{1}{2}{\left(\bar{u}-{\bar{u}}^{M}\right)}^{*}R_{M}^{-1}\left(\bar{u}-{\bar{u}}^{M}\right)+\frac{1}{2}\left(-\log\det\left(RR_{M}^{-1}\right)+tr\left(RR_{M}^{-1}\right)-N\right).$$

In (94)–(96), *N* is the dimension of π and $\pi^{M}$, *I* is the identity matrix with size N×N, and $Cov(u,u^{M})$ is the covariance between *u* and $u^{M}$. More discussions of Gaussian and non-Gaussian cases can be found in [56] and [54], respectively.

The information-theoretic framework (90)–(92) or (94)–(96) is usually defined in the super-ensemble sense [31] in accessing the data assimilation and prediction skill of the imperfect model given the perfect one. However, in some more realistic situations, although the imperfect models can be run in the ensemble mode, the ensemble run of the perfect model or the truth is never available. This is because the perfect model that describes nature is unknown. The only available information is one realization from observations (e.g., satellites). Nevertheless, the information-theoretic framework can also be used in a path-wise way, where the statistics are computed by collecting all the sample points in the given realization. Some realistic applications of the information-theoretic framework for filtering and prediction can be found in [31,61,139].

### 3.4. State Estimation and Prediction for Complex Scalar Forced Ornstein–Uhlenbeck (OU) Processes

Now, we study the state estimation (filtering) and prediction. The focus here is a complex scalar forced Ornstein–Uhlenbeck (OU) process,

(97) $$\frac{du}{dt}=\left(-\gamma +i\omega_{0}\right)u+f_{0}+f_{1}e^{i\omega_{1}t}+\sigma \dot{W},$$

where γ and $\omega_{0}$ are the damping and oscillation frequency while $f_{0}$ and $f_{1}e^{i\omega_{1}t}$ are a constant and time-periodic large-scale forcing, respectively, and $\sigma \dot{W}$ is stochastic noise. Despite the simplicity of this model in (97), it can be used to mimic some climate physics [15,120]. For example, the deterministic forcing $f_{0}+f_{1}e^{i\omega_{1}t}$ can be regarded as the annual cycle while $\omega_{0}$ can be treated as internal oscillation which may occur in the intreaseasonal time scale. The damping term γ measures the system memory and the stochastic term represents the input to the system from small or unresolved scales. The model in (97) can also be regarded as one Fourier mode of complex spatially-extended systems.

The information-theoretic framework developed above will be used to assess the filtering/prediction skill and quantify the model error. The complex scalar forced OU process in (97) will be used to generate the true signal. The imperfect forecast model has the same structure as (97) but with model errors in the parameters. The goal here is to systematically study the model error as functions of the observational time step, observational noise and the forcing amplitude.

The exact solution of (97) can be written down explicitly,

(98) $$\begin{array}{cc} u(t) & =u\left(t_{0}\right)e^{\left(-\gamma +i\omega_{0}\right)\left(t-t_{0}\right)}+\frac{f_{0}}{\gamma -i\omega_{0}}\left(1-e^{\left(-\gamma +i\omega_{0}\right)\left(t-t_{0}\right)}\right)\\ & \;\;+\frac{f_{1}e^{i\omega_{1}t}}{\gamma +i(-\omega_{0}+\omega_{1})}\left(1-e^{-\left(\gamma +i\omega_{1}-i\omega_{0}\right)\left(t-t_{0}\right)}\right)+\sigma \int_{t_{0}}^{t}e^{\left(-\gamma +i\omega_{0}\right)\left(t-s\right)}dW\left(s\right), \end{array}$$

which provides the analytical forms of the time evolution of the forecast mean $\bar{u}\left(t\right)$ and the forecast variance r(t),

(99) $$\begin{array}{cc} \bar{u}\left(t\right) & =\bar{u}\left(t_{0}\right)e^{\left(-\gamma +i\omega_{0}\right)\left(t-t_{0}\right)}+\frac{f_{0}}{\gamma -i\omega_{0}}\left(1-e^{\left(-\gamma +i\omega_{0}\right)\left(t-t_{0}\right)}\right)\\ & \;\;\;\;\;\;+\frac{f_{1}e^{i\omega_{1}t}}{\gamma +i(-\omega_{0}+\omega_{1})}\left(1-e^{-\left(\gamma +i\omega_{1}-i\omega_{0}\right)\left(t-t_{0}\right)}\right),\\ r(t) & =r\left(t_{0}\right)e^{-2\gamma (t-t_{0})}+\frac{\sigma^{2}}{2\gamma }\left(1-e^{-2\gamma (t-t_{0})}\right). \end{array}$$

With the explicit expressions in (98) and (99), it is easy to write down the corresponding operators $F,F_{m+1}$ and $\sigma_{m+1}$ in (67) or those in the imperfect forecast model (68) in each prediction/filtering assimilation step.

To understand the prediction and filtering skill of the complex scalar forced OU process (97), we start with a simple case which involves only a constant forcing. We also adopt the perfect forecast model in this example. The parameters of (97) are given as follows:

(100) $$\gamma =0.4,\;\omega_{0}=1,\;f_{0}=2,\;f_{1}=0,\;\omega_{1}=0,\;\sigma =1,$$

and the observational operator g=1. Here, we take $\Delta t_{obs}=0.5$ and $r^{o}=0.5$ as the default values of the observational time step and the observational noise level, respectively. Since the phase $\omega_{0}=1$ is nonzero, the autocorrelation function has an oscillation structure. Therefore, the decorrelation time which includes the cancellation of the positive and negative values in the autocorrelation function may be misleading for measuring the system memory. Here, we have checked both the standard decorrelation time, namely the integral of the autocorrelation function (ACF), and the integral of the absolute value of the ACF. These two quantities have values $\tau_{ACF}=0.34$ and $\tau_{\left|ACF\right|}=1.63$. Thus, $\Delta t_{obs}=0.5$ is a reasonable observational time step that includes some memory of the information in the previous assimilation step. On the other hand, $r^{o}=0.5$ here results in a polluted signal with roughly 10% observational noise.

**Figure 13:**
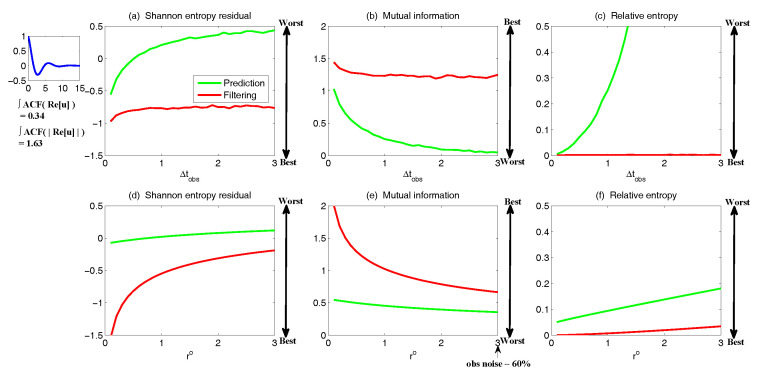
The three information measurements, namely the Shannon entropy residual, the mutual information and the relative entropy, as a function of $\Delta t_{obs}$ (**a**–**c**) and $r^{o}$ (**d**–**f**). Here, the experiments are based on the perfect model (97) with parameters in (100). The left small panel shows the autocorrelation function of Re[u].

Figure 13 show the prediction and filtering skill in terms of the three information measurements, namely the Shannon entropy residual, the mutual information and the relative entropy, as a function of $\Delta t_{obs}$ (panels (a)–(c)) and $r^{o}$ (panels (d)–(f)), respectively. First, the filtering estimate is always more skillful than the prediction one. This is consistent with the theoretical analysis in Section 3.2 in that the filter estimate combines the prediction result with the information observation and the error is therefore reduced. Next, it is clear from all the panels in Figure 13 that with the increase of either $\Delta t_{obs}$ or $r^{o}$, both the prediction and filtering skill deteriorates, which is as expected. Nevertheless, the prediction skill decreases more quickly with the increase of the observational time step $\Delta t_{obs}$. In particular, the model error in terms of the relative entropy increases exponentially. As comparison, the filtering skill only has a slight deterioration with the change of $\Delta t_{obs}$. On the other hand, the error in the filter estimate increases quickly with the observational noise $r^{o}$ when $r^{o}$ is small. When $r^{o}$ becomes moderate to large, the error in the filter estimate increases steadily. The error in the prediction estimate always increases steadily as a function of $r^{o}$.

**Figure 14:**
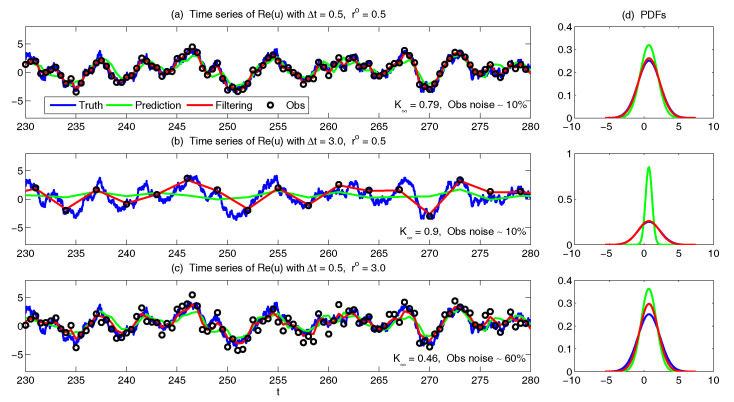
Comparison of the time series of the truth, the prediction estimate and the filter estimate. (**a**): $\Delta t_{obs}=0.5,r^{o}=0.5$; (**b**): $\Delta t_{obs}=3.0,r^{o}=0.5$; (**c**): $\Delta t_{obs}=0.5,r^{o}=3.0$; (**d**): the associated PDFs. Here, the experiments are based on the perfect model (97) with parameters in (100).

To have a more intuitive understanding of these results, the time series of the truth, the filter estimate and the prediction estimate are shown in Figure 14 with three different $\Delta t_{obs}$ or $r^{o}$. Comparing with panels (a) and (b), it is clear that a long observational time step $\Delta t_{obs}$ leads to a smaller fluctuation of the prediction estimate around its steady state mean value. In fact, the signal due to the memory effect in the previous assimilation step is strongly damped with a long observational time step and the resulting signal is dominated by the constant forcing. The consequence is that the PDF associated with the predicted time series has a much smaller variance compared with the truth and the prediction fails to capture the extreme events and large variabilities in the true signal. On the other hand, due to the incorporation of the information from the observations, the filter estimate even with a long observational time step provides a quite skillful result in terms of both the correlation and the signal amplitude. Note that the asymptotic Kalman gain in the filtering in panel (b) is $K_{\infty }=0.9$, which means the observations play an important role in regaining the skill in the filter estimate. In panel (c), the observational time step $\Delta t_{obs}=0.5$ is the same as that in panel (a) but the observational noise level is increased from $r^{o}=0.5$ to $r^{o}=3$. Both the filtering and prediction skill becomes worse as compared with those in panel (a). Nevertheless, the deterioration is not quite significant which is consistent with the statistics shown in Figure 13.

Next, we consider the complex forced scalar system with a time-periodic forcing in (97). The parameters are as follows:

(101) $$\gamma =0.4,\;f_{0}=0,\;f_{1}=2,\;\omega_{1}=1,\;\sigma =2,$$

and the observational operator g=1. Two dynamical regimes are studied here:

(102) $$RegimeI:\;\omega_{0}=0.5,\;\mathrm{and}\;RegimeII:\;\omega_{0}=1.$$

**Figure 15:**
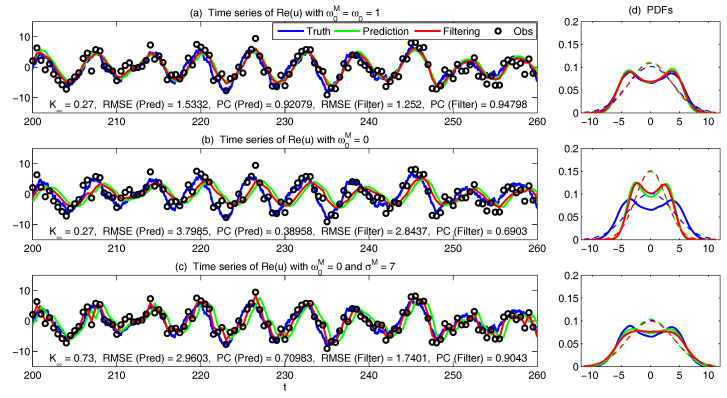
The true signal and filter and prediction estimates. (**a**): perfect model simulation; (**b**): imperfect forecast model with $\omega_{0}^{M}=0\neq 1=\omega_{0}$; (**c**): imperfect forecast model with $\omega_{0}^{M}=0\neq 1=\omega_{0}$ and optimized noise coefficient $\sigma^{M}=7$. Here, the true model is given by (97) and the parameters are shown in (101) and (102); (**d**): the associated PDFs formed by directly collecting all the points in the time series (solid curves) and the Gaussian fits (dashed curves).

It is important to note that, in Regime II, the phase $\omega_{0}$ and the forcing period $\omega_{1}$ are equal to each other, which means this dynamical regime has a resonance forcing. On the other hand, the dynamical Regime I has a non-resonance forcing. We take $\Delta t_{obs}=0.5$ and $r^{o}=9$ as the default values of the observational time step and the observational noise level, respectively. Note that although $r^{o}=9$ is much larger than that in the previous example, the signal amplitude due to the periodic forcing also increases. The observational noise here is about 25% compared with the true signal. See Figure 15 for the true signal and the noisy observations.

**Figure 16:**
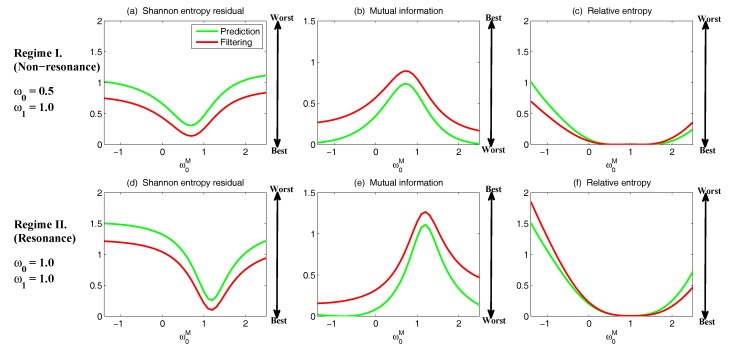
The three information measurements, namely the Shannon entropy residual, the mutual information and the relative entropy, as a function of $\omega_{0}^{M}$ in the imperfect model. The information measures are given using the Gaussian approximation (94)–(96) and the statistics here are averaged directly over the time series. (**a**–**c**): Regime I (the non-resonance regime); (**d**–**f**): Regime II (the resonance regime). The true model is given by (97) and the parameters are shown in (101) and (102). The imperfect model has the same structure and the same other parameters expect $\omega_{0}^{M}$.

**Figure 17:**
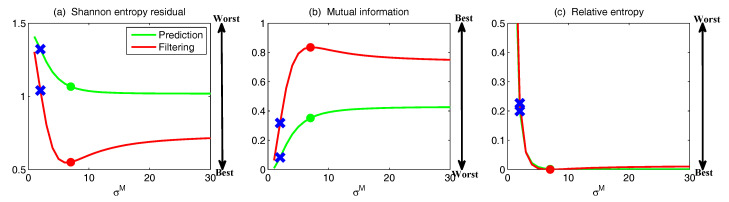
Model error as a function of $\sigma^{M}$ in the imperfect model where $\omega_{0}^{M}=0\neq 1=\omega_{0}$. The blue ’x’ shows the non-optimized values $\sigma^{M}=\sigma =2$. The dot $\sigma^{M}=7$ indicates the optimal value for the filter estimate which is also nearly the optimal value for the prediction estimate. (**a**) Shannon entropy residual, (**b**) Mutual information, (**c**) Relative entropy.

Now, let us assume the imperfect model shares the same model structure as the perfect one in (97). The imperfect part comes from the parameter $\omega_{0}^{M}$. This comes from the motivation that the situation that the large-scale forcing $f_{0}+f_{1}e^{i\omega_{1}t}$ is in general known quite well but measuring the internal oscillation $\omega_{0}$ usually contains error. In Figure 16, we show the model error in terms of the three information measurements, namely the Shannon entropy residual, the mutual information and the relative entropy, as a function of $\omega_{0}^{M}$. Here, the information measurements are computed based on the Gaussian framework (94)–(96) due to their simplicity from a practical point of view, where the statistics here are averaged directly over the time series. Note that such direct average results in a bimodal distribution in the true model due to the large amplitude of the periodic forcing such that the mixing and ergodicity are not satisfied [108] (see Figure 15 and a detailed discussion in Appendix D). Nevertheless, the Gaussian approximation in the information measurements here provides a qualitatively accurate estimate of the model error as can be seen in Figure 15, Figure 16 and Figure 17. There is an alternative way of computing the model error by first collecting all the points $t^{\prime }+mT$, m=1,2,… with $t^{\prime }$ fixed and *T* being the period of the time series. These points appear in the same location within a period and the collection forms a Gaussian distribution. Compute the information measurements for these Gaussian distributions. Then, let $t^{\prime }$ vary within $t^{\prime }\in \left(0,T\right]$ and repeat the above procedure. Eventually, take the average of the information measurements within one period to finalize the results. This alternative method is more rigorous in terms of applying the Gaussian framework of the information measurements. However, it requires a very long time series to guarantee the sampling size (the number of period) is sufficient. It also requires knowing the perfect information of the period, which may not be realistic in practice if the period contains randomness.

In Figure 16, the minimum of the model error appears around the perfect value. The small bias in the optimal value compared with the truth comes from applying the Gaussian framework of the information measurements. When the discrepancy between $\omega_{0}^{M}$ and the truth $\omega_{0}$ increases, the model error in all the three information measurements becomes large as well. Despite the similar profiles in the model error curves in the two regimes, the model error increases significantly faster in Regime II (the resonance regime). In fact, according to (98) or (99), the contribution of the time-periodic forcing to the forecast solution is given by

(103) $$\mathrm{Contribution}\;\mathrm{of}\;\mathrm{the}\;\mathrm{time}-\mathrm{periodic}\;\mathrm{forcing}=\frac{f_{1}e^{i\omega_{1}t}}{\gamma +i(-\omega_{0}+\omega_{1})}\left(1-e^{-\left(\gamma +i\omega_{1}-i\omega_{0}\right)\left(t-t_{0}\right)}\right).$$

In addition to the error appears in the phase $e^{-(\gamma +i\omega_{1}-i\omega_{0})}$ due to an imperfect $\omega_{0}^{M}$, the resonance forcing also greatly modifies the amplitude of the contribution in (103). With a resonance forcing $\omega_{0}=\omega_{1}$, the amplitude in the contribution (103) reduces to $f_{1}/\gamma$ which can be much larger than $f_{1}/\left(\gamma +i\left(-\omega_{0}+\omega_{1}\right)\right)$ if $\omega_{0}$ is quite different from $\omega_{1}$ and γ is small. Recall in (101) that γ=0.4 here. If in the imperfect model $\omega_{0}^{M}=0\neq 1=\omega_{0}$, then a large error in the amplitude of the forcing contribution using the imperfect is expected. This is shown in Panel (b) of Figure 15. It is clear that in addition to the phase shift in both the filtering and prediction estimates, the amplitudes of these estimates are severely underestimated as well. Notably, the relative entropy here unambiguously indicates such underestimations of the amplitudes and extreme events, which cannot be captured by the RMS error and the pattern correlation.

In order to reduce the model error in the imperfect model, a typical strategy is to optimize the noise coefficient $\sigma^{M}$[1,13,15,101,140]. In Figure 17, we show the model error in the perfect model as a function of $\sigma^{M}$, where $\omega_{0}^{M}=0$. Comparing to the non-optimized values $\sigma^{M}=\sigma =2$ as indicated by the blue `x’, the model error with the optimal value $\sigma^{M}=7$ for the filter estimate (which is also nearly the optimal value for the prediction estimate) has a significant decrease. This noise inflation strategy is in fact consistent with that in dealing with many operational models or complex dynamical systems [18,141,142]. Figure 17 also confirms that noise inflation leads to a much smaller model error than the underdispersion [15,18,100,140]. In Panel (c) of Figure 15, the filter and prediction estimates with this optimized noise are shown. The amplitudes of the true signal are recaptured by the imperfect model estimates. Interestingly, the filter estimates are now almost perfectly in phase with the true signal and even the discrepancy between prediction estimates and the truth is greatly decreased. See Panel (b) for a comparison. In fact, we note that the Kalman gain has increased from $K_{\infty }=0.27$ to $K_{\infty }=0.73$, which implies that the observations now play a more important role in obtaining the filter estimates. This is the underlying reason that the filtering becomes more skillful, which also increases the skill of the prediction since the filter estimate now provides a much more accurate initial value of the prediction.

### 3.5. State Estimation and Prediction for Multiscale Slow-Fast Systems

Multiscale slow-fast systems are commonly seen in many geophysical and engineering turbulent flows [18,143,144,145,146]. A concrete example involves the coupling of random incompressible geostrophically balanced (GB) flows and random rotating compressible gravity waves in the middle latitude atmosphere [8]. Under the situation with a small Rossby number, the coupled system becomes a multiscale slow-fast system where the GB component dominates the slow-varying geophysical flows [8,147,148,149].

#### 3.5.1. A 3×3 Linear Coupled Multiscale Slow-Fast System

Here, we start with a simple 3×3 linear coupled multiscale slow-fast system,

(104) $$\begin{array}{cc} \frac{du_{1}}{dt} & =-d_{u_{1}}u_{1}+L_{12}u_{2}+L_{13}u_{3}+F_{1}\left(t\right)+\sigma_{1}{\dot{W}}_{1},\\ \frac{du_{2}}{dt} & =L_{21}u_{1}-d_{u_{2}}u_{2}+\frac{L_{23}}{\epsilon }u_{3}+F_{2}\left(t\right)+\sigma_{2}{\dot{W}}_{2},\\ \frac{du_{3}}{dt} & =L_{31}u_{1}+\frac{L_{32}}{\epsilon }u_{2}-d_{u_{3}}u_{3}+F_{3}\left(t\right)+\sigma_{3}{\dot{W}}_{3}. \end{array}$$

In (104), we assume the linear coefficients $L_{12}=-L_{21},L_{13}=-L_{31}$ and $L_{23}=-L_{32}$ such that the $L_{ij}$ forms a skew-symmetric matrix. The three damping coefficients $-d_{u_{1}},-d_{u_{2}},-d_{u_{3}}<0$ to guarantee the mean stability. $F_{1}\left(t\right),F_{2}\left(t\right)$ and $F_{3}\left(t\right)$ are external forcing that can depend on time *t*. Here, ϵ is a controllable parameter. With ϵ≪1, the coupled system has a fast oscillation structure in $u_{2}$ and $u_{3}$ while $u_{1}$ remains as a slow variable. All the variables here are real.

The coupled system in (104) can be regarded as one Fourier mode of the shallow water equations, where $u_{1}$ mimics the large-scale GB flow while $u_{2}$ and $u_{3}$ represent the analogies of the real and imaginary parts of the gravity waves. Note that the gravity waves appear in pairs and therefore the linear combinations of $u_{2}$ and $u_{3}$ in the complex plane are good surrogates of the two components of the gravity waves associated with one Fourier mode in the shallow water equation. These three variables are coupled in a linear way in (104).

Below, we study the filtering/prediction skill. The following parameters are taken:

(105) $$\begin{array}{c} d_{u_{1}}=d_{u_{2}}=d_{u_{3}}=1,\;\sigma_{1}=\sigma_{2}=\sigma_{3}=1,\;L_{12}=L_{13}=1,\;L_{21}=L_{31}=-1,\\ \;\;L_{23}=1,\;L_{32}=-1,\;F_{1}=2\cos\left(0.5t\right),\;F_{2}=F_{3}=0. \end{array}$$

Here, we only impose the deterministic time-periodic forcing to $u_{1}$. This is because we denote $u_{1}$ as the slow (or large) scale variable, which is typically driven by external forcing, such as the seasonal cycle or annual cycle [15]. On the other hand, the other two variables mostly occur in a faster time scale and the forcing is basically stochastic.

To understand the filtering/prediction skill, the following four setups are adopted:

1. *Full observations, full forecast model (F/F).* The observational operator *g* is an identity such that
  (106) $$\left(\begin{array}{c} v_{1}\\ v_{2}\\ v_{3} \end{array}\right)=\left(\begin{array}{ccc} 1 & & \\ & 1 & \\ & & 1 \end{array}\right)\left(\begin{array}{c} u_{1}\\ u_{2}\\ u_{3} \end{array}\right)+\left(\begin{array}{c} \sigma_{1}^{o}\\ \sigma_{2}^{o}\\ \sigma_{3}^{o} \end{array}\right).$$
  The forecast model is the same as in (105). Although this straightforward setup may not be practical (see below) and can be expensive when a much larger dimension of the system is considered (see next subsection), the results from such a setup can be used as a baseline for testing various modifications and reduced models as will be presented below.
2. *Partial observations, full forecast model (P/F).* The real observations typically involve the superposition of different wave components. It is usually impossible to artificially separate these components from the noisy observations. Therefore, here we let the observational operator be g=(1,1,1), namely the observation is the combination of the three variables,
  (107) $$v=\left(\begin{array}{ccc} 1 & 1 & 1 \end{array}\right)\left(\begin{array}{c} u_{1}\\ u_{2}\\ u_{3} \end{array}\right)+\sigma^{o}.$$
  The forecast model remains the same as that in (105).
3. *Partial observations, reduced forecast model (P/R).* In practice, only part of the state variables are of particular interest in filtering and prediction. These state variables usually lie in large or resolved scales, such as the GB flow. Therefore, simple reduced forecast models are typically designed to reduce the computational cost and retain the key features in filtering and predicting these variables. To this end, the following reduced forecast model is used
  (108) $$\frac{du_{1}^{M}}{dt}=-d_{u_{1}}u_{1}^{M}+F_{1}\left(t\right)+\sigma_{1}{\dot{W}}_{1},$$
  and the observation remains the same as that in (107). Here, we have completely dropped the dependence of $u_{1}$ on $u_{2}$ and $u_{3}$ since their mean is zero according to the setup above.
4. *Partial observations, reduced forecast model and tuned observational noise level with inflation (P/R tuned)*. It is easy to notice that in the previous setup (P/R), the signals of $u_{2}$ and $u_{3}$ actually become part of the observational noise in filtering and predicting $u_{1}$. This is known as the representation error [53,100,150,151,152,153,154]. However, if the original observational noise level $r^{o}$ is still used in updating the Kalman gain, then the filtering and prediction skill may be affected by the representation error. To resolve this issue, we utilize an inflated $r_{M}^{o}$ in the analysis step to compute the Kalman gain while the other setups remain the same as in the P/R case. Here, the inflated $r_{M}^{o}$ is given by
  (109) $$r_{M}^{o}=r^{o}+\mathrm{var}\left(u_{2}\right)+\mathrm{var}\left(u_{3}\right),$$
  where $var(u_{2})$ and $var(u_{3})$ are the variance of $u_{2}$ and $u_{3}$ respectively at the statistical steady state. The inflation in (109) is the most straightforward one. More elaborate inflation techniques can be reached by applying the information theory in the training phase. Nevertheless, with such a simple inflation of the observational noise, the signals of $u_{2}$ and $u_{3}$ are treated as part of the observational noise. The estimation of the Kalman gain using the imperfect forecast model (108) is therefore expected to be improved.

Below, we consider two dynamical regimes with ϵ=0.1 and ϵ=1, respectively. The two variables $u_{2}$ and $u_{3}$ evolve in a much faster time scale than $u_{1}$ in the regime with ϵ=0.1 while the three variables lie in the same time scale with ϵ=1.

**Figure 18:**
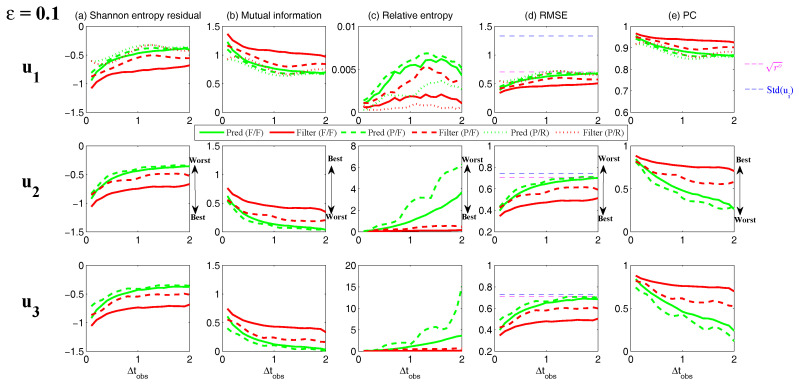
Regime ϵ=0.1. Prediction and filtering skill as a function of the observational time step $\Delta t_{obs}$ using the three information measures: (**a**) Shannon entropy of residual, (**b**) mutual information and (**c**) relative entropy as well as the two traditional path-wise measures (**d**) root-mean-square error (RMSE) and (**e**) pattern correlation (PC). The green curves are for prediction and the red curves are for filtering. The solid curves correspond to the situation with full observations and full forecast model (F/F); the dashed curves correspond to the situation with partial observations and full forecast model (P/F); and the dotted curves are for that with partial observations and reduced forecast model (P/R). The three rows are shown for the skill of $u_{1}$, $u_{2}$ and $u_{3}$, respectively. The numerical simulation is based on time series with total length $T_{total}=5000$ while the largest observational time step here is $\Delta t_{obs}=2$.

**Figure 19:**
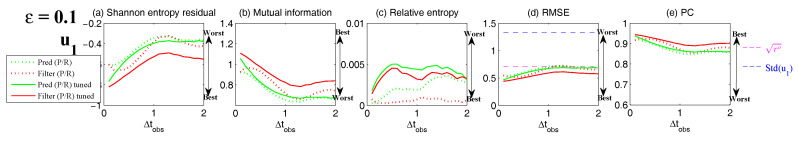
Regime ϵ=0.1. Similar to Figure 18 but the comparison of the skill of filtering and predicting $u_{1}$ based on the setup with partial observations and reduced forecast model (P/R) (dotted line) and that with partial observations, reduced forecast model and tuned observational noise level with inflation (P/R tuned) (thin solid line).

**Figure 20:**
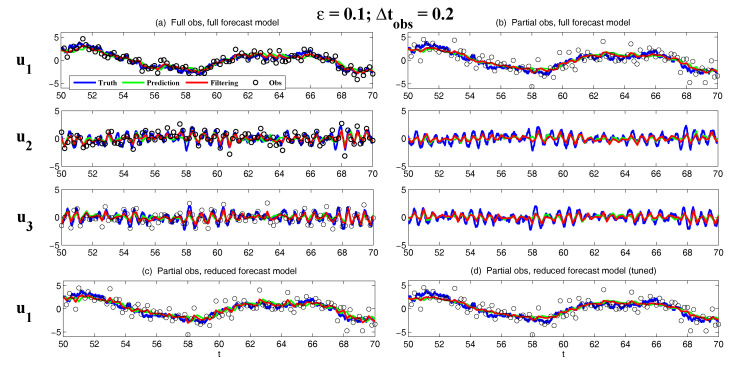
Regime ϵ=0.1 and $\Delta t_{obs}=0.2$. Comparison of the filtering and prediction skill in different setups. (**a**): full observations and full forecast model (F/F); (**b**): partial observations and full forecast model (P/F); (**c**): partial observations and reduced forecast model (P/R); and (**d**): partial observations, reduced forecast model and tuned observational noise level (P/R tuned).

**Figure 21:**
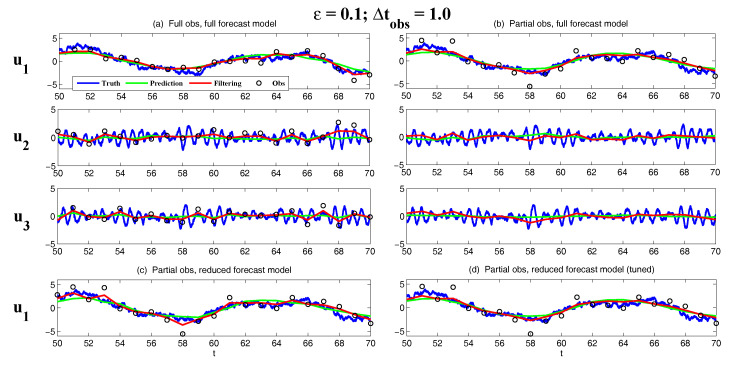
Similar to in Figure 20 but for Regime ϵ=0.1 and $\Delta t_{obs}=1.0$.

Now, we compare the filtering and prediction skill using the four setups as discussed above. In Figure 18 and Figure 19, the skill as a function of the observational time step $\Delta t_{obs}$ is shown in Regime ϵ=0.1. The following conclusions are reached. First, both the filtering and prediction skill overall deteriorates with the increase of the observational time step $\Delta t_{obs}$. Second, the filter estimates are almost always more accurate than the prediction estimates since the former contains extra information from observations. Third, the results with F/F is the best among all the four setups, as expected. Nevertheless, the filtering and prediction results of $u_{1}$ based on the other three setups remain comparable to that of F/F. However, the predictions of $u_{2}$ and $u_{3}$ using both the full and partial observations (F/F and P/F) contain a large error when the observational time step becomes large. Such an error is not reflected by the RMSE and PC but is clearly indicated by the relative entropy. In fact, since $u_{2}$ and $u_{3}$ both lie in faster time scales, their decorrelation times become much shorter than the observational time step when the latter increases. The consequence is that, regardless of the initial value, the prediction estimates always relax to the equilibrium mean and the amplitudes are thus severely weakened. Despite the success in capturing the pattern correlation, the prediction fails to catch any extreme events. On the other hand, the observations help the state estimation of filtering. In fact, the filter estimates with full observations (F/F) can almost perfectly capture the amplitudes of the truth while the partial observations (P/F) at least allow the filter estimates to reach some of the events with large amplitudes, which is nevertheless more skillful than the prediction. See Figure 20 and Figure 21 for the true time series as well as the prediction and filtering estimates.

**Figure 22:**
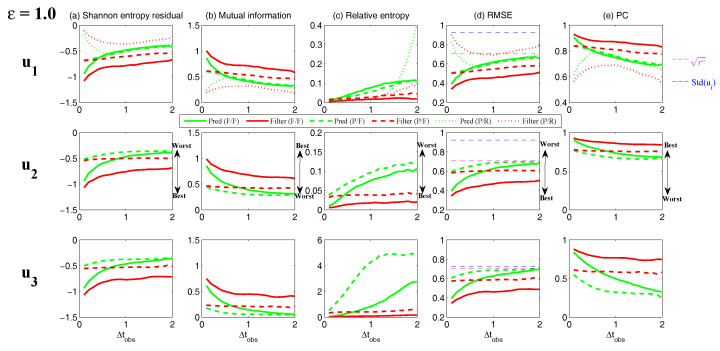
Similar to Figure 18 but for Regime ϵ=1.

**Figure 23:**
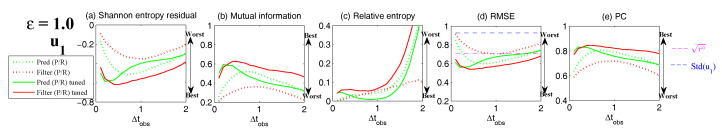
Similar to Figure 19 but for Regime ϵ=1.

**Figure 24:**
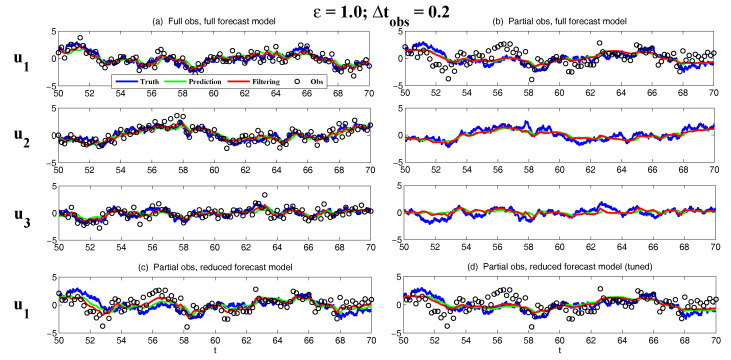
Similar to in Figure 20 but for Regime ϵ=1.0 and $\Delta t_{obs}=0.2$.

**Figure 25:**
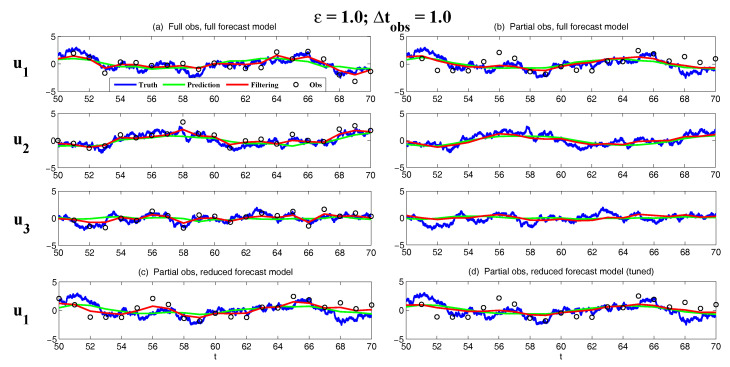
Similar to in Figure 20 but for Regime ϵ=1.0 and $\Delta t_{obs}=1.0$.

Next, in Figure 22 and Figure 23, the filtering and prediction skill in Regime ϵ=1 is shown. Now, the difference in the results between using different setups becomes more significant. The filtering and prediction skill of $u_{1}$ using P/F remains good but the gap compared with that using F/F is more obvious. Interestingly, the reduced strategy P/R now becomes much worse and the filtering results are even worse than the predictions especially with short observational time step $\Delta t_{obs}$ (see also the time series in Figure 24 and Figure 25). In fact, there are two sources of error that bring about such unskillful results. First, the variances of $u_{2}$ and $u_{3}$ with ϵ=1 are now much larger, which leads to a large representation error. Such representation error leads to a larger error in the filtering than prediction (For comparison, see Section 3.2 for the conclusion with no representation error). Second, recall that in Regime ϵ=0.1, $u_{2}$ and $u_{3}$ evolve in a much faster time scale and therefore they can be treated as noise. Ignoring them in the dynamics of $u_{1}$ provides a good approximation in (108). This is however not true in Regime ϵ=1 where the long memory of $u_{2}$ and $u_{3}$ plays an important role in the reduced dynamics of $u_{1}$. In other words, the reduced forecast model in (108) results in a large model error in Regime ϵ=1 due to the ignorance of $u_{2}$ and $u_{3}$. Thus, the combined effect from both the representation error and the imperfect model leads to the large error in filtering as well as prediction. With a short observational time step (for example, $\Delta t_{obs}=0.2$ in Figure 24), the representation error becomes dominant. Therefore, an inflation of the observational noise to compensate the representation error (P/R tuned in Figure 23) improves the filtering and prediction skill. However, with a much longer observational time step, say $\Delta t_{obs}=2$, an inflation of the observational noise (P/R tuned) will reduce the Kalman gain and the model rather than the observation provide more information to the filter results. When $\Delta t_{obs}$ is large, the model will relax towards its equilibrium mean and thus the amplitude will be underestimated. See Figure 23. This again indicates the importance of using the relative entropy as one of the quantification criteria. Note that, with $\Delta t_{obs}=2$, Figure 23 clearly states that both the RMSE and PC of the filtering estimates in P/R tuned setup are better than those in P/R setup, but the relative entropy in P/R tuned setup is much larger. This is a good example to show the importance and necessity of using the information-theoretic framework in quantifying the filtering and prediction skill instead of using only the path-wise RMSE and PC. Finally, we note that the signals of $u_{2}$ and $u_{3}$ here do not behave as a pair of oscillator as in the Regime with ϵ=0.1. This is because all the three variables now lie in the same time scale and they interact with each other. Here, the large-scale time-periodic forcing in $u_{1}$ leads to a time-periodic pattern in $u_{2}$ as well. However, the strong anti-correlation in $u_{1}$ and $u_{2}$ provides a cancelation in the feedback to $u_{3}$, which makes the signal of $u_{3}$ more noisy than $u_{2}$. The consequence is that the filtering and prediction skill in $u_{3}$ is much worse than those in $u_{2}$ due to the much larger noise to signal ratio in $u_{3}$.

To summarize, in the Regime with ϵ=0.1, all four of the setups lead to comparable results for both filtering and predicting the slow variable $u_{1}$. In particular, the most efficient strategy P/R works quite well. The filtering and prediction of the two fast variables $u_{2}$ and $u_{3}$ using F/F and P/F also show skillful results when the observational time step $\Delta t_{obs}$ is short. When $\Delta t_{obs}$ exceeds the decorrelation time of $u_{2}$ and $u_{3}$, the filter estimates tend to miss some large events while the prediction results fail to capture all the extreme events. In the Regime with ϵ=1, the reduced strategy (P/R) for $u_{1}$ does not work well especially with small observational time step $\Delta t_{obs}$. Nevertheless, if an observational noise inflation is adopted (P/R tuned), then both the filtering and prediction skill can be improved and becomes nearly comparable to those to the full filter with full observations (F/F) when $\Delta t_{obs}$ is small to moderate. When the $\Delta t_{obs}$ is large, the model error in the reduced forecast model (108) becomes dominant. In such a situation, only a full forecast model provides skillful prediction and filtering results while a partial observation (P/F) is allowed for retaining the skill. The partial observation (P/F) also gives a comparable skill as the full observation (F/F) in filtering and predicting $u_{2}$ but only in the setup with both the full forecast model and the full observations (F/F) leads to skillful results for $u_{3}$. A summary is shown in Table 1.

#### 3.5.2. Shallow Water Flows

Finally, let us study the filtering and prediction for spatially-extended systems. Consider the linearized two-dimensional rotating shallow water equation [8,143]

(110) $$\begin{array}{cc} \;\;\frac{\partial u}{\partial t}+\epsilon^{-1}u^{⊥} & =-\epsilon^{-1}\nabla \eta ,\\ \;\;\frac{\partial \eta }{\partial t}+\epsilon^{-1}\nabla \cdot u & =0, \end{array}$$

where $u={\left(u,v\right)}^{T}$ is the two-dimensional velocity field and η is the geophysical height. Here, ϵ is the Rossby number representing the ratio between the Coriolis term and the advection term. We also set the Froude number equal to the Rossby number, which is the typical case in realistic geophysical flows [8]. Applying the Fourier decomposition method (See Section 4.4 in [8]) to (110), a 3×3 system is obtained for each Fourier wavenumber. In particular, associated with each Fourier wavenumber, there are:

1. One geostrophically balanced (GB) mode with eigenvalue
  (111) $$\omega_{k,B}=0.$$
  The GB mode is incompressible.
2. Two gravity modes with eigenvalues
  (112) $$\omega_{k,\pm }=\pm \epsilon^{-1}\sqrt{{\left|k\right|}^{2}+1}.$$
  The gravity modes are compressible.

Therefore, the solution of the shallow water equation in (110) can be written as a superposition of different Fourier modes,

(113) $$\left[\begin{array}{c} u(x,t)\\ \eta (x,t) \end{array}\right]=\sum_{k\in K,\alpha \in \{B,\pm \}}{\hat{u}}_{k,\alpha }\left(t\right)\exp\left(ik\cdot x\right)r_{k,\alpha },$$

where the eigenvectors associated with the GB and gravity modes, i.e., $r_{k,0}$ and $r_{k,\pm }$, are given by

(114) $$r_{k,B}=\frac{1}{\sqrt{{\left|k\right|}^{2}+1}}\left(\begin{array}{c} -ik_{2}\\ ik_{1}\\ 1 \end{array}\right),\;r_{k,\pm }=\frac{1}{{\left|k\right|}^{2}\sqrt{{2|k|}^{2}+2}}\left(\begin{array}{c} ik_{2}\pm k_{1}\sqrt{{\left|k\right|}^{2}+1}\\ -ik_{1}\pm k_{2}\sqrt{{\left|k\right|}^{2}+1}\\ {\left|k\right|}^{2} \end{array}\right),$$

respectively, for |k|≠0 and

(115) $$r_{k,B}=\left(\begin{array}{c} 0\\ 0\\ 1 \end{array}\right),\;r_{k,\pm }=\frac{1}{\sqrt{2}}\left(\begin{array}{c} \pm i\\ 1\\ 0 \end{array}\right),$$

respectively, for |k|=0. Here, in (111)–(115), $k=(k_{1},k_{2})$ and x=(x,y).

The time evolution of the random Fourier amplitudes ${\hat{u}}_{k,\alpha }\left(t\right)$ associated with each Fourier wavenumber k can be described by the 3×3 system as introduced in (104)

(116) $$\begin{array}{cc} \frac{d{\hat{u}}_{k,1}}{dt} & =-d_{{\hat{u}}_{k,1}}{\hat{u}}_{k,1}+L_{k,12}{\hat{u}}_{k,2}+L_{k,13}{\hat{u}}_{k,3}+F_{k,1}\left(t\right)+\sigma_{k,1}{\dot{W}}_{k,1},\\ \frac{d{\hat{u}}_{k,2}}{dt} & =L_{k,21}{\hat{u}}_{k,1}-d_{{\hat{u}}_{k,2}}{\hat{u}}_{k,2}+\frac{L_{k,23}}{\epsilon }{\hat{u}}_{k,3}+\sigma_{k,2}{\dot{W}}_{k,2},\\ \frac{d{\hat{u}}_{k,3}}{dt} & =L_{k,31}{\hat{u}}_{k,1}+\frac{L_{k,32}}{\epsilon }{\hat{u}}_{k,2}-d_{{\hat{u}}_{k,3}}{\hat{u}}_{k,3}+\sigma_{k,3}{\dot{W}}_{k,3}. \end{array}$$

Note that the variables ${\hat{u}}_{k,1},{\hat{u}}_{k,2}$ and ${\hat{u}}_{k,3}$ are all real variables while the gravity modes are a pair of complex conjugate. Nevertheless, we can make use of a combination of ${\hat{u}}_{k,2}$ and ${\hat{u}}_{k,3}$ to form the two gravity waves:

(117) $$\begin{array}{cc} {\hat{u}}_{k,+} & ={\hat{u}}_{k,2}+i{\hat{u}}_{k,3},\\ {\hat{u}}_{k,-} & ={\hat{u}}_{k,3}+i{\hat{u}}_{k,2}. \end{array}$$

On the other hand, ${\hat{u}}_{k,1}={\hat{u}}_{k,B}$. Without $L_{k,12},L_{k,21},L_{k,13}$ and $L_{k,31}$, these setups are similar to those in [139,155] except that the starting 3×3 systems in [139,155] are complex and there are two extra freedoms for noise in the pair of the gravity modes. In (116), the GB and gravity modes are coupled with each other linearly through nonzero coefficients $L_{k,12},L_{k,21},L_{k,13}$ and $L_{k,31}$.

Next, the noisy observations are given by the velocity fields *u* and *v* at each grid point in physical space. This is known as the Euler observations. Note that Lagrangian observations (via Lagrangian tracers) are also widely used in filtering the shallow water flows or more generally the geophysical flows [49,139,155,156,157,158,159]. Here, the Fourier expansion is applied to the noisy observational data of *u* and *v*. We assume the observational noise is white. Therefore, the noise level associated with each Fourier wavenumber is the same [108]. Note that the observations are not the Fourier coefficients in (116). They are the summation of the three Fourier components ${\hat{u}}_{k,\alpha }$ for α={B,±} multiplying by the associated eigenvectors $r_{k,\alpha }$ in (114) and (115) according to the expression of the velocity in (113). These correspond to the setups of P/F, P/R and P/R as discussed in Section 3.5. We will also report the filtering and prediction skill using the F/F as introduced in Section 3.5, which assumes that the observation for each GB and gravity mode is available. Although such a setup is idealized, it provides the optimal filtering and prediction results and can be used to examine the skill in the other setups. Once the results are obtained for each Fourier mode associated with the 3×3 system in (116), the summation of different Fourier modes are taken to recover the velocity field in the physical space. In practice, recovering and predicting the GB flow are of particular interest since GB flows lie in a longer time scale. Therefore, we focus on the study of the GB flow in different setups (F/F, P/F, P/R and P/R tuned). Since the GB modes are incompressible, it is more convenient to show the stream function ψ instead of the velocity field, where (u,v)=(∂ψ/∂y,-∂ψ/∂x).

In the following, we consider the Fourier wavenumbers k in ${\left[-2,2\right]}^{2}$, where there are 25 GB modes and 50 gravity modes. The modes with k=(0,0) are the background modes, which are usually deterministic. Thus, we filter and predict the other 24 wavenumbers. Note that the mode k and -k are complex conjugate. The following parameters are taken in rotating shallow water Equation (110),

(118) $$\begin{array}{c} \;\;\;d_{k,u_{1}}=d_{k,u_{2}}=d_{k,u_{3}}=1,\;\sigma_{k,1}=3,\;\sigma_{k,2}=\sigma_{k,3}=2,\\ \;\;\;\;\;L_{k,12}=L_{k,13}=1,\;L_{k,21}=L_{k,31}=-1,\\ L_{k,23}=\sqrt{{\left|k\right|}^{2}+1},\;L_{k,32}=-\sqrt{{\left|k\right|}^{2}+1},\;F_{k,1}=2\cos\left(0.5t\right),\;F_{k,2}=F_{k,3}=0. \end{array}$$

Two dynamical regimes will be studied. They are ϵ=0.1 (fast rotation regime) and ϵ=1.0 (moderate rotation regime). The observational noise level is $r_{k}^{o}=1.5$. The noise to signal ratio varies in different Fourier modes, but the noise is about 30% to 40% compared with the amplitude of the true signals multiplying by the eigenvectors (which is also the observational operator here) when the mode has observability [15,140,160,161]. The observability issue will be discussed at the end of this section.

**Figure 26:**
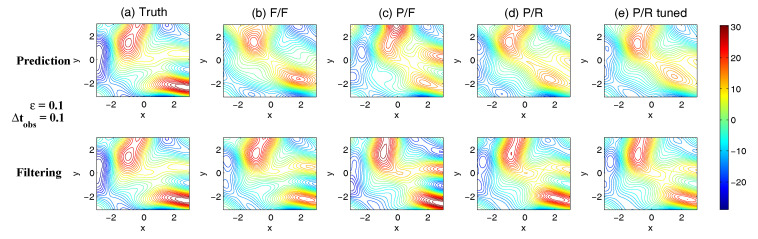
Regime ϵ=0.1 with observational time $\Delta t_{obs}=0.1$. Comparison of the prediction and filtering estimates in physical space at a fixed time t=26 using different setups: (**b**) F/F (full observations, full forecast model); (**c**) P/F (partial observations, full forecast model); (**d**) P/R (partial observations, reduced forecast model) and (**e**) P/R tuned (partial observations, reduced forecast model and tuned observational noise level with inflation). The truth is shown in column (**a**).

**Figure 27:**
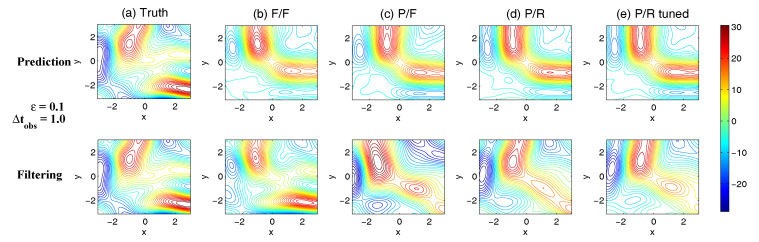
Regime ϵ=0.1 with observational time $\Delta t_{obs}=1.0$. The caption is similar to that in Figure 26.

**Figure 28:**
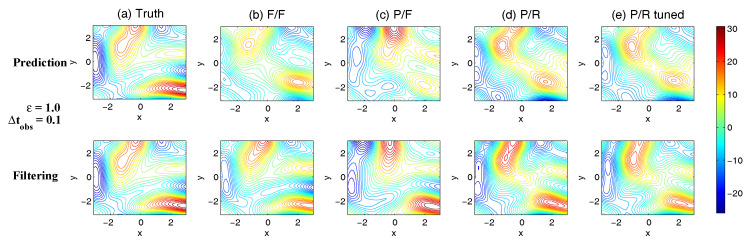
Regime ϵ=1.0 with short observational time $\Delta t_{obs}=0.1$. The caption is similar to that in Figure 26.

**Figure 29:**
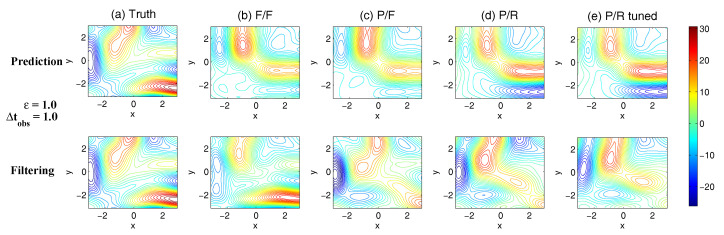
Regime ϵ=1.0 with observational time $\Delta t_{obs}=1.0$. The caption is similar to that in Figure 26.

The statistical behavior in filtering and predicting each Fourier wavenumber based on the information-theoretic framework are quite similar to those in Section 3.5.1. Therefore, in the following, we focus only on the comparison in the physical space, the results of which are given by taking the summation of different Fourier modes. In Figure 26 and Figure 27, the prediction and filtering results in Regime ϵ=0.1 are shown. With a short observational time step $\Delta t_{obs}$ (shorter than the decorrelation time of the gravity waves), both the filtering and prediction estimates are quite accurate, despite the fact that the prediction estimates in Figure 26 contain small errors in recovering the vortex in the right bottom corner. When $\Delta t_{obs}$ is increased to $\Delta t_{obs}=1$, which is longer than the memory time of the gravity waves, obvious errors are found in the predicted GB flows as shown in Figure 27. Nevertheless, the overall patterns and the amplitudes of the predicted GB flows in all the setups remain acceptable. The filtering estimates are more accurate than the predictions, especially in recovering the vortex near the left edge. On the other hand, in Regime ϵ=1, even with a short observational time step $\Delta t_{obs}=0.1$, the prediction is inaccurate. See Figure 28. The error comes from both the pattern and the amplitude, the latter of which is quantified by the relative entropy. When $\Delta t_{obs}$ becomes $\Delta t_{obs}=1$, the filtering skills using the three practical setups (P/F, P/R and P/R tuned) all contain significant errors while the prediction estimates provide completely wrong patterns such as those at the right bottom corner. See Figure 29.

**Figure 30:**
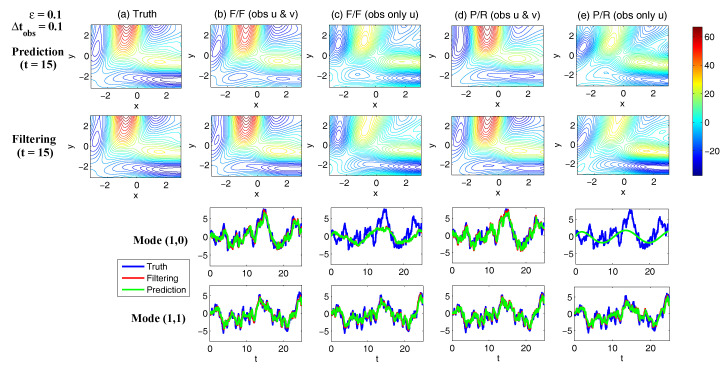
Comparison of the filtering and prediction estimates in physical space using F/F and P/R with two different types of observations. (**a**): the truth; (**b**,**d**): observing both *u* and *v*; (**c**,**e**): observing only *u*. Here, ϵ=0.1 and $\Delta t_{obs}=0.1$. The first two rows show the snapshots of the steam function at a fixed time t=15. The third and fourth rows show the time series of the two Fourier modes k=(1,0) and k=(0,1), where the filtering and prediction estimates are largely overlapped with each other. Note that the first component of vector ${\vec{r}}_{k,B}$ with k=(1,0) is zero.

One interesting question to ask is that whether the observations of both the velocity fields *u* and *v* are needed in filtering and predicting the rotation shallow water flows since these two velocity components are strongly linked through the eigenvectors (114). To answer this question, we show the filtering and prediction estimates in physical space by observing both *u* and *v* (Panels (b) and (d)) and observing only *u* (Panels (c) and (e)). See the first two rows of Figure 30. Here, the fast rotation regime ϵ=0.1 is chosen and a short observational time step $\Delta t_{obs}=0.1$ is adopted. It is clear that by observing only *u*, both the filtering and prediction estimates contain significant errors under the setups of both F/F and P/R (and others, not shown here). In fact, it is expected from Section 3.5 that with such a small $\Delta t_{obs}$ and in the small ϵ regime, both the filtering and prediction results are accurate. This is true for most of the Fourier modes, such as k=(1,1) as shown in the last row of Figure 30. However, it is seen in the third row that the estimates of mode k=(1,0) for both filtering and predictions are quite different from the truth by observing only *u*. The reason is that the first component of $r_{k,B}$ in (114) which multiplies ${\hat{u}}_{k,B}$ in obtaining the observation of *u* is zero for all modes with $k=(k_{1},0)$. This means any mode ${\hat{u}}_{k,B}$ with $k=(k_{1},0)$ has no observability. In other words, the observation *u* plays no role in the filtering process. The consequence is that both the filtering and prediction estimates of ${\hat{u}}_{k,B}$ follow exactly the mean evolution of the dynamics. This is clearly demonstrated in column (e) for P/R. On the other hand, the small fluctuations in the estimates of ${\hat{u}}_{k,B}$ in F/F are due to the coupling between ${\hat{u}}_{k,B}$ and ${\hat{u}}_{k,\pm }$ where the latter is observable. These findings indicate the importance and necessity of observing both *u* and *v*.

There are a few issues that have not been fully addressed here but can be good directions for future works. First, there might not be necessary to observe all the components of *u* and *v*. For example, observing *v* only for the modes that *u* has no observability may provide a cheaper strategy. Second, comparing the Euler and Lagrangian observations is an interesting topic. In fact, it has been shown in [49] that there exists an information barrier in recovering the velocity field using the Lagrangian observations. Whether this information barrier can be rigorously quantified by using Euler measurements and how to combine Euler and Lagrangian observations to maximize the information are both important topics that deserve further explorations. Finally, as has been noticed here, the P/R tuned setup does not significantly reduce the biases due to the representation error. Therefore, a more systematical study of understanding and improving the representation error is a good future direction.

## 4. Information, Sensitivity and Linear Statistical Response—Fluctuation–Dissipation Theorem (FDT)

In Section 2.3 and Section 2.4, we have shown the response in the statistical mean as a function of the external forcing perturbation in linear models, where analytic formulae were available and they were used to explicitly illustrate the response. For complex nonlinear dynamical systems, computing the system response due to different types of external perturbations is an important issue in many areas including climate change in climate science and feedback control in engineering. These external perturbations can be forcing (as in the examples shown in Section 2.3 and Section 2.4), dissipation, phase as well as all other types of perturbations. In addition, the response function of interest is not only the statistical mean but also the energy (variance) and many other nonlinear functions of the state variables. Clearly, for most of the nonlinear systems, analytic formulae for the statistical response are not available and direct numerical methods are too expensive to adopt. Therefore, it is important to develop a general strategy of efficiently computing the system response to any external perturbation in complex nonlinear dynamical systems.

The fluctuation–dissipation theorem (FDT) [38,39,40,162] is an attractive way to assess the system response by using the statistics of the present states. For example, the important practical and conceptual advantages for climate change science when a skillful FDT algorithm can be established is that the linear statistical response operator produced by FDT can be used directly for multiple climate change scenarios, multiple changes in forcing, dissipation and other parameters and inverse modelling directly [163,164] without the need of running the complex climate model in each individual case, often a computational problem of overwhelming complexity. With systematic approximations, FDT has been shown to have high skill for suitable regimes of general circulation models (GCMs), which are extremely complicated with an order of a million degrees of freedom [163,164].

### 4.1. Fluctuation–Dissipation Theorem (FDT)

#### 4.1.1. The General Framework

Here, we summarize the general framework of the FDT [40]. Consider a general nonlinear dynamical system with noise

(119) $$\frac{du}{dt}=F\left(u\right)+\sigma \left(u\right)\dot{W},$$

where $u\in R^{N}$ is the state variables, σ is an N×K noise matrix and $\dot{W}\in R^{K}$ is a *K*-dimensional white noise. The evolution of the PDF p(u) associated with u is driven by the so-called Fokker–Planck equation [108],

(120) $$\frac{\partial p}{\partial t}=-{div}_{u}\left[F\left(u\right)p\right]+\frac{1}{2}{div}_{u}\nabla_{u}\left(\Sigma p\right)\equiv L_{FP}p,$$

where $\Sigma =\sigma \sigma^{T}$ and ${p|}_{t=0}=p_{0}\left(u\right)$. Let $p_{eq}\left(u\right)$ be the smooth equilibrium PDF that satisfies $L_{FP}p_{eq}=0$. The statistics of some function A(u) are determined by

(121) $$\langle A\left(u\right)\rangle =\int A\left(u\right)p_{eq}\left(u\right)du.$$

Now, consider the dynamical in (119) by a small external forcing perturbation δF(u,t). The perturbed system reads

(122) $$\frac{du}{dt}=F\left(u\right)+\delta F\left(u,t\right)+\sigma \left(u\right)\dot{W}.$$

We further assume an explicit time-separable structure for δF(u,t), which occurs in many applications [40,97,165], namely

(123) δF(u,t)=δw(u)f(t).

Then, the Fokker–Planck equation associated with the perturbed system (122) is given by

(124) $$\begin{array}{cc} \frac{\partial p^{\delta }}{\partial t} & =L_{FP}p^{\delta }+\delta L_{ext}p^{\delta },\\ where\;\;\delta L_{ext}p^{\delta }=L_{ext}p\cdot & \delta F\left(t\right),\;L_{ext}p=-\frac{\partial }{\partial u_{i}}\left(w_{i}\left(u\right)p\right),\;1\leq i\leq N. \end{array}$$

Similar to (121), for the perturbed system (124) the expected value of the nonlinear functional A(u) is given by

(125) $${\langle A\left(u\right)\rangle }^{\delta }=\int A\left(u\right)p^{\delta }\left(u\right)du.$$

The goal here is to calculate the change in the expected value

(126) $$\delta \langle A\left(u\right)\rangle ={\langle A\left(u\right)\rangle }^{\delta }-\langle A\left(u\right)\rangle .$$

To this end, let’s take the difference between (120) and (124),

(127) $$\frac{\partial }{\partial t}\delta p=L_{FP}\delta pp+\delta L_{ext}p_{eq}+\delta L_{ext}\delta p,$$

where $\delta p=p^{\delta }-p_{eq}$ is the small perturbation in the PDF. Ignoring the higher order term $\delta L_{ext}\delta p$ assuming δ is small, (127) reduces to

(128) $$\begin{array}{cc} \frac{\partial }{\partial t}\delta p & =L_{FP}\delta pp+\delta L_{ext}p_{eq},\\ {\delta p|}_{t=0} & =0. \end{array}$$

Since $L_{FP}$ is a linear operator, with the semigroup notation, $\exp[tL_{FP}]$, for this solution operator, the solution of (128) is written concisely as

(129) $$\delta p=\int_{0}^{T}\exp\left[\left(t-t^{\prime }\right)L_{FP}\right]\left(\delta L_{ext}\left(t^{\prime }\right)p_{eq}\right)dt^{\prime }.$$

Now, combining (129) with (124) and (126), we arrive at the *linear response formula*

(130) $$\delta \langle A\left(u\right)\rangle \left(t\right)=\int_{R^{N}}A\left(u\right)\delta p\left(u,t\right)du=\int_{0}^{t}R\left(t-t^{\prime }\right)\cdot \delta F\left(t^{\prime }\right)dt^{\prime },$$

where the vector linear response operator is given by

(131) $$R\left(t\right)=\int_{R^{N}}A\left(u\right)\left(\exp\left[tL_{FP}\right]\left[L_{ext}p_{eq}\right]\right)\left(u\right)du.$$

This general calculation is the first step in the FDT. However, for nonlinear systems with many degrees of freedom, direct use of the formula in (131) is completely impractical because the exponential $\exp[tL_{FP}]$, cannot be calculated directly.

FDT states that, if δ is small enough, then the leading-order correction to the statistics in (121) becomes [40]

(132) $$\delta \langle A\left(u\right)\rangle \left(t\right)=\int_{0}^{t}R\left(t-s\right)\delta f\left(s\right)ds,$$

where R(t) is the linear response operator, which is calculated through correlation functions in the unperturbed climate:

(133) $$R\left(t\right)=\langle A\left[u\left(t\right)\right]B\left[u\left(0\right)\right]\rangle ,\;B\left(u\right)=-\frac{{div}_{u}\left(wp_{eq}\right)}{p_{eq}}.$$

See [40] for a rigorous proof of (132) and (133). Clearly, calculating the correlation functions in (133) via FDT is much cheaper and practical than directly computing the linear response operator (131).

Before we move on to the more specific FDT algorithms, let’s comment on the perturbation function in (122) and (123). In fact, if w has no dependence on u, then δF(t) naturally represents the forcing perturbation. If w(u) is a linear function of u, then δF(u,t) represents the perturbation in dissipation. It is also clear that if the functional A(u) in (132) is given by A(u)=u, then the response computed is for the statistical mean. Likewise, $A\left(u\right)={\left(u-\bar{u}\right)}^{2}$ is used for computing the response in the variance.

Notably, despite the small perturbation, FDT (132) and (133) does not require any linearization of the underlying dynamics in (119). Therefore, it captures the nonlinear features in the underlying turbulent systems.

#### 4.1.2. Approximate FDT Methods

One major issue in applying FDT directly in the form of (133) is that the equilibrium measure $p_{eq}\left(u\right)$ is not known exactly. Therefore, different approximate methods have been proposed to compute the linear response operator.

**Quasi-Gaussian (qG) FDT.** Among all the approximate methods, the quasi-Gaussian (qG) approximation is one of the most effective approaches. It uses the approximate equilibrium measure

(134) $$p_{eq}^{G}=C_{N}\exp\left[-\frac{1}{2}{\left(u-\bar{u}\right)}^{*}R^{-1}\left(u-\bar{u}\right)\right],$$

where the mean $\bar{u}$ and covariance matrix *R* match those in the equilibrium $p_{eq}$. One then calculates

(135) $$B^{G}\left(u\right)=-\frac{{\mathrm{div}}_{u}\left(wp_{eq}^{G}\right)}{p_{eq}^{G}}$$

and replaces B(u) by $B^{G}\left(u\right)$ in the qG FDT. The correlation in (133) with this approximation is calculated by integrating the original system in (119) over a long trajectory or an ensemble of trajectories covering the attractor for shorter times assuming mixing and ergodicity for (133).

For the special case of changes in external forcing $w{\left(u\right)}_{i}=e_{i}$, i≤i≤N, the response operator for the qG FDT is given by the matrix

(136) $$R^{G}\left(t\right)=\langle A\left(u\left(t\right)\right)C^{-1}\left(u-\bar{u}\right)\left(0\right)\rangle .$$

The qG FDT will be applied in the simple example in Section 4.2.

**Kicked FDT.** One strategy to approximate the linear response operator which avoids direct evaluation of $\pi_{\mathrm{eq}}$ through the FDT formula is through the *kicked response* of an unperturbed system to a perturbation δu of the initial state from the equilibrium measure [30], that is,

(137) $$\pi {|}_{t=0}=\pi_{\mathrm{eq}}\left(u-\delta u\right)=\pi_{\mathrm{eq}}-\delta u\cdot \nabla \pi_{\mathrm{eq}}+O\left(\delta^{2}\right).$$

One important advantage of adopting this kicked response strategy is that higher order statistics due to nonlinear dynamics will not be ignored (compared with other linearized strategy using only Gaussian statistics [162]). Then, the kicked response theory gives the following fact [28,40] for calculating the linear response operator:

**Fact**: For δ small enough, the linear response operator $R\left(t\right)$ can be calculated by solving the unperturbed system (119) with a perturbed initial distribution in (137). Therefore, the linear response operator can be achieved through

(138) $$R\left(t\right)=\int A\left(u\right)\delta \pi +O\left(\delta^{2}\right).$$

Here, δπ is the resulting leading order expansion of the transient density function from unperturbed dynamics using initial value perturbation. The straightforward Monte Carlo algorithm to approximate (138) is sketched elsewhere [40,50]. The use of kicked FDT in calibrating the reduced-order models will be illustrated in Section 6.3.

### 4.2. Information Barrier for Linear Reduced Models in Capturing the Response in the Second Order Statistics

In this subsection, we use a simple 2D example to systematically illustrate the procedure of the FDT as introduced above. We also aim at showing the information barrier for linear reduced models in capturing the response beyond the first-order statistics. Note that such an information barrier was first pointed out in [41] with detailed discussions and more complicated examples.

The perfect model here is the SPEKF type of non-Gaussian model as discussed in (11), except that for simplicity we adopt a constant forcing $f_{u}$ in the equation of *u*,

(139) $$\begin{array}{cc} \frac{du}{dt} & =-\gamma u+f_{u}+\sigma_{u}{\dot{W}}_{u},\\ \frac{d\gamma }{dt} & =-d_{\gamma }\left(\gamma -\hat{\gamma }\right)+\sigma_{\gamma }{\dot{W}}_{\gamma }. \end{array}$$

The following parameters are used in (139) in order to generate non-Gaussian statistics of *u*,

(140) $$\sigma_{u}=0.5,\;d_{\gamma }=1.3,\;\sigma_{\gamma }=1,\;\hat{\gamma }=1,\;f_{u}=1.$$

**Figure 31:**
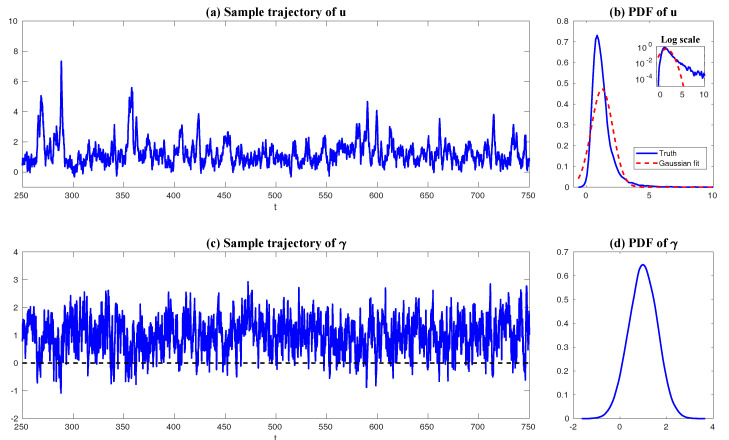
(**a**,**b**): Sample trajectories of *u* and γ in the stochastic parameterized extended Kalman filter (SPEKF) type of non-Gaussian model (139); (**c**,**d**): the corresponding PDFs. The subpanel within (**b**) shows the PDF in logarithm scale, with the red curves representing the Gaussian fit. The parameters associated with these figures are given in (140).

In Figure 31, sample trajectories and the associated PDFs of the SPEKF type non-Gaussian model (139) with parameters (140) are shown. Since γ frequently crosses zero and becomes negative, the corresponding signal of *u* is intermittent. Consequently, *u* has a skewed non-Gaussian PDF with a one-side fat tail.

**Figure 32:**
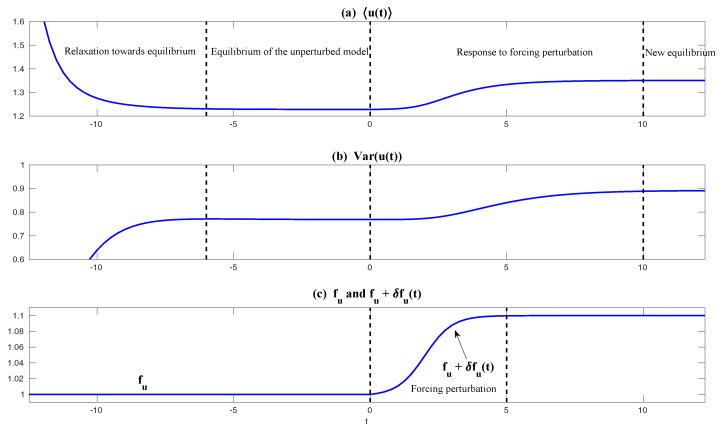
Time evolution of the mean 〈u〉 and variance Var(u(t)) of *u* (**a**,**b**) and the corresponding forcing $f_{u}\left(t\right)$ (panel (**c**)). The forcing $f_{u}\left(t\right)$ is perturbed at time t=0 with δf(t) given in (142). The mean and variance of *u* have corresponding responses and eventually arrive at a new equilibrium.

With this constant forcing $f_{u}\equiv 1$, the time evolutions of the mean 〈u〉 and variance Var(u) of *u* are shown in Figure 32. For simplicity of the discussion below, the initial time here is set to be $t_{0}=-12$. It is clear that after *t* reaches around t=-6, the model (139) arrives at the statistical equilibrium.

Now, we add a forcing perturbation $\delta f_{u}\left(t\right)$ to the model in (139),

(141) $$\begin{array}{cc} \frac{du}{dt} & =-\gamma u+f_{u}+\delta f_{u}\left(t\right)+\sigma_{u}{\dot{W}}_{u},\\ \frac{d\gamma }{dt} & =-d_{\gamma }\left(\gamma -\hat{\gamma }\right)+\sigma_{\gamma }{\dot{W}}_{\gamma }. \end{array}$$

The function $\delta f_{u}\left(t\right)$ is a ramp-type perturbation with the following form

(142) $$\delta f_{u}\left(t\right)=A_{0}\frac{\tanh\left(a\left(t-t_{c}\right)\right)+\tanh\left(at_{c}\right)}{1+\tanh(at_{c})},$$

with

(143) $$A_{0}=0.1,\;a=1,\;t_{c}=2.$$

The profile of $\delta f_{u}\left(t\right)$ is shown in panel (c) of Figure 32. The forcing perturbation $\delta f_{u}\left(t\right)$ starts from 0 at time t=0 and it reaches 0.1 at roughly t=5. After t=5, $\delta f_{u}\left(t\right)$ stays at $\delta f_{u}\left(t\right)=0.1$. Due to this forcing perturbation, the mean 〈u〉 and variance Var(u) also have corresponding changes, which are shown in panels (a) and (b) of Figure 32. Note that these responses are computed by using the analytical formulas of the time evolutions of the statistics, which are accurate. They are known as the idealized responses.

In most realistic scenarios, the true dynamics is unknown or it is too expensive to run the full perfect model. Therefore, simplified or reduced models are widely used in computing the responses. One type of the simple models that are widely adopted is the linear model,

(144) $$du^{M}=-d_{u}^{M}u^{M}+f_{u}^{M}+\sigma_{u}^{M}\dot{W}.$$

Note that adopting such a linear model to compute the responses shares the same philosophy as one of the ad-hoc-FDT procedures [166], where linear regression approximate stochastic model [87] is used for the variables of interest before applying FDT.

The three parameters in (144) are calibrated by matching the equilibrium mean, equilibrium variance and decorrelation time with those of *u* in the perfect model (139), where

(145) $${\langle u\rangle }_{eq}=\frac{f_{u}^{M}}{d_{u}^{M}},\;Var{\left(u\right)}_{eq}=\frac{{\left(\sigma_{u}^{M}\right)}^{2}}{2d_{u}^{M}},\;\tau_{corr}=\frac{1}{d_{u}^{M}}.$$

Note that the autocorrelation function of *u* in (139) with parameters in (140) does not have a strong oscillation decaying structure, and therefore matching the decorrelation is sufficient for the calibration purpose here. With such calibrations, the linear model (144) automatically fit the unperturbed mean and variance at t=0. Now, we add the same forcing perturbation to the linear model,

(146) $$du^{M}=-d_{u}^{M}u^{M}+f_{u}^{M}+\delta f_{u}\left(t\right)+\sigma_{u}^{M}\dot{W}.$$

**Figure 33:**
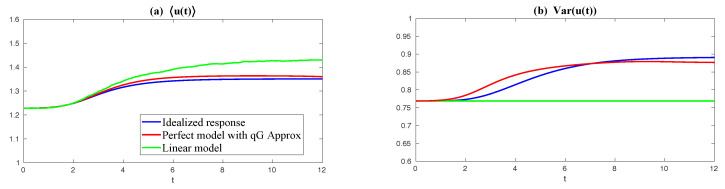
Responses to the mean 〈u〉 (panel (**a**)) and variance Var(u(t)) (panel (**b**)) of *u* with the forcing perturbation δf(t) given in (142). The perturbation starts at time t=0, which is consistent with that in Figure 32.

Since the statistics in the linear model is Gaussian, the formulas in (134)–(136) become rigorous with no approximation. In computing the responses to the forcing perturbation $\delta f_{u}\left(t\right)$ in the mean and variance of *u*, the functional A(u(t)) is set to be

(147) $$\begin{array}{cc} \mathrm{Response}\;\mathrm{in}\;\mathrm{the}\;\mathrm{mean}: & \;A\left(u\left(t\right)\right)=u^{M},\\ \mathrm{Response}\;\mathrm{in}\;\mathrm{the}\;\mathrm{variance}: & \;A\left(u\left(t\right)\right)={\left(u^{M}-{\bar{u}}^{M}\right)}^{2}, \end{array}$$

respectively. These responses using such a linear model are shown in Figure 33 (green colors) while the idealized responses are shown in blue for reference. It is clear that the response in the mean using the linear model captures the trend of the truth, but the amplitude is severely overestimated. On the other hand, the response in the variance using the linear model is identically zero and therefore it completely misses the truth. In fact, inserting the second Equation (147) into (136) yields solving a third-order centered moment. However, all odd-centered moments automatically vanish for Gaussian distribution and therefore the response in the variance using the linear model is zero [41], which has already been mentioned in Section 2.2.1 and Section 2.3. These results unambiguously indicate the insufficiency of using linear approximate models as well as the ad hoc-FDT [166] to compute the responses when the underlying dynamics is highly nonlinear.

As a comparison, we also show the responses using the qG FDT based on the perfect model (139). Since the forcing perturbation is only on the direction of *u*, w(u) in (135) is given by $w\left(u\right)={\left[1,0\right]}^{T}$. In Figure 33, it is clear that the qG FDT based on the perfect model (red) captures the response in the mean quite accurately. In addition, this qG FDT also results in a response in the variance and the skill in recovering the time evolution of the variance response is pretty good. Notably, although the response operator R(t) in (132) is linear and the Gaussian approximation (134) is used in computing the equilibrium PDF of the unperturbed system, the underlying nonlinear dynamics is used in computing the functional A(u(t)) in (147). Therefore, the nonlinear interaction is included in the FDT and the response in the variance is captured to a large extent. It is of importance to keep in mind that FDT does not implement linearization on the original underlying nonlinear system. Thus, the nonlinear dynamical features are reflected in the FDT. The linearization is applied only in the response operator due to small perturbations.

Although the simple test example here deals with a constant forcing in the unperturbed system, the FDT technique can be easily generalized to the systems with time-periodic settings, which usually corresponds to annual or seasonal cycles in climate, atmosphere and ocean sciences. Mathematical theories of the generalizations of FDT to time-dependent ensembles can be found in [162]. In [167], a triad nonlinear stochastic model with time-periodic setting was developed, which mimics the nonlinear interaction of two Rossby waves forced by baroclinic processes with a zonal jet forced by a polar temperature gradient. Systematical studies showed that qG FDT has surprisingly high skill for the mean response to the changes in forcing. The performance of qG FDT for the variance response to the perturbations of dissipation is good in the nearly Gaussian regime and deteriorates in the strongly non-Gaussian regime. More examples can be found in [15,40].

Other FDT techniques that have skillful performance in dealing with complex nonlinear dynamical systems includes blended response algorithms [168,169] and kicked FDT [30]. FDT has been demonstrated to have high skill for the mean and variance response in the upper troposphere for changes in tropical heating in a prototype atmospheric GCM and can be utilized for complex multiple forcing and inverse modeling issues of interest in climate change science [163,164]. Note that GCMs usually have a huge number of state variables and applying FDT on the entire phase space is impractical due to the limitations in calculating the covariance matrix. Practical strategies involve computing the response operator on a reduced subspace. Mathematical principles of applying FDT on reduced subspaces can be found in [41].

### 4.3. Information Theory for Finding the Most Sensitive Change Directions

An important question in climate change is how to find the most sensitive directions for climate change given the present climate. To quantify these most sensitive directions, consider a family of parameters $\lambda \in R^{p}$ with $\pi_{\lambda }$ the PDF of the true climate as a function of λ. Here λ=0 corresponds to the unperturbed state or the present climate π. Note that λ can consist of external parameters such as changes in forcing or parameters of internal variability such as a change in dissipation. In light of the information theoretic framework, the most sensitive perturbed climate is the one with the largest uncertainty related to the unperturbed one,

(148) $$P\left(\pi_{\lambda_{*}},\pi \right)=\max_{\lambda \in R^{p}}P\left(\pi_{\lambda },\pi \right).$$

The calculation of the most sensitive perturbation for the present climate in (148) is through the information theoretical framework. Assume that $\pi_{\lambda }$ is differentiable with respect to the parameter λ [90,162,170]. Since $\pi_{\lambda }{|}_{\lambda =0}=\pi$, for small values of λ, we have

(149) $$P\left(\pi_{\lambda },\pi \right)=\lambda \cdot I\left(\pi \right)\lambda +{O(|\lambda |}^{3}),$$

where λ·I(π)λ is the quadratic form in λ given by the Fisher information [40,93,162,171]

(150) $$\lambda \cdot I\left(\pi \right)\lambda =\int \frac{{\left(\lambda \cdot \nabla_{\lambda }\pi \right)}^{2}}{\pi },$$

and the elements of the matrix of this quadratic form are given by

(151) $$I_{kj}\left(\pi \right)=\int \frac{\frac{\partial \pi }{\partial \lambda_{k}}\frac{\partial \pi }{\partial \lambda_{j}}}{\pi }.$$

Detailed derivations of (149)–(151) are included in Appendix A. Note that the gradients are calculated at the unperturbed state λ=0. Therefore, if both the unperturbed state π and the gradients $\lambda \cdot \nabla_{\lambda }\pi$ are known, then the most sensitive perturbation direction occurs along the unit direction $e_{\pi }^{*}\in R^{p}$ which is associated with the largest eigenvalue $\lambda_{\pi }^{*}$ of the quadratic form in (150).

Below, we use two simple examples to provide insights of the above information theoretical framework in finding the most sensitive change direction in the underlying models. We will start with a linear example, where all the results using the direct calculation method can be written down explicitly. We aim at comparing the results using the direct method and using the Fisher information in (150). The analytic formulae associated with this linear example also allow us to understand the contributions of the uncertainty in the perturbation from the signal and the dispersion parts, respectively. Then, we will use a more complicated nonlinear example with non-Gaussian noise to show the efficiency and accuracy of using the information criterion in (150).

The first example is an one-dimensional linear model,

(152) $$\frac{du}{dt}=-au+f+\sigma \dot{W},$$

the equilibrium PDF of which is Gaussian and is given by $N(\bar{u},C)$,

(153) $$\pi \left(u\right)=N_{C}\exp\left(-\frac{{\left(u-\bar{u}\right)}^{2}}{2C}\right),$$

with

(154) $$\bar{u}=\frac{f}{a},\;C=\frac{\sigma^{2}}{2a}.$$

The two-dimensional parameters $\lambda ={\left(f,a\right)}^{T}\in R^{2}$ for external forcing and dissipation are the natural parameters which are varied in this model. Therefore, the corresponding I(λ) in (150) is a 2×2 matrix with entries $I_{ij},i,j=1,2$. Using (153), it is straightforward to compute the first-order derivatives of π with respect to *f* and *a*,

(155) $$\begin{array}{cc} \frac{\partial \pi }{\partial f} & =\frac{u-\bar{u}}{aC}\pi ,\\ \frac{\partial \pi }{\partial a} & =\frac{\sigma^{2}}{4a^{2}C}\pi -\frac{f(u-\bar{u})}{a^{2}C}\pi -\frac{\sigma^{2}{\left(u-\bar{u}\right)}^{2}}{4a^{2}C^{2}}\pi . \end{array}$$

In light of (151) and (155), the four elements of *I* have the following explicit expressions:

(156) $$\begin{array}{cc} I_{11} & =\int \frac{{\left(\frac{\partial \pi }{\partial f}\right)}^{2}}{\pi }du=\int \frac{{\left(u-\bar{u}\right)}^{2}}{C^{2}}\frac{\pi }{a^{2}}du=\frac{1}{Ca^{2}},\\ I_{12}=I_{21} & =\int \frac{\frac{\partial \pi }{\partial f}\frac{\partial \pi }{\partial a}}{\pi }du=\int \frac{u-\bar{u}}{aC}\left(\frac{\sigma^{2}}{4a^{2}C}-\frac{f(u-\bar{u})}{a^{2}C}-\frac{\sigma^{2}{\left(u-\bar{u}\right)}^{2}}{4a^{2}C^{2}}\right)\pi du=-\frac{f}{a^{3}C},\\ I_{22} & =\int \frac{{\left(\frac{\partial \pi }{\partial a}\right)}^{2}}{\pi }=\int {\left(\frac{\sigma^{2}}{4a^{2}C}-\frac{f(u-\bar{u})}{a^{2}C}\pi -\frac{\sigma^{2}{\left(u-\bar{u}\right)}^{2}}{4a^{2}C^{2}}\right)}^{2}\pi du\\ & =\int \left[{\left(\frac{\sigma^{2}}{4a^{2}C}\right)}^{2}+{\left(\frac{f(u-\bar{u})}{a^{2}C}\right)}^{2}+{\left(\frac{\sigma^{2}{\left(u-\bar{u}\right)}^{2}}{4a^{2}C^{2}}\right)}^{2}-2\frac{\sigma^{2}}{4a^{2}C}\frac{\sigma^{2}{\left(u-\bar{u}\right)}^{2}}{4a^{2}C^{2}}\right]\pi du\\ & =-\frac{f^{2}}{Ca^{4}}+\frac{\sigma^{4}}{8C^{2}a^{4}}. \end{array}$$

Now, let’s implement numerical experiments. The following two groups of parameters are used:

(157) $$\begin{array}{cc} (a): & \;a=1,\;f=1,\;\sigma =1,\\ (b): & \;a=1,\;f=1,\;\sigma =3. \end{array}$$

Since *I* is a 2×2 matrix, there are only two eigenmodes. The eigenvector w associated with the larger eigenvalue corresponds to the most sensitive direction with respect to the perturbation ${\left(\delta f,\delta a\right)}^{T}$.

By plugging the model parameters (157) into the *I* matrix in (156), we find the most sensitive direction in both of the cases:

(158) $$\left(a\right):\;e_{\pi }^{*}=\left(\begin{array}{c} -0.6618\\ 0.7497 \end{array}\right),\;\;\left(b\right):\;e_{\pi }^{*}=\left(\begin{array}{c} -0.3554\\ 0.9347 \end{array}\right).$$

To gain more intuition on the results of these most sensitive directions, we make use of the simple structure of (152) to solve this problem in an alternative way. In fact, given small perturbations ${\left(\delta f,\delta a\right)}^{T}$ to ${\left(f,a\right)}^{T}$, the corresponding perturbed mean and variance can be written down explicitly

(159) $${\bar{u}}^{\delta }=\frac{f+\delta f}{a+\delta a},\;C^{\delta }=\frac{\sigma^{2}}{2(a+\delta a)}.$$

Since both the unperturbed and perturbed PDFs are Gaussian, we can easily make use of the explicit formula of the relative entropy in (6) to compute the uncertainty due to the perturbation $P(\pi ,\pi^{\delta })$ and find the most sensitive direction in the two-dimensional parameter space. Recall in (6) that the total uncertainty can be decomposed into signal and dispersion parts. Making use of (154) and (159), we have

(160) $$\begin{array}{cc} \mathrm{Signal} & =\frac{1}{2}{\left(\frac{f}{a}-\frac{f+\delta f}{a+\delta a}\right)}^{2}{\left(\frac{\sigma^{2}}{2a}\right)}^{-1}=\frac{1}{2}\frac{{\left(fa+f\delta a-fa-a\delta f\right)}^{2}}{a^{2}{\left(a+\delta a\right)}^{2}}\frac{2a}{\sigma^{2}}\\ & =\frac{{\left(f\delta a-a\delta f\right)}^{2}}{a\sigma^{2}}+o\left(\delta a^{3}\right)+o\left(\delta a^{2}\delta f\right)+o\left(\delta a\delta f^{2}\right)\\ \mathrm{Dispersion} & =-\frac{1}{2}\ln\left(\frac{a+\delta a}{a}\right)+\frac{1}{2}\left(\frac{a+\delta a}{a}-1\right)=-\frac{1}{2}\ln\left(1+\frac{\delta a}{a}\right)+\frac{1}{2}\frac{\delta a}{a}\\ & =-\frac{1}{2}\left(\frac{\delta a}{a}-\frac{1}{2}{\left(\frac{\delta a}{a}\right)}^{2}+o{\left(\frac{\delta a}{a}\right)}^{3}\right)+\frac{1}{2}\frac{\delta a}{a}\\ & =\frac{1}{4}{\left(\frac{\delta a}{a}\right)}^{2}+o{\left(\frac{\delta a}{a}\right)}^{3}. \end{array}$$

Note that the dispersion part depends only on the perturbation in the dissipation δa since *f* has no effect on the variance. In addition, it is clear that δa and δf should have opposite signs in order to maximize the relative entropy in the signal part.

**Figure 34:**
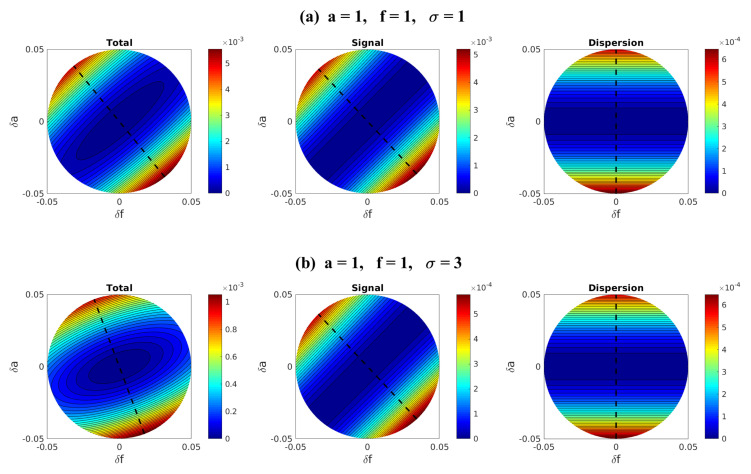
Uncertainty dependence of the perturbation in the two-dimensional parameter space ${\left(\delta a,\delta f\right)}^{T}$ in the linear model (152). (**a**): a=1,f=1,σ=1; (**b**): a=1,f=1,σ=3. The total uncertainty (**left column**) is decomposed into signal (**middle column**) and dispersion (**right column**) parts according to (6). The black dashed line shows the direction of the maximum uncertainty, namely the most sensitive direction of perturbation.

Figure 34 shows the total relative entropy as well as its two components, namely signal and dispersion, as a function of the perturbations in the two-dimensional parameter space ${\left(\delta f,\delta a\right)}^{T}$ using the direct formula (160). The numerical simulation here assumes $\sqrt{\delta f^{2}+\delta a^{2}}\leq 0.05$ to guarantee the perturbation is small enough. In both cases, the most sensible direction with respect to only the dispersion part lies in the direction ${\left(\delta a,\delta f\right)}^{T}={\left(1,0\right)}^{T}$, due to the fact that δf has no effect on the dispersion part. In the signal part, the most sensitive direction satisfies aδf=-fδa. The overall most sensitive direction depends naturally on the weights of signal and dispersion parts. When σ becomes larger, the weight on the signal part reduces since the signal part is proportional to the inverse of the model variance. It is easy to see that the most sensitive directions as indicated by the black dashed lines in Figure 34 are consistent with the theoretical prediction from (158) using the Fisher information (148)–(151).

Now, we consider a second example with a nonlinear model [116,170],

(161) $$\frac{du}{dt}=\left(f+au+bu^{2}-cu^{3}\right)+\left(A-Bu\right){\dot{W}}_{C}+\sigma {\dot{W}}_{A}.$$

The nonlinear model in (161) is a canonical model for low frequency atmospheric variability and was derived based on stochastic mode reduction strategies. This one-dimensional, normal form was applied in a regression strategy in [116] for data from a prototype AOS model [112] to build one-dimensional stochastic models for low-frequency patterns such as the North Atlantic Oscillation (NAO) and the leading principal component (PC-1) that has features of the Arctic Oscillation. Note that the model in (161) has both correlated additive and multiplicative noise $\left(A-Bu\right){\dot{W}}_{C}$ as well as an extra uncorrelated additive noise $\sigma {\dot{W}}_{A}$. The nonlinearity interacting with noise allows a rich dynamical features in the model such as strongly non-Gaussian PDFs and multiple attractors. Different from the previous example with linear dynamics, the direct method has no explicit solution for the nonlinear system (161). The goal here is to find the most sensitive directions using the information theory developed above in different dynamical regimes.

Here, we consider a simple case with A=B=0 such that the model has only additive noise. Nevertheless, the cubic nonlinearity still allows the model to have strong non-Gaussian characteristics. With A=B=0, the equilibrium PDF of (161) is given by the following explicit formula

(162) $$\pi \left(u\right)=N_{0}\exp\left(\frac{2}{\sigma^{2}}\left(fu+\frac{a}{2}u^{2}+\frac{b}{3}u^{3}-\frac{c}{4}u^{4}\right)\right).$$

We again look at the perturbation in the two-dimensional parameter space $\lambda ={\left(f,a\right)}^{T}$, which represent the changes in forcing and damping. Following (149)–(151), we aim at solving the eigenvectors of the 2×2 matrix I(λ). To explicitly write down the elements in I(λ), we define

(163) $$H_{k}=\int u^{k}\psi \left(u\right)du,\;k\geq 0\;\mathrm{with}\;\psi \left(u\right)=\exp\left(\frac{2}{\sigma^{2}}\left(fu+\frac{a}{2}u^{2}+\frac{b}{3}u^{3}-\frac{c}{4}u^{4}\right)\right).$$

Straightforward calculations show that

(164) $$\begin{array}{cc} I_{11} & =\int \frac{{\left(\frac{\partial \pi }{\partial f}\right)}^{2}}{\pi }du=\frac{4}{\sigma^{4}H_{0}^{4}}\left(H_{0}H_{2}-H_{1}^{2}\right),\\ I_{12}=I_{21} & =\int \frac{\frac{\partial \pi }{\partial f}\frac{\partial \pi }{\partial a}}{\pi }du=\frac{2}{\sigma^{4}H_{0}^{4}}\left(H_{0}H_{3}-H_{1}H_{2}\right),\\ I_{22} & =\int \frac{{\left(\frac{\partial \pi }{\partial a}\right)}^{2}}{\pi }du=\frac{1}{\sigma^{4}H_{0}^{4}}\left(H_{0}H_{2}-H_{2}^{2}\right). \end{array}$$

Now, we focus on the case studies in the following three regimes,

(165) $$\begin{array}{cccccc} \mathrm{Regime}\;I & :\;f=1.8,\; & a=0,\; & b=-5.4,\; & c=4,\; & \sigma =\sqrt{0.5},\\ \mathrm{Regime}\;\mathrm{II} & :\;f=-0.005,\; & a=-0.018,\; & b=0.006,\; & c=0.003,\; & \sigma =0.226,\\ \mathrm{Regime}\;\mathrm{III} & :\;f=-1.44,\; & a=-0.55,\; & b=-0.073,\; & c=0.003,\; & \sigma =0.253. \end{array}$$

**Figure 35:**
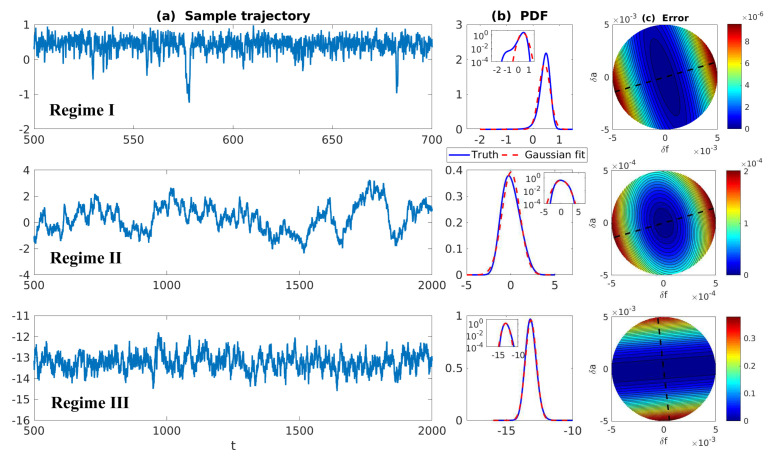
Time series (column (**a**)), equilibrium PDF (column (**b**)) and error due to the parameter perturbation ${\left(\delta f,\delta a\right)}^{T}$ in the two-dimensional parameter space (column (**c**)). The subpanels in column (**b**) are the PDFs in logarithm scale and the red dashed curves are the Gaussian fits. The parameter values of the three regimes are given in (166). The black dashed line shows the direction of the maximum uncertainty, namely the most sensitive direction of perturbation.

The sample trajectories and equilibrium PDFs associated with these regimes are shown in Figure 35.

The PDF in Regime I is unimodal with skewness and an one-side fat tail. Interestingly, the time series in Regime I shows a distinct regimes of behavior [172,173]. Regimes II and III correspond to PC-1 and NAO for the low frequency data as discussed above, where Regime II has a slight skewed PDF with sub-Gaussian tails while Regime III is nearly Gaussian.

With the parameters in (165) and the explicit expression of I(λ) in (164), the most sensitive direction of the parameter perturbation in the two-dimensional space ${\left(\delta f,\delta a\right)}^{T}$ is given by respectively

(166) $$\begin{array}{cc} \mathrm{Regime}\;I & :\;e_{\pi }^{*}={\left(0.9545,0.2981\right)}^{T},\\ \mathrm{Regime}\;\mathrm{II} & :\;e_{\pi }^{*}={\left(0.9685,0.2488\right)}^{T},\\ \mathrm{Regime}\;\mathrm{III} & :\;e_{\pi }^{*}={\left(-0.0760,0.9971\right)}^{T}. \end{array}$$

The results in (166) imply that the forcing perturbation leads to more significant changes of the system in Regimes I and II while damping perturbation is more crucial in Regime III for the NAO. In column (c) of Figure 35, we show the numerical simulations of the relative entropy in (1) with perturbations in all the directions within the entire two-dimensional parameter space ${\left(\delta f,\delta a\right)}^{T}$. Here, we take smaller ${\left(\delta f,\delta a\right)}^{T}$ in Regime II than those in Regimes I and III due to the smaller parameter values ${\left(f,a\right)}^{T}$ in Regime II. These numerical results, which are more expensive to compute, are consistent with the theoretical predictions in (166). Note that, although the most sensitive directions in Regimes I and II are close to each other, the ratio of the larger to smaller eigenvalues in the two regimes are quite different with 18.2979 in Regime I and 2.5307 in Regime II. This means that there is a direction of ${\left(\delta f,\delta a\right)}^{T}$ in Regime I in which the perturbation results in almost no change in the PDF, which can also be seen in column (c) of Figure 35.

Note that both the simple examples shown above contain the perfect knowledge of the present climate given by the unperturbed equilibrium PDFs. However, it is often quite difficult in practice to know the exact expression of these PDFs or it is computationally unaffordable to compute the gradient in high dimensions. Therefore, many approximations are combined with the information theoretical framework developed above. One common practical strategy is to adopt some approximated PDFs based on a few measurements such as the mean and covariance. It is also common to use imperfect or reduced models from a practical point of view, where FDT can also be incorporated to calculate the gradient of the present climate. Then, quantifying the model error in finding the most sensitive directions using imperfect models is an important issue. For detailed discussions of these topics, please see the reference [26].

## 5. Given Time Series, Using Information Theory for Physics-Constrained Nonlinear Stochastic Model for Prediction

### 5.1. A General Framework

A central issue in contemporary science is the development of data-driven statistical dynamical models for the time series of a partial set of observed variables which arise from suitable observations from nature ([174] and references therein). Examples involve multi-level linear autoregressive models as well as ad hoc quadratic nonlinear regression models. It has been established recently [111] that ad hoc quadratic multilevel regression models can have finite time blow up of statistical solutions and pathological behavior of their invariant measure even though they match the data with high precision. Recently, a new class of physics-constrained multi-level nonlinear regression models was developed which involve both memory effects in time as well as physics constrained energy conserving nonlinear interactions [47,48] and completely avoid the above pathological behavior with full mathematical rigor.

The physics-constrained multi-level nonlinear regression models have the following forms:

(167) $$\begin{array}{cc} \frac{du}{dt} & =Lu+B\left(u,u\right)+F+r_{1},\\ \frac{dr}{dt} & =Qu+Ar+\sigma \dot{W}, \end{array}$$

where B(u,u) is a quadratic nonlinearity which imposes the physical constraint of energy conservation on the nonlinear terms, namely

(168) u·B(u,u)=0.

In (167), the noise has the form $r={\left(r_{1},\ldots ,r_{p}\right)}^{T}$ where *p* denotes the number of memory levels and these noises are characterizes by the triangular matrix A. The situation with p=0 denotes the special zero-memory level model

(169) $$\begin{array}{cc} \frac{du}{dt} & =Lu+B\left(u,u\right)+F+\sigma \dot{W}. \end{array}$$

See [47,48] for more details.

**Figure 36:**
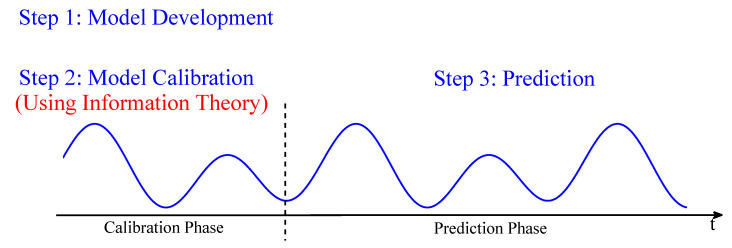
A general procedure for predicting time series.

The ideas of developing physics-constrained nonlinear regression models can be combined with information calibration for predicting strongly nonlinear time series. The general procedure is shown in Figure 36. Here, the observed time series are divided into two parts, namely the training phase and the prediction phase. In the first step, physics-constrained nonlinear stochastic models are developed based on the characteristics of the given time series in the training phase. The second step involves applying information theory for model calibration using the time series again in the training phase. Then, the remaining time series is used for testing the prediction skill of the calibrated model.

### 5.2. Model Calibration via Information Theory

The key step above is the model calibration. As has been seen in Section 2, an effective model is expected to capture both the fidelity and sensitivity of nature. Therefore, two objective functions are utilized here for model calibration. The first one aims at capturing the model fidelity, which is given by minimizing the information distance between the PDF associated with the time series π(u) and that associated with the model $\pi^{M}\left(u\right)$. The model fidelity guarantees the model’s ability in recovering the long-term statistics of nature. However, the model fidelity does not necessarily provide skillful predictions at short and medium ranges. See examples in Figure 9 and Figure 10. Thus, a second objective function is launched, which aims at minimizing the distance between the two autocorrelation functions associated with the observed time series and the model, respectively. As has been shown in Section 2.5, the autocorrelation function is associated with the mean response of the system. In fact, autocorrelation function characterizes the overall time-evolving patterns of the underlying dynamical system. Capturing the autocorrelation function ensures the dynamical consistency and is crucial for skillful short and medium-range forecasts using the proposed model.

Denote θ the parameters in the physics-constrained nonlinear stochastic model. If both the model and nature are stationary, then the model calibration is given by the following optimization problem:

(170) $$L=\min_{\theta }\left(w_{1}P\left(\pi \left(u\right),\pi^{M}\left(u\right)\right)+w_{2}P\left(E\left(\lambda \right),E^{M}\left(\lambda \right)\right)\right),$$

where $w_{1}$ and $w_{2}$ are weight functions. In (170), E(λ) and $E^{M}\left(\lambda \right)$ are the energy spectra corresponding to the autocorrelation functions R(t) and $R^{M}\left(t\right)$ of nature and the model, respectively, as studied in Section 2.5. In practice, time-periodic forcing may be involved in both the observed time series and the physics-constrained model. In such a situation, both $\pi^{M}$ and $R^{M}\left(t\right)$ can be formed by making use of the sample points in a long trajectory from the model. Since the stationary assumption is broken, the target function in (170) can be modified as the average value of the minimizations at different points within one period. Alternatively, an even cruder but practically useful target function involves a modified version of the first part in (170) given by the empirical measurements based on the time-averaged PDFs while the second part in (170) is replaced by directly computing the difference between the two autocorrelation functions. The important issue here is that both the PDF and the temporal correlation must be included in the target function.

The model calibration based on (170) or its modified versions has several salient features. First, the information distance $P(\pi ,\pi^{M})$ is able to quantify the difference in the non-Gaussian statistics between the model and nature. Particularly, it is able to assess the skill of the model in recovering extreme events. Second, the two target functions play the role of improving long-term and short-term prediction skill, respectively. Therefore, the calibrated model can be used for predicting both transit phases and the statistical equilibrium state. Third, although the number of the parameters, namely the dimension of θ, can be large, the cost function *L* is in general robust with respect to the perturbation of θ around the optimal values with a suitable choice of the physics-constrained nonlinear model. This is crucial in practice because it requires only a crude estimation of the parameters for the model, which greatly reduces the computational cost for searching in high-dimensional parameter space. In fact, as has been shown in [46], the energy-conserving nonlinear interaction in these physics-constrained nonlinear models is the underlying mechanism for such robustness property even in the presence of strong nonlinearity and intermittency. Finally, the physics-constrained nonlinear stochastic models require only a short training period [61,175] because the model development automatically involves a large portion of the information of nature. Thus, the data-driven physics-constrained modeling framework as discussed above is much cheaper and more practical than most non-parametric methods where a massive training data is typically required.

### 5.3. Applications: Assessing the Predictability Limits of Time Series Associated with Tropical Intraseasonal Variability

**Figure 37:**
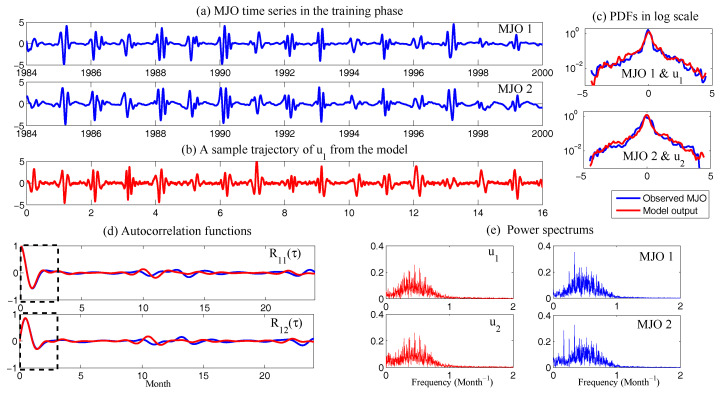
Calibrating the physics-constrained nonlinear stochastic model (171) for the Madden-Julian oscillation (MJO) time series. (**a**): the pair of MJO time series from observations; (**b**): a sample trajectory of $u_{1}$ from the model (171), which has the same length as the MJO time series in panel (**a**); (**c**): comparison of the highly intermittent PDFs in logarithm scale; (**d**): comparison of the autocorrelation functions, where the black dashed box indicates the time range within the first three months; (**e**): comparison of the power spectrums.

A striking application combining physics-constrained nonlinear model strategy with the above procedure is to assess the predictability limits of time series associated with the tropical intraseasonal variability such as the the Madden–Julian oscillation (MJO) and monsoon [46,61,176]. They yield an interesting class of low-order turbulent dynamical systems with extreme events and intermittency. Denote by $u_{1}$ and $u_{2}$ the two observed large-scale components of tropical intraseasonal variability. Here, we focus on the MJO time series [46], which are measured by outgoing longwave radiation (OLR; a proxy for convective activity) from satellite data [177]. See panel (a) of Figure 37. The PDFs for $u_{1}$ and $u_{2}$ (panel (c)) are highly non-Gaussian with fat tails indicative of the temporal intermittency in the large-scale cloud patterns. To describe the variability of the time series $u_{1}$ and $u_{2}$, the following family of low-order stochastic models are proposed:

(171) $$\begin{array}{cc} \frac{du_{1}}{dt} & =\left(-d_{u}u_{1}+\gamma \left(v+v_{f}\left(t\right)\right)u_{1}-\left(a+\omega_{u}\right)u_{2}\right)+\sigma_{u}{\dot{W}}_{u_{1}},\\ \frac{du_{2}}{dt} & =\left(-d_{u}u_{2}+\gamma \left(v+v_{f}\left(t\right)\right)u_{2}+\left(a+\omega_{u}\right)u_{1}\right)+\sigma_{u}{\dot{W}}_{u_{2}},\\ \frac{dv}{dt} & =\left(-d_{v}-\gamma \left(u_{1}^{2}+u_{2}^{2}\right)\right)+\sigma_{v}{\dot{W}}_{v},\\ \frac{d\omega_{u}}{dt} & =\left(-d_{\omega }\omega_{u}\right)+\sigma_{\omega }{\dot{W}}_{\omega }, \end{array}$$

where

(172) $$v_{f}\left(t\right)=f_{0}+f_{t}\sin\left(\omega_{f}t+\phi \right).$$

In (171), in addition to the two observed variables $u_{1}$ and $u_{2}$, the other two variables *v* and $\omega_{u}$ are hidden and unobserved, representing the stochastic damping and stochastic phase, respectively. Here, ${\dot{W}}_{u_{1}},{\dot{W}}_{u_{2}},{\dot{W}}_{v}$ and ${\dot{W}}_{\omega }$ are independent white noise. The constant coefficients $d_{u}$, $d_{v}$, $d_{\omega }$ represent damping for each stochastic process, and the non-dimensional constant γ is the coefficient of the nonlinear interaction. The time periodic damping $v_{f}\left(t\right)$ in the Equation (171) is utilized to crudely model the active winter and the quiescent summer in the annual cycle. The constant coefficients $\omega_{f}$ and ϕ in (172) are the frequency and phase of the damping, respectively. All of the model variables are real. The energy conserving nonlinear interactions between $u_{1},u_{2}$ and $v,\omega_{u}$ are seen in the following way. First, by dropping the linear and external forcing terms in (171), the remaining equations involving only the nonlinear parts of (171) read

(173) $$\begin{array}{cc} \frac{du_{1}}{dt} & =\gamma vu_{1}-\omega_{u}u_{2},\\ \frac{du_{2}}{dt} & =\gamma vu_{2}+\omega_{u}u_{1},\\ \frac{dv}{dt} & =-\gamma (u_{1}^{2}+u_{2}^{2}),\\ \frac{d\omega_{u}}{dt} & =0. \end{array}$$

To form the evolution equation of the energy from nonlinear interactions $E=(u_{1}^{2}+u_{2}^{2}+v^{2}+\omega_{u}^{2})/2$, we multiply the four equations in (173) by $u_{1},u_{2},v,\omega_{u}$ respectively and then sum them up. The resulting equation yields

(174) $$\frac{dE}{dt}=0.$$

The vanishing of the right-hand side in (174) is due to the opposite signs of the nonlinear terms involving *v* multiplying $u_{1}$ and $u_{2}$ in (174) and those in (174) multiplying by *v* as well as the trivial cancellation of skew-symmetric terms involving $\omega_{u}$.

Further motivation for the models in (171) is provided by the stochastic skeleton model which predicts key features of the MJO [178,179,180,181]. These are coupled nonlinear oscillator models of the MJO where if we identify the OLR variables with the envelope of synoptic scale convective activity, the hidden variables $v,\omega_{u}$, and their dynamics become phenomenological surrogates for the energy-conserving interactions in the skeleton model involving the synoptic scale convective activity and the equatorial dynamic equations for temperature, velocity, and moisture.

It is shown in Figure 37 that, with the optimized parameters, the model in (171) almost perfectly capture the highly non-Gaussian fat-tailed PDFs, the autocorrelation functions (up to three months) and the power spectrums. In addition, the wiggles around one year in the autocorrelation functions, representing the annual cycle, are also recovered. Importantly, these parameters are pretty robust around the optimal values. In panel (b), a sample trajectory of $u_{1}$ from the model is shown, which shares many salient features as those of the observed MJO time series in panel (a). Another notable advantage of the physics-constrained nonlinear low-order stochastic models developed here is that the model structure allows an efficient nonlinear data assimilation scheme to determine the initial values of the hidden variables $v,\omega_{u}$ [140]. This facilitates the ensemble prediction algorithm since no direct observation is available for these hidden variables. In [46], significant prediction skill of these MJO indices using the physics-constrained nonlinear stochastic model (171) was shown. The prediction based on ensemble mean can have skill even up to 40 days. In addition, the ensemble spread accurately quantify the forecast uncertainty in both short and long terms. In light of a twin experiment, it was also revealed in [46] that the model in (171) is able to reach the predictability limit of the large-scale cloud patterns of the MJO.

## 6. Reduced-Order Models (ROMs) for Complex Turbulent Dynamical Systems

### 6.1. Strategies for Reduced-Order Models for Predicting the Statistical Responses and UQ

#### 6.1.1. Turbulent Dynamical System with Energy-Conserving Quadratic Nonlinearity

Let’s consider a general framework of turbulent dynamical system [1],

(175) $$\frac{du}{dt}=\left(L+D\right)u+B\left(u,u\right)+F\left(t\right)+\sigma_{k}\left(t\right){\dot{W}}_{k}\left(t;\omega \right).$$

The model in (175) has the following properties:

- L=L+D is a linear operator representing dissipation and dispersion. Here, *L* is skew symmetric representing dispersion and *D* is a negative definite symmetric operator representing dissipative process such as surface drag, radiative damping, viscosity, etc.
- $B\left(u,u\right)$ is a bilinear term and it satisfies energy conserving property with $u\cdot B\left(u,u\right)=0$.

The energy-conserving quadratic nonlinearity is one of the representative features in many turbulent dynamical systems in nature. The energy is transferred from the unstable modes to stable modes where the energy is dissipated resulting in a statistical steady state.

We use a finite-dimensional representation of the stochastic field consisting of a fixed-in-time, *N*-dimensional, orthonormal basis ${\left\{v_{i}\right\}}_{i=1}^{N}$

(176) $$u\left(t\right)=\bar{u}\left(t\right)+\sum_{i=1}^{N}Z_{i}\left(t;\omega \right)v_{i},$$

where $\bar{u}\left(t\right)=\langle u\left(t\right)\rangle$ represents the ensemble average of the response, i.e., the mean field, and $Z_{i}\left(t;\omega \right)$ are stochastic processes. By taking the average of (175) and using (176), the *mean equation* is given by

(177) $$\frac{d\bar{u}}{dt}=\left(L+D\right)\bar{u}+B\left(\bar{u},\bar{u}\right)+R_{ij}B\left(v_{i},v_{j}\right)+F\left(t\right),$$

with $R=\langle ZZ^{*}\rangle$ the covariance matrix. Moreover, the random component of the solution, $u^{\prime }=Z_{i}\left(t;\omega \right)v_{i}$ satisfies

(178) $$\frac{du^{\prime }}{dt}=\left(L+D\right)u^{\prime }+B\left(\bar{u},u^{\prime }\right)+B\left(u^{\prime },\bar{u}\right)+B\left(u^{\prime },u^{\prime }\right)-R_{ij}B\left(v_{i},v_{j}\right)+\sigma_{k}\left(t\right){\dot{W}}_{k}\left(t;\omega \right).$$

By projecting the above equation to each basis element $v_{i}$, we obtain

(179) $$\begin{array}{cc} \frac{dZ_{i}}{dt}=Z_{j}[\left(L+D\right)v_{j}+B\left(\bar{u},v_{j}\right) & +B\left(v_{j},\bar{u}\right)]\cdot v_{i}\\ & +\left[B\left(u^{\prime },u^{\prime }\right)-R_{ij}B\left(v_{i},v_{j}\right)\right]\cdot v_{i}+\sigma_{k}\left(t\right){\dot{W}}_{k}\left(t;\omega \right)\cdot v_{i}. \end{array}$$

From the last equation, we directly obtain the exact *evolution equation of the covariant matrix* $R=\langle ZZ^{*}\rangle$

(180) $$\frac{dR}{dt}=L_{v}R+RL_{v}^{*}+Q_{F}+Q_{\sigma },$$

where we have:

1. The linear dynamical operator expressing energy transfers between the mean field and the stochastic modes (effect due to *B*), as well as energy dissipation (effect due to *D*) and non-normal dynamics (effect due to *L*)
  (181) $${\left\{L_{v}\right\}}_{ij}=\left[\left(L+D\right)v_{j}+B\left(\bar{u},v_{j}\right)+B\left(v_{j},\bar{u}\right)\right]\cdot v_{i}.$$
2. The positive definite operator expressing energy transfer due to the external stochastic forcing
  (182) $${\left\{Q_{\sigma }\right\}}_{ij}=\left(v_{i}\cdot \sigma_{k}\right)\left(\sigma_{k}\cdot v_{j}\right).$$
3. The energy flux between different modes due to non-Gaussian statistics (or nonlinear terms) modeled through third-order moments
  (183) $${\left\{Q_{F}\right\}}_{ij}=\langle Z_{m}Z_{n}Z_{j}\rangle B\left(v_{m},v_{n}\right)\cdot v_{i}+\langle Z_{m}Z_{n}Z_{i}\rangle B\left(v_{m},v_{n}\right)\cdot v_{j}.$$

We note that the energy conservation property of the quadratic operator *B* is inherited by the matrix $Q_{F}$ since

(184) $$\mathrm{tr}\left(Q_{F}\right)=2\langle Z_{m}Z_{n}Z_{i}\rangle B\left(v_{m},v_{n}\right)\cdot v_{i}=2B\left(u^{\prime },u^{\prime }\right)\cdot u^{\prime }=0.$$

Based on the observation that the eigenvalues are effectively changed by the existence of the nonlinear energy transfer mechanism, we propose a special form of the flux $Q_{F}$ that will make the correct steady state statistics a stable equilibrium. More specifically, we split the nonlinear fluxes into a positive semi-definite part $Q_{F,+}$ and a negative semi-definite part $Q_{F,-}$:

$$Q_{F}=Q_{F,-}+Q_{F,+}.$$

As in (184), the nonlinear fluxes should always satisfy the conservative property of *B*, namely,

$$\mathrm{tr}\left[Q_{F}\right]=0⟹\mathrm{tr}\left[Q_{F,-}\right]=-\mathrm{tr}\left[Q_{F,+}\right].$$

The positive fluxes $Q_{F,+}$ indicate the energy being `fed’ to the stable modes in the form of external stochastic noise. On the other hand, the negative fluxes $Q_{F,-}$ should act directly on the linearly unstable modes of the spectrum, effectively stabilizing the unstable modes.

#### 6.1.2. Modeling the Effect of Nonlinear Fluxes

The first idea here is to model the effect of the nonlinear energy transfers on each mode by adding additional damping balancing the linearly unstable character of these modes, and adding additional (white) stochastic excitation with standard deviation which will model the energy received by the stable modes,

(185) $$Q_{F}^{M}=Q_{F,-}^{M}+Q_{F,+}^{M}=-D_{M}\left(R\right)R_{M}-R_{M}D_{M}^{*}\left(R\right)+\Sigma_{M}\left(R\right).$$

In (185), $\left(D_{M},\Sigma_{M}\right)$ are N×N matrices that replace the original nonlinear unstable and stable effects from the original dynamics. Here $Q_{F,-}^{M}=-D_{M}\left(R\right)R_{M}-R_{M}D_{M}^{*}\left(R\right)$ represents the additional damping effect to stabilize the unstable modes with positive Lyapunov coefficients, while $Q_{F,+}^{M}=\Sigma_{M}\left(R\right)$ is the positive-definite additional noise to compensate for the overdamped modes. Now, the problem is converted to finding expressions for $D_{M}$ and $\Sigma_{M}$. In the following, by gradually adding more detailed characterization about the statistical dynamical model, we display the general procedure of constructing a hierarchy of the closure methods step by step. Below is a review about several model closure ideas [1,11,50,117] with increasing complexity:

1. *Quasilinear Gaussian closure model:* The simplest approximation for the closure methods at the first stage should be simply neglecting the nonlinear part entirely [182,183,184]. That is, set
  (186) $$D_{M}\left(R\right)\equiv 0,\;\Sigma_{M}\left(R\right)\equiv 0,\;Q_{F}^{QG}\equiv 0.$$
  Thus, the nonlinear energy transfer mechanism will be entirely neglected in this Gaussian closure model. This is the similar idea in the eddy-damped Markovian model where the moment hierarchy is closed at the level of second moments with Gaussian assumption and a much larger eddy-damped parameter is introduced to replace the molecular viscosity [121,185]. Obviously, this crude Gaussian approximation will not work well in general due to the cutoff of the energy flow when strong nonlinear interactions between modes occur. Actually, the deficiency of this crude approximation has been shown under the Lorenz 96 framework, and in a final equilibrium state, there exists only one active mode with a critical wavenumber [11,50]. Such closures are only useful in the weakly nonlinear case where the quasi-linear effects are dominant.
2. *Models with consistent equilibrium statistics:* The next strategy is to construct the simplest closure model with consistent equilibrium statistics. Thus, the direct way is to choose constant damping and noise term scaled with the total variance. We propose two possible choices as in [50] for the damping and noise in (185) below.
  **Gaussian closure 1 (GC1)**: let
  (187) $$D_{M}\left(R\right)=\epsilon_{M}I_{N}\equiv \mathrm{const}.,\;\;\;\Sigma_{M}\left(R\right)=\sigma_{M}^{2}I_{N}\equiv \mathrm{const}.,\;\;\;Q_{F}^{GC1}=-\left(\epsilon_{M}R+R\epsilon_{M}\right)+\sigma_{M}^{2}I_{N}.$$
  **Gaussian closure 2 (GC2)**: let
  (188) $$\begin{array}{c} D_{M}\left(R\right)=\epsilon_{M}{\left(\frac{trR}{trR_{eq}}\right)}^{1/2}I_{N},\;\Sigma_{M}\left(R\right)=\sigma_{M}^{2}{\left(\frac{trR}{trR_{eq}}\right)}^{3/2}I_{N},\\ Q_{F}^{GC2}=-{\left(\frac{trR}{trR_{eq}}\right)}^{1/2}\left(\epsilon_{M}R+R\epsilon_{M}\right)+\sigma_{M}^{2}{\left(\frac{trR}{trR_{eq}}\right)}^{3/2}I_{N}. \end{array}$$
  Above, only two scalar model parameters $\left(\epsilon_{M},\sigma_{M}\right)$ are introduced, and $I_{N}$ represents the N×N identity matrix. GC1 is the familiar strategy of adding constant damping and white noise forcing to represent nonlinear interaction; GC2 scales with the total variance trR (or total statistical energy) so that the model sensitivity can be further improved as the system is perturbed. From both GC1 and GC2, we introduce uniform additional damping rate for each spectral mode controlled by a single scalar parameter $\epsilon_{M}$, while the additional noise with variance $\sigma_{M}^{2}$ is added to make sure climate fidelity in equilibrium.
  The statistical model closure $Q_{F}^{M}$ is used to approximate the third-order moments in the true dynamics, thus the exponents of the total energy trR in GC2 should be consistent in scaling dimension. In the positive-definite part $Q_{F+}^{M}$, it calibrates the rate of energy injected into the spectral mode due to nonlinear effect in the order $|u^{\prime }{|}^{3}$. The factor scales with the total energy with exponent 3/2 so that the corrections keep consistent with the third-order moment approximations; In the negative damping rate $Q_{F,-}^{M}$, the scaling function is used to characterize the amount of energy that flows out the spectral mode due to nonlinear interactions. Scaling factor with a square-root of the total energy with exponent 1/2 is applied for this damping rate multiplying the variance in order $|u^{\prime }{|}^{2}$ to make it consistent in scaling dimension with third moments.
  However, the damping and noise are chosen empirically without consideration about the true dynamical features in each mode. A more sophisticated strategy with slightly more complexity in computation is to introduce the damping and noise judiciously according to the linearized dynamics. Then, climate consistency for each mode can be satisfied automatically.
3. *Modified quasi-Gaussian (MQG) closure with equilibrium statistics:* In this modified quasi-Gaussian closure model originally proposed in [11,45], we exploit more about the true nonlinear energy transfer mechanism from the equilibrium statistical information. Thus, the additional damping and noise proposed as before are calibrated through the equilibrium nonlinear flux by letting
  (189) $$D_{M}\left(R\right)=-N_{M,eq},\;\Sigma_{M}\left(R\right)=Q_{F,eq}^{+},\;Q_{F}^{MQG}=-\left(N_{M}R+RN_{M}^{*}\right)+Q_{F}^{+}.$$
  $N_{M,eq}$ is the effective damping from equilibrium, and $Q_{F,eq}^{+}$ is the effective noise from the positive-definite component. Unperturbed equilibrium statistics in the nonlinear flux $Q_{F,eq}$ are used to calibrate the higher-order moments as additional energy sink and source. The true equilibrium higher-order flux can be calculated without error from first and second order moments in $\left({\bar{u}}_{eq},R_{eq}\right)$ from the unperturbed true dynamics (180) in steady state following the steady state statistical solution relation:
  (190) $$Q_{F,eq}=Q_{F,eq}^{-}+Q_{F,eq}^{+}=-L_{v}\left({\bar{u}}_{eq}\right)R_{eq}-R_{eq}L_{v}{\left({\bar{u}}_{eq}\right)}^{*}-Q_{\sigma },\;N_{M,eq}=\frac{1}{2}Q_{F,eq}^{-}R_{eq}^{-1},$$
  where $Q_{F,eq}^{-},Q_{F,eq}^{+}$ are the negative and positive definite components in the unperturbed equilibrium nonlinear flux $Q_{F,eq}$. Since exact model statistics are used in the imperfect model approximations, the true mechanism in the nonlinear energy transfer can be modeled under this first correction form. This is the similar idea used for measuring higher order interactions in [45], where more sophisticated and expensive calibrations are required to make that model work there.

#### 6.1.3. A Reduced-Order Statistical Energy Model with Optimal Consistency and Sensitivity

The above closure model ideas, especially (187)–(189), have advantages of their own. Models in (187) and (188) are simple and efficient to construct with consistent equilibrium consistency, while (189) involves the true information about the higher-order statistics in equilibrium so that the energy mechanism can be characterized well. The validity of these approaches has been tested and compared from several papers [11,45,50] using the simplified triad model and L96 model. The methods have also been applied to the two-layer quasi-geostrophic equation [44,117], where the phase space of the original system is $5\times 10^{5}$ while the ROM contains only 0.1% of the large scale modes and can cope with the changes in external forcing. Still, when it comes to the more complicated and realistic flow systems such as the quasi-geostrophic equations, more detailed calibration for model consistency and sensitivity is required to achieve the optimal performance. A preferred approach for the nonlinear flux $Q_{F}^{M}$ combining both the detailed model energy mechanism and control over model sensitivity is proposed in the form

(191) $$Q_{F}^{M}=Q_{F}^{M,-}+Q_{F}^{M,+}=f_{1}\left(R\right)\left[-\left(N_{M,eq}+d_{M}I_{N}\right)R_{M}\right]+f_{2}\left(R\right)\left[Q_{F,eq}^{+}+\Sigma_{M}\right].$$

The closure form (191) consists of three indispensable components:

(i). *Higher-order corrections from equilibrium statistics:* In the first part of the correction using the damping and noise operator as $\left(N_{M,eq},Q_{F,eq}^{+}\right)$, unperturbed equilibrium statistics in the nonlinear flux $Q_{F,eq}$ are used to calibrate the higher-order moments as additional energy sink and source following the procedure in (189). Therefore, the equilibrium statistics can be guaranteed to be consistent with the truth, and the true energy mechanism can be restored.
(ii). *Additional damping and noise to model changes in nonlinear flux:* The above corrections in step (i) by using equilibrium information for nonlinear flux is found to be insufficient for accurate prediction in the reduced-order methods since the scheme is only marginally stable and the energy transferring mechanism may change with large deviation from the equilibrium case when external perturbations are applied. Thus, we also introduce the additional damping and noise $\left(d_{M},\Sigma_{M}\right)$ as from (187). $d_{M}$ is just a constant scalar parameter to add uniform dissipation on each mode, and $\Sigma_{M}$ is the further correction as an additional energy source to maintain climate fidelity.
(iii). *Statistical energy scaling to improve model sensitivity:* Still note that these additional parameters are added regardless of the true nonlinear perturbed energy mechanism where only unperturbed equilibrium statistics are used. To capture the responses to a specific perturbation forcing, it is better to make the imperfect model parameters change adaptively according to the total energy structure. Considering this, the additional damping and noise corrections are scaled with factors $f_{1}\left(R\right),f_{2}\left(R\right)$ related with the total statistical variance trR as>
  (192) $$f_{1}\left(R\right)={\left(\frac{trR}{trR_{eq}}\right)}^{1/2},\;f_{2}\left(R\right)={\left(\frac{trR}{trR_{eq}}\right)}^{3/2}.$$

#### 6.1.4. Calibration Strategy

As discussed in the previous sections, the calibration should involve two criteria: (1) the model fidelity (consistency) with the same equilibrium statistics as the truth, and (2) the optimal model sensitivity.

Let’s denote $\pi_{G}\left(u\right)$ and $\pi_{G}^{M}\left(u\right)$ the Gaussian distributions of the truth and the imperfect model, as in Section 2.1. Here, the Gaussian approximation of the PDFs is adopted since the above reduced-order model strategy only involves the evolution of mean and variance in the imperfect model. According to the information-theoretic framework in Section 2.1, the statistical equilibrium fidelity means that the Gaussian relative entropy satisfies

(193) $$P(\pi_{G}\left(u\right),\pi_{G}^{M}\left(u\right))=0.$$

Practically, we can make use of the explicit form (6) to calibrate the parameters such that (193) is satisfied. Statistical equilibrium fidelity is a natural necessary condition to tune the mean and variance of the imperfect model to match those of the perfect model; it is far from a sufficient condition. To see this, recall from Section 2.5 that different dynamical systems can have the same Gaussian invariant measure and therefore statistical equilibrium fidelity among the models is obviously satisfied (see [40] for several concrete examples). Thus, the condition in (193) should be regarded as an important necessary condition. UQ requires an accurate assessment of both the mean and variance and at least (193) guarantees calibration of this on a subspace, $u\in R^{M}$, for the unperturbed model. Climate scientists often just tune only the means (see [26] and references therein).

Next, the prediction skill of imperfect models can be improved by comparing the information distance through the linear response operator with the true model. The response in the Gaussian framework $P(\pi_{\delta },\pi_{\delta }^{M})$ can be computed by making use of (10). This condition has been shown to be crucial in calibrating the parameters (see examples in Section 2.5 and Section 5). The optimal model $M^{*}\in M$ that ensures best information consistent responses to various kinds of perturbations is characterized with the smallest additional information in the linear response operator R among all the imperfect models, such that

(194) $${∥P\left(\pi_{\delta },\pi_{\delta }^{M^{*}}\right)∥}_{L^{1}\left(\left[0,T\right]\right)}=\min_{M\in M}{∥P\left(\pi_{\delta },\pi_{\delta }^{M}\right)∥}_{L^{1}\left(\left[0,T\right]\right)},$$

where $\pi_{\delta }^{M}$ can be achieved through a kicked response procedure (138) in the training phase compared with the actual observed data $\pi_{\delta }$ in nature, and the information distance between perturbed responses $P\left(\pi_{\delta },\pi_{\delta }^{M}\right)$ can be calculated with ease through the expansion formula (10). For the time-periodic cases, the information distance $P\left(\pi_{\delta }\left(t\right),\pi_{\delta }^{M}\left(t\right)\right)$ is measured at each time instant, so the entire error is averaged under the $L^{1}$-norm inside proper time window $\left[0,T\right]$ (such as one period). Some low dimensional examples of this procedure for turbulent systems can be found in [10,30,186].

Below, in the example of predicting passive tracer extreme events using low-order reduced model (Section 6.3), the imperfect models are all linear Gaussian models. As we have already seen in Section 2.2.1 and Section 2.3, the linear Gaussian models are only able to capture the response in the mean statistics. Therefore, minimizing the model sensitivity is based on optimizing the mean response in the imperfect models compared with that in the truth. Note that optimizing the mean response is equivalent to optimizing the autocorrelation function in the linear Gaussian models. Thus, instead of applying the general strategy with FDT for the optimization of the response, minimizing the spectral density (for autocorrelations) using information theory as discussed in Section 2.5 is an efficient alternative approach, which will be adopted below. The readers should keep in mind that these two methods share the same goal of optimizing the sensitivity in imperfect models.

### 6.2. Physics-Tuned Linear Regression Models for Hidden (Latent) Variables

Before we show concrete applications of the reduced-order modeling framework developed in Section 6.1, let’s briefly discuss the physics-tuned linear regression modeling strategy. These physics-tuned linear regression models are particularly useful for simplifying the hidden or latent processes in complex dynamical systems while they preserve the important feedback from the latent variables to the resolved variables. Thus, such physics-tuned linear regression technique allows the dynamics and statistical structure of the models to become much more tractable and facilitates the application of the reduced-order modeling strategy in Section 6.1.

Consider the following general nonlinear system,

(195) $$\begin{array}{cc} \frac{du}{dt} & =F_{1}\left(u,v\right)+\sigma_{u}{\dot{W}}_{u},\\ \frac{dv}{dt} & =F_{2}\left(u,v\right)+\sigma_{v}{\dot{W}}_{v}, \end{array}$$

where $F_{1}$ and $F_{2}$ are nonlinear functions of u and v. The model in (195) is usually written as a collection of Fourier modes. In (195), the state variables u are resolved variables that are our primary interest. The state variables v are the latent or unresolved variables, which nevertheless play an important role in contributing to the variability of u through nonlinear interactions. Here, the goal is to develop physics-tuned linear regression models of v such that their dynamics and statistical structure become much more tractable. Meanwhile, the feedback from v to u under the physics-tuned linear regression modeling framework are expected to be preserved to a large extent. The physics-tuned linear regression modeling framework for the latent variables *v* is given as follows:

(196) $$\begin{array}{cc} \frac{du^{M}}{dt} & =F_{1}\left(u^{M},v^{M}\right)+\sigma_{u}{\dot{W}}_{u},\\ \frac{dv^{M}}{dt} & =\Lambda^{M}v^{M}+\left(v^{M}-{\hat{v}}^{M}\right)+\sigma_{v}^{M}{\dot{W}}_{v}^{M}, \end{array}$$

where $\Lambda^{M}$ are $\sigma_{v}^{M}$ both diagonal matrices. The *i*-th diagonal entry of $\Lambda^{M}$ usually has the form $\lambda_{j}^{M}=-d_{j}^{M}+i\omega_{j}^{M}$, where the real part of each diagonal entry of $\lambda_{j}^{M}$, namely $-d_{j}^{M}$, is negative. In other words, each component $v_{j}$ of v satisfies a one-dimensional OU process:

(197) $$\frac{dv_{j}^{M}}{dt}=\lambda_{j}^{M}v_{j}^{M}+\left(v_{j}^{M}-{\hat{v}}_{j}^{M}\right)+\sigma_{v,j}^{M}{\dot{W}}_{v,j}^{M}.$$

The physics-tuned linear regression modeling strategy requires that each $v_{j}^{M}$ in (197) satisfies both the model fidelity and model sensitivity compared with the *i*-th component of the truth v, namely $v_{j}$, in (195). Therefore, the model parameters $d_{j}^{M},\omega_{j}^{M},{\hat{v}}_{j}^{M}$ and $\sigma_{v,j}^{M}$ in (197) are calibrated by matching the equilibrium Gaussian PDF and the autocorrelation function with those associated with $v_{j}$ in (195). See Section 2.5 for more technical details. Note that the goal here is to let $v^{M}$ provide the least biased statistical feedback to u instead of recovering all the point-wise details of the latent random process $v^{M}$.

Below, we use a simple example to illustrate physics-tuned linear regression modeling strategy and emphasize the importance of capturing the feedback from v to u. Note that such ideas will be adopted in Section 6.3 for predicting the extreme events in passive tracers, where v and u can be thought of as the surrogates of the advection flow and the passive tracer fields there, respectively.

The true model is a two-dimensional nonlinear model:

(198) $$\begin{array}{cc} \frac{du}{dt} & =\left(-v+i\omega_{u}\right)u+\sigma_{u}{\dot{W}}_{u},\\ \frac{dv}{dt} & =\left(f+av+bv^{2}-cv^{3}\right)+\left(A-Bv\right){\dot{W}}_{C}+\sigma_{v}{\dot{W}}_{A}. \end{array}$$

In (198), *u* is complex but *v* is real. The unresolved process *v* is given by the canonical model for low frequency atmospheric variability, derived based on stochastic mode reduction strategies [116,170]. It has been used in Section 4.3. The unresolved variable *v* serves as the stochastic damping for the resolved variable *u*. The feedback mechanism of *v* with suitable parameters can result in the intermittent instability of *u*. The parameters in this coupled model are all constants. We consider the following two dynamical regimes:

(199) Fat-tailedregime:a=-2.20,b=-2.6,c=0.8,A=1.0,B=-2.0,f=-2.0,σv=2.Bimodalregime:a=-2.64,b=-7.8,c=4.0,A=0.1,B=-0.1,f=-0.2,σv=2,

where the fat-tailed regime is a typical non-Gaussian regime for the unresolved process while the bimodal one is an extremely tough test regime. The other two parameters in the dynamics of *u* are the same in the two regimes,

(200) $$\sigma_{u}=0.1,\;\omega_{u}=2.$$

The reduced model with simplified process of *v* is given by

(201) $$\begin{array}{cc} \frac{du^{M}}{dt} & =\left(-v^{M}+i\omega_{u}\right)u^{M}+\sigma_{u}{\dot{W}}_{u},\\ \frac{dv^{M}}{dt} & =-d_{v}^{M}\left(v^{M}-{\hat{v}}^{M}\right)+\sigma_{v}^{M}{\dot{W}}_{v}^{M}. \end{array}$$

Since *v* is real in the true model (198), $v^{M}$ is also real in the reduced model (201). Thus, $\lambda_{j}^{M}\equiv -d_{v}^{M}$ here, which is a special case of the general framework in (197). The three parameters $d^{M}$, ${\hat{v}}^{M}$ and $\sigma_{v}^{M}$ are tuned by capturing the model sensitivity (autocorrelation function) and model fidelity (equilibrium PDF) of the those associated with the truth of *v* in (198).

**Figure 38:**
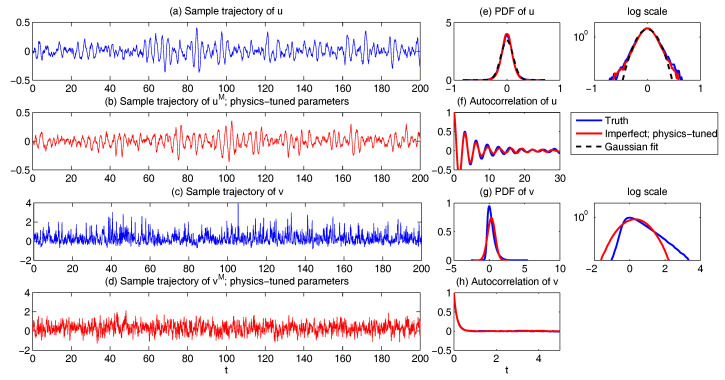
(**a**,**c**): sample trajectories of the truth of *u* and *v* in the regime where *v* has a fat-tailed PDF. (**b**,**d**): sample trajectories of $u^{M}$ and $v^{M}$ from the reduced model with physics-tuned parameters; (**e**,**g**): comparison of the PDFs of *u* and *v* in the truth and physics-tuned model. The right panels show the PDFs in the logarithm scale; (**f**,**h**): comparison of the autocorrelation function. All the trajectories and statistics with respect to *u* means the real part of *u*.

**Figure 39:**
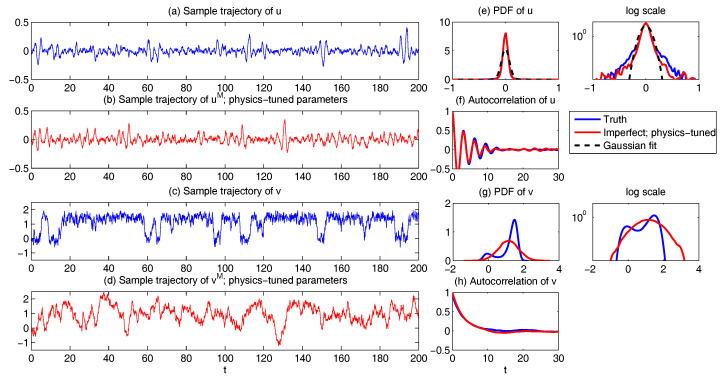
Same as Figure 38 but in bimodal regime of *v*.

**Figure 40:**
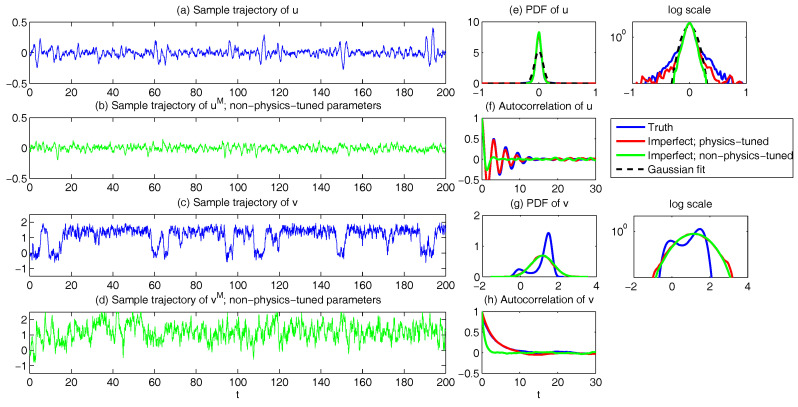
As in Figure 39 where the process of the truth of *v* is in a bimodal regime. In the reduced model, the parameters are not physical-tuned. Thus, a large model error is seen from both the trajectories in (**b**,**d**) compared with the truth (**a**,**c**) and the statistics in (**e**–**h**).

The sample trajectories and the statistics of both the truth and the reduced model with the physics-tuned parameters are shown in Figure 38 and Figure 39 for the fat-tailed and the bimodal regimes, respectively. In both figures, despite the failure in capturing the non-Gaussianity in the hidden process *v*, the non-Gaussian fat-tailed PDFs as well as the intermittent trajectories of the resolved variable *u* are both recovered with high accuracy in the reduced model with physics-tuned parameters. One crucial reason for the high skill in the reduced model is that the autocorrelation function of $v^{M}$ resembles that of *v* in the truth. Therefore, the duration and frequency of the intermittent phases of $u^{M}$ are statistically similar to those of *u*. In fact, even in the bimodal regime which is an extremely tough test case (Figure 39), where the PDF of $v^{M}$ is highly biased from that of *v*, the feedback mechanism from *v* to *u* is well recovered by the reduced model due to the capturing of both the model fidelity and model sensitivity. In Figure 40, we show that only matching the equilibrium PDF of $v^{M}$ with that of *v* but ignoring the autocorrelation function in the calibration process is insufficient in recovering the key features of *u*. This emphasizes the importance of physics-tuned calibration strategy and the skill of using the resulting linear regression model for the hidden unresolved variables in capturing the non-Gaussian intermittent behavior of the resolved variable *u*.

### 6.3. Predicting Passive Tracer Extreme Events

Turbulent diffusion models of passive tracers have numerous applications in geophysical science and engineering. These applications range from, for example, the spread of contaminants or hazardous plumes in environmental science, to the behavior of anthropogenic and natural tracers in climate change science, and many related areas [187,188,189,190]. The scalar field T(x,t) describes the concentration of the passive tracer immersed in the fluid which is carried with the local fluid velocity but which does not itself significantly influence the dynamics of the fluid. The evolution of the passive tracer is driven by turbulent advection, diffusion and usually uniform damping,

(202) $$\frac{\partial T}{\partial t}+v\cdot \nabla T=-d_{T}T+\kappa \Delta T,$$

where v(x,y,t) is a velocity field. One key feature of great interest in the tracer turbulent model (202) is the existence of intermittency, which can be found in atmosphere observational data [190], laboratory experiments [191], and numerical simulations of idealized models [15,189,192,193].

A special form of the velocity field v, which is a superposition of a spatially uniform but possibly temporally fluctuating cross-sweep in the *x*-direction and a random shear flow (with fluctuations possibly in both time and spatial *x*-direction) in the *y*-direction

(203) v(x,y,t)=(U(t),v(x,t)),

has been proposed by Majda et al. [189,193] and tested on simple mathematical models [26,29,194]. Assume the existence of a background mean gradient for the tracer varying in only *y*-variable and a tracer fluctuation component dependent with only the *x*-variable

(204) $$T\left(x,t\right)=T^{\prime }\left(x\right)+\alpha y.$$

Combining (204) with the tracer dynamics (202) and the simplified flow field (203), the fluctuation part of the tracer $T^{\prime }$ satisfies the following equation:

(205) $$\frac{\partial T^{\prime }}{\partial t}+U\left(T\right)\frac{\partial T^{\prime }}{\partial x}=-\alpha v\left(x,t\right)-d_{T}T^{\prime }+\kappa \frac{\partial^{2}T^{\prime }}{\partial x^{2}}.$$

Despite their simplicity, the model (205) in random shear flow with a mean sweep can capture and preserve key features for various inertia range statistics for turbulent diffusion [192,195,196,197,198] including intermittency. Even for roughly Gaussian velocity fields *v* in (203) as observed in turbulent flows, the linear scalar field can experience rare but very large fluctuations in amplitude, and its statistics can depart significantly from Gaussianity displaying fatter tails representing the intermittency [199,200,201,202,203]. Explicit formulations about the statistical solutions of the tracers have been derived in [193] under this simplified flow system, and a rigorous mathematical proof about the intermittent fat tails in tracer distributions has been achieved recently in [195].

Complex nonlinear and non-Gaussian features in the flow components are unavoidable and ubiquitous especially in realistic turbulent flows. The complexity and large computational expense in resolving the highly turbulent advection flow equations require the introduction of simpler and more tractable imperfect models while still maintaining the ability in capturing the key intermittent features in the tracer field. Below, we investigate the effects from a nonlinear advection flow on the steady state passive tracer intermittency, and especially the errors and performances of imperfect approximation models are tested in a variety of turbulent regimes. In particular, the following two issues will be addressed in the remaining of this section:

1. Whether a linear Gaussian dynamics in approximating the advection flow is able to capture tracer non-Gaussian statistical structures?
2. How to design an unambiguous reduced-order stochastic modeling strategy with high prediction skill of the tracer field?

There are at least two motivations for using linear Gaussian imperfect models for describing advection flow *v*. First, the dynamics and statistical structure become much more tractable with explicit solutions that enable us to design the model and tune parameters with ease. Second, the computational difficulty and cost are also greatly reduced considering the simple and controllable structure of the linear model. However, it is challenging by applying these linear Gaussian models with no positive Lyapunov exponents to estimate the non-Gaussian flow field including various degrees of internal instabilities. Therefore, a systematic procedure in calibrating the imperfect model parameters is required.

Here, the information-theoretic framework developed in Section 6.1.4 is applied to train the imperfect model parameters in a training phase so that the model predicted stationary process can possess the least biased estimation in energy and autocorrelation function, the latter of which plays an particularly important role in determining the structure of tracer statistics. With such a systematical calibration strategy, these linear stochastic models can be greatly improved through this proposed tuning strategy under a proper information metric. On the other hand, the reduced-order strategy aims at using low order equilibrium statistics in the model calibration as a correction to the nonlinear small scale feedback, which avoids high computational cost.

#### 6.3.1. Approximating Nonlinear Advection Flow Using Physics-Tuned Linear Regression Model

Here, we aim at answering the question that whether a linear Gaussian dynamics is able to approximate the advection flow such that the non-Gaussian statistical structures in the tracer field is preserved. One good reference of this topic is [50].

To this end, we consider a background flow, which is driven by the 40-dimensional Lorenz 96 (L96) system [204]. The L96 model is able to mimic baroclinic turbulence in the midlatitude atmosphere with the effects of energy conserving nonlinear advection and dissipation, displaying a wide range of distinct dynamical regimes from Gaussian to extremely non-Gaussian statistics. Therefore, the L96 model is a representative testbed for studying the model error here.

The L96 system with state variables $u\left(x,t\right)={\left(u_{0},u_{1},\ldots u_{J-1}\right)}^{T}$ is given by

(206) $$\frac{du_{j}}{dt}=\left(u_{j+1}-u_{j-2}\right)u_{j-1}-d_{u}\left(t\right)u_{j}+F\left(t\right),\;j=0,1,\ldots ,J-1,\;J=40,$$

with periodic boundary condition $u_{0}=u_{J}$. Nonlinearity comes from the bilinear quadratic form $B_{j}\left(u,u\right)=\left(u_{j+1}-u_{j-2}\right)u_{j-1}$, which conserves energy through u·B(u,u)=0. By changing the amplitude of the external forcing *F*, the L96 system displays a wide range of different dynamical regimes ranging from weakly chaotic (F=5), strongly chaotic (F=8), to finally full turbulence (F=16) with varying statistics.

The advection flow field v=(U(t),v(x,t)) is constructed from the L96 model solution. Note that the system is homogeneous and translation invariant along each grid point, so standard Fourier basis $e_{k}={\left\{e^{2\pi ikj/J}\right\}}_{j=1}^{J-1}$ naturally becomes the empirical orthogonal functions (EOFs) of the system [50]. The state variables of the system can be decomposed under Fourier basis as

(207) $$u\left(x,t\right)=\bar{u}\left(t\right)+\sum_{k=-J/2+1}^{J/2}{\hat{u}}_{k}\left(t\right)e_{k}\left(x\right),\;\langle {\hat{u}}_{k}\rangle =0,\;{\hat{u}}_{-k}={\hat{u}}_{k}^{*},$$

where 〈·〉 is the ensemble average. We construct the passive tracer fields nonlinearly advected by the flow generated through the L-96 system. The gradient cross-sweeping component U(t) is from the mean state with randomness from zero mode, while the shearing component $v(x_{j},t)$ simulated by the flow fluctuation modes with varying values at each grid point,

(208) $$U\left(t\right)=\bar{u}\left(t\right)+{\hat{u}}_{0}\left(t\right),\;v\left(x_{j},t\right)=\sum_{k\neq 0}{\hat{u}}_{k}\left(t\right)e^{2\pi ikx_{j}}.$$

Below, we will focus on the statistical features of the scalar tracer field in stationary steady state. To make sure the system converges to the final stationary state, that is, $\bar{u}\left(t\right)\to {\bar{u}}_{\infty }$, $r_{k}\left(t\right)=\langle |{\hat{u}}_{k}{|}^{2}\left(t\right)\rangle \to r_{k,\infty }$ as t→∞, we consider the simplified dynamics of (206) with constant damping and forcing terms $d_{u}\equiv d_{u}\left(t\right),F\equiv F\left(t\right)$.

The exact dynamical equations for each mode in the shearing flow ${\hat{u}}_{k}$ and the mean gradient *U* can be derived from the L96 system (206) as

(209) $$\begin{array}{cc} \frac{dU}{dt} & =-d_{u}U\left(t\right)+\sum_{k\neq 0}\Gamma_{k}{\left|{\hat{u}}_{k}\right|}^{2}\left(t\right)+F, \end{array}$$

(210) $$\begin{array}{cc} \frac{d{\hat{u}}_{k}}{dt} & =-d_{u}{\hat{u}}_{k}+\left(e^{2\pi i\frac{k}{j}}-e^{-2\pi i\frac{2k}{j}}\right)U\left(t\right){\hat{u}}_{k}+\sum_{m\neq 0}{\hat{u}}_{k+m}{\hat{u}}_{m}^{*}\left(e^{2\pi i\frac{2m+k}{J}}-e^{-2\pi i\frac{m+2k}{J}}\right), \end{array}$$

where k=1,…,J/2 and the energy transfer rate is $\Gamma_{k}=\cos\frac{4\pi k}{J}-\cos\frac{2\pi k}{J}$. The cross-sweep field U is forced by the combined effects from each fluctuation mode $\sum_{k\neq 0}\Gamma_{k}{\left|{\hat{u}}_{k}\right|}^{2}$, and conversely the shearing flow is advected by the mean drift through the second term in the first line in (210).

The accuracy in the steady state passive tracer statistics is limited by the modeling and computation skill of the complex background advection flow *v*. However, several difficulties cannot be bypassed if we directly go with the true flow system with nonlinearity. First, simple Galerkin truncation of high frequency wave-numbers in the dynamical equations may introduce large errors to the flow system due to strong nonlinear interactions between the (truncated) small scale and large scale modes. Second, even with a low dimensional Galerkin truncation model, large ensemble size may still be required to resolve the flow if non-Gaussian features and intermittencies are important and of interest. On the other hand, returning to our original problem, the central issue of major interest is the turbulent fluctuation and statistical structure of the passive tracer *T* rather than the background flow field *v*. Considering both sides of the problem, the question that is worth asking is whether we can predict the crucial features (such as, intermittency) in steady state tracer statistics advected and forced by nonlinear non-Gaussian background flow *v* using simpler imperfect models with error for the background dynamical field.

Now, we adopt the simplest approximation about the advection flow with imperfect models using linear stochastic dynamics along each spectral mode from the Ornstein–Uhlenbeck process [15,193,196,205]. With the simple structures in these linear Gaussian models, the dynamics and statistical structure become much more tractable with explicit solutions that enable us to design the model and tune parameters with ease. The linear stochastic models for each mode can be written as

(211) $$\frac{d{\hat{u}}_{k}^{M}}{dt}=\left(-\gamma_{u_{k}}+i\omega_{u_{k}}\right){\hat{u}}_{k}^{M}+\sigma_{u_{k}}{\dot{W}}_{k},$$

with $\gamma_{u_{k}},\omega_{u_{k}}$ and $\sigma_{u_{k}}$ as parameters to be determined, together with the dynamics for the mean

(212) $$\frac{d{\bar{u}}^{M}}{dt}=-d_{u}{\bar{u}}^{M}+\sum_{k\neq 0}\Gamma_{k}r_{k}^{M}+\hat{F},$$

with $r_{k}^{M}=\langle |{\hat{u}}_{k}^{M}{|}^{2}\rangle$. Note that, in both (211) and (212), we consider all the Fourier modes. In practice, Galerkin truncation is naturally applied to these imperfect models, which greatly reduces the dimension of the imperfect system [81]. Since the goal of this subsection is to understand the role of these linear models with optimized parameters, we do not include the Galerkin truncation here. In the next subsection, we will apply the Galerkin truncation for ${\hat{u}}_{k}^{M}$ and reach a suite of low-order models in approximating both the velocity and the tracer fields.

Under the approximations in (211) and (212), the background flow $v^{M}=(U^{M}\left(t\right),v^{M}\left(x_{j},t\right)$ can be constructed as before for the mean cross-sweep $U^{M}$ and the shearing flow $v^{M}$ in the tracer model (202),

(213) $$U^{M}\left(t\right)={\bar{u}}^{M}\left(t\right)+{\hat{u}}_{0}^{M}\left(t\right),\;v^{M}\left(x_{j},t\right)=\sum_{k\neq 0}{\hat{u}}_{k}^{M}\left(t\right)e^{2\pi ikx_{j}}.$$

Now, the problem is converted to finding systematic strategies of assigning values to the three undetermined coefficients $\gamma_{u_{k}},\omega_{u_{k}},\sigma_{u_{k}}$ so that the tracer structure (intermittency) can be reconstructed from this imperfect model. They should be chosen in an unambiguous way according to the true steady state statistics of the system (which is available from observations). In comparison with the original equation for each mode described in (210), the linear Gaussian approximation of L96 system replaces the nonlinear interaction part in the second line of (210) by linear damping and rotation together with a white noise

(214) $$\sum_{m\neq 0}{\hat{u}}_{k+m}{\hat{u}}_{m}^{*}\left(e^{2\pi i\frac{2m+k}{J}}-e^{-2\pi i\frac{m+2k}{J}}\right)∼\left(-\gamma_{u_{k}}+i\omega_{u_{k}}\right){\hat{u}}_{k}^{M}+\sigma_{u_{k}}{\dot{W}}_{k}.\;$$

The white noise $\sigma_{u_{k}}{\dot{W}}_{k}$ is added to each Fourier mode in order to make sure that the system converges to the consistent equilibrium steady state spectra. $\gamma_{u_{k}}$ represents the damping that neutralizes the additional energy from the white noise. The imaginary component $\omega_{u_{k}}$ is the additional degree of freedom for tuning the autocorrelation function (or in other words, to control the `memory’ of this mode of its previous history). Note that the quasi-linear part with U(t) in the first line of the formula (210) is also included in the coefficients $\gamma_{u_{k}},\omega_{u_{k}}$. It is discovered that even under this linear flow field with Gaussian statistics, intermittency with fat-tailed distributions can be generated in the steady state tracer distributions [193,195]. Here, the challenge is whether we can still capture the correct structure in the tracer spectra and density functions, especially for the intermittency, under these imperfect linear models. Therefore, judicious choice of the model parameters needs to be investigated.

One of the simplest and most direction way to estimate the undetermined coefficients $\gamma_{u_{k}},\omega_{u_{k}},\sigma_{u_{k}}$ is through the mean stochastic model (MSM) [15,41] by calibrating the energy (variance) $E_{k}=\langle |{\hat{u}}_{k}\left(t\right)-\langle {\hat{u}}_{k}\rangle {|}^{2}|\rangle$ and decorrelation time τ in (44) of the truth (known as “MSM tuned parameters”). Note that the decorrelation time $\tau =T_{k}+i\theta_{k}$ here contains real and imaginary parts, fitting both as well as the energy provides three conditions. Despite the simplicity in this mean stochastic model, reasonably skillful prediction in uncertainty quantification as well as filtering under this strategy have been obtained for some turbulent systems [193,195]. However, MSM still suffers several shortcomings when strong nonlinearity takes place in the system. Most importantly, the decorrelation time $\tau =T_{k}+i\theta_{k}$ involves only the time-integrated effects in each mode. This works well when the system is strongly mixing within a nearly Gaussian regime, whereas, when non-Gaussian features become crucial in the system, the pointwise decaying process of the entire autocorrelation function R(t) becomes important and we need take into account the temporal performance of the autocorrelation in the linear model approximation. This has already been seen in the simple example in Section 2.5. In fact, the autocorrelation function becomes strongly oscillatory when F=5 in the L96 model, which shows the insufficiency of fitting only the decorrelation time.

Therefore, following the physics-tuned linear regression modeling strategy in Section 6.2 and the information-theoretic framework of calibrating the autocorrelation function in Section 2.5, we fit the autocorrelation function of each ${\hat{u}}_{k}$ by the spectral information criteria (47) and (48). Note that the linear Gaussian model in (211) has explicit solution for the autocorrelation function and power spectrum (52), which provides an extremely efficient way of calibrating the two parameters $\gamma_{u_{k}},\omega_{u_{k}}$. The remaining parameter $\sigma_{u_{k}}$ is calibrated by fitting the energy. Finally, we keep the tracer equation to be the same in this example. Finding a reduced order model for the tracer equation following the general strategy in Section 6.1 will be discussed in the next subsection.

**Figure 41:**
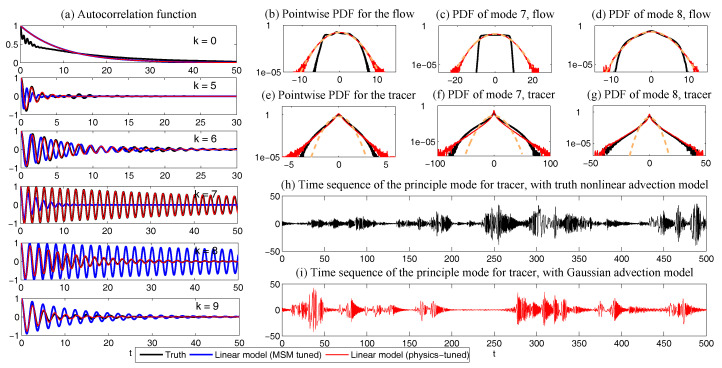
Comparison of the statistical feature of both the advection field *v* and the tracer *T*. The true advection model is given by L96 system (Figure 41) with F=5 (weakly chaotic regime). (**a**): fitting of the autocorrelation functions for representative Fourier modes k=0 and 5≤|k|≤9 of the flow state variables ${\hat{u}}_{k}$, where only the real part is shown. The autocorrelation function from the true system is plotted in thick black lines, and the results from MSM is in blue lines, while the optimal model results from tuning parameters in spectral density functions are shown in red lines. Note that black and red lines are largely overlapped together; (**b**–**g**): comparison of probability density functions in logarithm scale, where the brown dashed curves are the Gaussian fit. Note that modes 7 and 8 are the most energetic modes in L96 model with F=5; (**h**,**i**): sample trajectories of the tracer principal mode from the perfect model and linear Gaussian model with physics-tuned parameters.

In Figure 41, the statistical features of both the advection field *v* and the tracer *T* are shown. Here, the parameters in the true model (205), (209) and (210) are as follows:

(215) $$d_{T}=0.1,\;\alpha =2,\;\kappa =0.001,\;d_{u}=1,\;F=5.$$

Note that F=5 corresponds to the weakly chaotic regime in L96 model, which results in a very slow mixing and therefore the autocorrelation function in a certain modes decays quite slowly with strong oscillations. See the black curves Panel (a) of Figure 41. It is expected from Section 2.5 that using the strategy of MSM by fitting only the decorrelation time results in a large bias, which is clearly indicated by the blue curve in Panel (a). On the other hand, with the physics-tuned parameters calibrated based on fitting the autocrrelation function via the spectral density, the imperfect model provides a significantly accurate estimation of the autocorrelation function even in this tough regime. Next, the comparison of the PDFs associated with both *v* and *T* are shown in Panels (b)–(g). Clearly, the linear Gaussian models of *v* fail to capture the sub-Gaussian PDFs of the velocity field, which indicates an information barrier. Nevertheless, the nonlinear interaction between *U* and *T* allows the imperfect model to capture the non-Gaussian features in the tracer field with fat-tailed PDFs in both physical space (Panel (e)) and spectrum space (Panels (f)–(g)). The sample time series using the linear Gaussian velocity model (209) and (210) with the physics-tuned parameters also resembles that of the truth with significant intermittency (Panels (h) and (i)). On the other hand, the linear model with MSM tuned parameters (fitting only the decorrelation time) fails to capture these features (not shown here). See [81] for more discussions and numerical tests in other regimes (F=8 and F=16).

#### 6.3.2. Predicting Passive Tracer Extreme Events with Low-Order Stochastic Models

Now, we aim at answering the second question proposed at the beginning of this section. That is, how to design an unambiguous reduced-order modeling (ROM) strategy with high prediction skill of the tracer field [82]. Here, we consider a more realistic and complicated advection flow v(x,t), which is described from the solution of the two-layer quasi-geophysics (QG) equation [121,143]

(216) $$\begin{array}{cc} \frac{\partial q_{j}}{\partial t}+v_{j}\cdot \nabla q_{j}+\left(\beta +k_{d}^{2}U_{j}\right)\frac{\partial \psi_{j}}{\partial x} & =-\delta_{2j}r\Delta \psi_{j}-\nu \Delta^{s}q_{j},\\ q_{j}=\Delta \psi_{j}+\frac{k_{d}^{2}}{2}\left(\psi_{3-j}-\psi_{j}\right), & \;v_{j}=\left(U_{j},0\right)+v_{j}^{\prime }. \end{array}$$

Above, the subindex j=1,2 is used to represent the upper and lower layer of the two-layer flow model. The two-dimensional incompressible velocity field $v_{j}$ is decomposed into a zonal mean cross sweep, $\left(U_{j},0\right)$, and a fluctuating shear flow $v_{j}^{\prime }=\nabla^{⊥}\psi_{j}=\left(-\partial_{y}\psi_{j},\partial_{x}\psi_{j}\right)$. For the passive tracer field, now we assume the background mean gradient varying in both *x* and *y* directions together with a tracer fluctuation component

(217) $$T_{j}\left(x,t\right)=\alpha \cdot x+T_{j}^{\prime }\left(x,t\right),$$

where $\alpha =(\alpha_{x},\alpha_{y})$. Plugging (217) into (202), the fluctuation part of the passive tracer model yields

(218) $$\epsilon \frac{\partial T_{j}^{\prime }}{\partial t}+v_{j}^{\prime }\left(x,t\right)\cdot \nabla T_{j}^{\prime }+U_{j}\frac{\partial T_{j}^{\prime }}{\partial x}=-\left(\alpha_{x}u_{j}+\alpha_{y}v_{j}\right)\left(x,t\right)-d_{T}T_{j}^{\prime }+\kappa \Delta T_{j}^{\prime }.$$

In (218), $v_{j}^{\prime }=\left(u_{j},v_{j}\right)$ is the fluctuating advection flow field from the solution of (216) together with a zonal mean flow $U_{j}$. In addition, a scale separation in the tracer Equation (218) with order ϵ is introduced. The difference in time scale in the tracer is through a different time scale, $\tilde{t}=\epsilon^{-1}t$, in the tracer time as in various previous works [189,193,195]. As ϵ<1, the velocity field is varying at a faster time scale than the passive tracer process, while on the other hand with ϵ>1 the tracer evolves in a more rapid rate than the advection field. A long time rescaling limit with explicit analytic tracer solutions is derived in [195] and numerical simulations for varying values of ϵ among a wide range are investigated in [192] under a much simpler linear model. In general, different intermittent features will be generated from near Gaussian statistics to distributions with fat tails as the scale separation parameter value changes [189,192].

Given periodic boundary condition in both the two-layer flow and the tracer field, we formulate the flow and tracer fields with Galerkin truncation to finite number of Fourier modes. Spatial Fourier decomposition in flow potential vorticity $q_{j}$ and passive tracer disturbance $T_{j}^{\prime }$ can be written in the expansion under modes exp(ik·x) as

(219) $$q_{j}=\sum_{k}{\hat{q}}_{j,k}e^{ik\cdot x},\;T_{j}^{\prime }=\sum_{k}{\hat{T}}_{j,k}e^{ik\cdot x}.$$

Note that here we focus on the homogeneous flow on mesoscale and therefore the periodic condition is reasonable. By projecting the tracer and flow Equations (218) and (216) to each Fourier spectral mode, equations for the spectral coefficients in each wavenumber of the two-layer tracer field ${\vec{T}}_{k}={\left({\hat{T}}_{1,k},{\hat{T}}_{2,k}\right)}^{T}$, and two-layer advection flow field ${\vec{q}}_{k}={\left({\hat{q}}_{1,k},{\hat{q}}_{2,k}\right)}^{T}$, form the set of ODEs in the spectral domain as

(220) $$\begin{array}{cc} \frac{d{\vec{T}}_{k}}{dt}+\epsilon^{-1}\sum_{m+n=k}\left(A_{km}{\vec{q}}_{m}\circ {\vec{T}}_{n}+A_{kn}{\vec{q}}_{n}\circ {\vec{T}}_{m}\right) & =-\epsilon^{-1}\left(\gamma_{T,k}+i\omega_{T,k}\right){\vec{T}}_{k}+\epsilon^{-1}G_{k}{\vec{q}}_{k}, \end{array}$$

(221) $$\begin{array}{cc} \frac{d{\vec{q}}_{k}}{dt}+\sum_{m+n=k}\left(A_{km}{\vec{q}}_{m}\circ {\vec{T}}_{n}+A_{kn}{\vec{q}}_{n}\circ {\vec{T}}_{m}\right) & =-\left(\gamma_{q,k}+i\omega_{q,k}\right){\vec{q}}_{k}, \end{array}$$

where ‘∘’ is used to denote the pointwise produce, namely $a\circ b=(a_{i}b_{i})$. The potential vorticities ${\vec{q}}_{k}$ and stream function ${\vec{\psi }}_{k}$ in two layers are related by the transform matrix $H_{k}$,

(222) $${\vec{q}}_{k}=H_{k}{\vec{\psi }}_{k}=-\left(\begin{array}{cc} {\left|k\right|}^{2}+\frac{k_{d}^{2}}{2} & -\frac{k_{d}^{2}}{2}\\ -\frac{k_{d}^{2}}{2} & {\left|k\right|}^{2}+\frac{k_{d}^{2}}{2} \end{array}\right){\vec{\psi }}_{k}$$

through the relation $q_{j}=\nabla^{2}\psi_{j}+\frac{k_{d}^{2}}{2}\left(\psi_{3-j}-\psi_{j}\right)$ in (216). The other operators and terms in the nonlinear dynamics (220) and (221) are given by

(223) $$\begin{array}{cc} A_{km}=\frac{1}{2}\left(k_{x}m_{y}-k_{y}m_{x}\right)H_{m}^{-1},\;G_{k} & =-i\alpha \cdot k^{⊥}H_{k}^{-1}=\Gamma_{k}H_{k}^{-1},\;\gamma_{T,k}=d_{t}+\kappa {\left|k\right|}^{2},\\ \omega_{T,k}=k_{x}\vec{U},\;\gamma_{q,k}={\left(0,1\right)}^{T}\circ r{\left|k\right|}^{2}H_{k}^{-1} & +{\nu |k|}^{2s},\;\omega_{q,k}=k_{x}\left(\vec{U}+\left(\beta +k_{d}^{2}\vec{U}\right)H_{k}^{-1}\right). \end{array}$$

In (223), the linear dissipation $\gamma_{T,k}$ is due to the Ekman friction applied only on the bottom layer and the hyperviscosity. The dispersion $\omega_{T,k}$ is from the rotational β-effect as well as the background zonal mean flow advection from the original Equation (216) applied on the vorticity modes.

The advection terms in the tracer and flow Equations (220) and (221) involve interactions between modes of different scales along the entire spectrum in a large dimensional phase space, thus usually high computational cost is required in achieving accurate statistical results from direct numerical simulations. In general, intermittency in a tracer field is dominated by the variability in largest scales, thus we will concentrate on the large-scale modes with wavenumber |k|≤M≪N, where *M* is the number of resolved modes and *N* is the full dimensionality of the system. Usually, we could choose *M* much smaller than *N* that only covers the essential most energetic directions in the flow system. Below, we first develop the simple strategy with linear corrections to approximate the advection flow field in the leading modes, which is similar to that in Section 6.3.1. Then, the calibration and improvement of the imperfect models due to model errors from this approximation will be discussed.

As in Section 6.3.1, in order to approximate the advection flow, the simple Gaussian approximation is adopted to replace the quadratic interactions ${\left(v\cdot \nabla q\right)}_{k}$ in the flow equations by additional linear damping and random Gaussian noise. Thus, the reduced-order advection flow equations are given by

(224) $$\begin{array}{cc} \frac{d{\vec{q}}_{M,k}}{dt} & =-\left(\gamma_{q,k}+i\omega_{q,k}\right){\vec{q}}_{M,k}-D_{q,k}^{M}{\vec{q}}_{M,k}+\Sigma_{q,k}^{M}{\dot{\vec{W}}}_{q,k},\;1\leq \left|k\right|\leq M,\\ & \;v_{M}=\nabla^{⊥}{\vec{\psi }}_{M},\;{\vec{q}}_{M,k}=H_{k}{\vec{\psi }}_{M,k}, \end{array}$$

with only Gaussian statistics generated. Only the first *M* large-scale modes 1≤|k|≤M are resolved in the reduced-order model (224). In addition to the linear dissipation and dispersion operators $\left(\gamma_{q,k},\omega_{q,k}\right)$, additional damping and noise $D_{q,k}^{M},\sigma_{q,k}^{M}$ are introduced to correct model errors due to the neglected nonlinear interactions in the flow equations. On the other hand, there is no additional model calibrations of the tracer field statistics in case of over fitting of data. The idea here is to improve the reduced-order model prediction skill by optimizing the background advection flow field, thus the reduced order passive tracer equations can be modeled through a direct truncation

(225) $$\begin{array}{cc} \frac{d{\vec{T}}_{M,k}}{d\tilde{t}}+{\left({\tilde{v}}_{M}\cdot \nabla {\vec{T}}_{M}\right)}_{k} & =\Gamma_{k}{\vec{\psi }}_{M,k}-\left(\gamma_{T,k}+i\omega_{T,k}\right){\vec{T}}_{M,k},\;1\leq \left|k\right|\leq M,\\ {\tilde{v}}_{M} & =\sum_{\left|k\right|\leq M_{1}}ik^{⊥}{\vec{\psi }}_{M,k}e^{ik\cdot x},\;M_{1}\leq M, \end{array}$$

where only the first leading modes of the advection flow 1≤|k|≤M≪N are resolved in the tracer approximation model.

Again, the major difficulty in modeling the tracer dynamics is from the accurate approximation of the tracer advection $A_{km}{\vec{q}}_{m}\circ {\vec{T}}_{n}$ in (220). Exact modeling about this nonlinear interaction term requires the flow mode solution ${\vec{q}}_{M,k}$ along the entire spectrum 0<|k|≤N, while only the first *M* leading modes are available through the reduced-order model. One crude approximation idea could be to replace the nonlinear advection in the tracer field $v\left(x,t\right)\cdot \nabla \vec{T}\left(x,t\right)$ with damping and noise in a similar fashion as the flow approximation model (224). However, as discussed in previous works [50,195], the nonlinear advection in the tracer equation is crucial in the generation of many important statistical features including the intermittency. Thus, including of nonlinear effects from the flow solution is essential, at least for the large scale modes. On the left-hand side of the Equation (225), the nonlinear advection ${\tilde{v}}_{M}\cdot \nabla {\vec{T}}_{M}$ is modeled explicitly, but only the first $M_{1}\leq M$ largest scale flow modes in the model velocity solution ${\tilde{v}}_{M}$ are used to calculate the imperfect model tracer advection. This nonlinear advection is essential in generating the accurate spectra in tracer statistics, while it is also not expensive to calculate since only leading modes are involved. The idea for this approximation is through the assumption that the dominant features in tracer statistics (such as intermittency and equilibrium spectrum in large scales) are due to the leading advection flow modes with largest energy.

Now, we calibrate the imperfect low-order linear Gaussian model for advection flow system (225) using equilibrium statistics and information theory. Such calibration procedure is divided into two steps:

1. Properly reflecting the nonlinear energy mechanism from the true system.
2. Imperfect stochastic model consistency in equilibrium statistics and autocorrelation functions.

In the first step, we aim at making sure that the imperfect model calibration parameters $\left(D_{q,k}^{M},\Sigma_{q,k}^{M}\right)$ can properly reflect the true nonlinear energy mechanism from the true system. The consistent imperfect model then can be proposed by consulting the model statistical dynamics. Therefore, it is useful to investigate the statistical equations for the second order moments from the fluctuation equations of (221). The dynamics for the covariance matrix $R_{k}^{q}=\langle {\vec{q}}_{k}{\vec{q}}_{k}^{*}\rangle$ of flow vorticity can be derived as a 2×2 blocked system for each wavenumber [117],

(226) $$\frac{dR_{k}^{q}}{dt}+L_{k}\left(\bar{q}\right)R_{k}^{q}+R_{k}^{q}L_{k}^{*}\left(\bar{q}\right)+Q_{F}^{q}=\left(L_{k}^{q}+D_{k}^{q}\right)R_{k}^{q}+R_{k}^{q}{\left(L_{k}^{q}+D_{k}^{q}\right)}^{*},\;\left|k\right|\leq N.$$

The linear operators $\left(L^{q},D^{q}\right)$ represent the skew-symmetric dispersion and dissipation effects from the right-hand side of (221). The additional operator $L_{k}\left(\bar{q}\right)$ represents the interactions with a non-zero statistical mean state, where internal instability occurs with positive growth rate. Most importantly, the nonlinear interactions between different spectral modes introduce the additional nonlinear flux term $Q_{F}^{q}$ indicating higher-order interactions, that is,

(227) $$Q_{F}^{q}\left({\vec{q}}_{k}\right)=\frac{1}{2}\sum_{m+n=k}\langle \left(A_{km}{\vec{q}}_{m}\circ {\vec{q}}_{n}+A_{kn}{\vec{q}}_{n}\circ {\vec{q}}_{m}\right){\vec{q}}_{k}^{*}\rangle .$$

Therefore, the small and large scale modes are linked through third-order moments $\langle {\vec{q}}_{m}{\vec{q}}_{n}{\vec{q}}_{k}^{*}\rangle$ in (227) between the triad modes m+n=k. The nonlinear flux $Q_{F}^{q}$ plays the central role in the energy mechanism that balances the unstable directions due to internal instability from the linear operators. Here, our focus is on the low-order stochastic realization in (224) of the statistical closure model of (226), thus solving the statistical Equation (226) directly is not favorable considering its complexity.

Below, we follow the general framework developed in Section 6.1 to determine the reduced order model. The nonlinear flux $Q_{F}^{q}$ in (227) corresponds to the unresolved nonlinear effects in the stochastic model in (224). Thus, it is useful to exploit the nonlinear flux $Q_{F}^{q}$ so that the imperfect model parameters $\left(D_{q}^{M},\Sigma_{q}^{M}\right)$ in (224) can be proposed according to the true model energy transfer mechanism. Especially in statistical equilibrium, as t→∞ the nonlinear fluxes can be calculated easily from the localized lower-order moments

(228) $$Q_{F,eq}^{q}=\left(L_{k}^{q}+D_{k}^{q}-L_{k}\left({\bar{q}}_{eq}\right)\right)R_{k,eq}^{q}+R_{k,eq}^{q}{\left(L_{k}^{q}+D_{k}^{q}-L_{k}\left({\bar{q}}_{eq}\right)\right)}^{*}.$$

Next, we further decompose the matrix $Q_{F}^{q}=Q_{F}^{q,+}+Q_{F}^{q,-}$ by singular value decomposition into positive-definite and negative-definite components. The positive definite part $Q_{F}^{q,+}$ illustrates the additional energy that injected into this mode from other scales, while the negative definite part $Q_{F}^{q,-}$ shows the extraction of energy through nonlinear transfer to other scales. In adopting the true equilibrium statistics from $Q_{F,eq}^{q}$, the true model energy transfer mechanism is respected and the least artificial effect is introduced into the imperfect approximation model. Considering all these aspects, the first proposal for the linear damping and Gaussian random noise correction can be introduced as

(229) $$D_{q,k}^{eq}=-\frac{1}{2}Q_{F,eq,k}^{q,-}{\left(R_{k,eq}^{q}\right)}^{-1},\;\Sigma_{q,k}^{eq}={\left(Q_{F,eq,k}^{q,+}\right)}^{1/2}.$$

The additional damping is from the negative definite equilibrium flux $Q_{F,eq}^{q,-}$ and the positive definite equilibrium flux $Q_{F,eq}^{q,+}$ acts as additional noise to the system. The above additional damping and noise (229) offer a desirable quantification for the minimum amount of corrections to stabilize the system with consistent equilibrium statistics for the mean and variance. This is the same idea applied to the statistical modified quasi-linear Gaussian closures developed in [45].

As discussed in Section 6.1.3, the above estimation of parameters (229) may not be optimal for the reduced-order Gaussian model considering that: (i) it only guarantees marginal stability in the unstable modes for equilibrium; and more importantly (ii) the time mixing scale in each mode (represented by the autocorrelation functions) may still lack the accuracy in the approximation using only equilibrium information. The nonlinear energy transferring mechanism may change with large deviation from the equilibrium case when intermittent fluctuations are present. The shortcomings for purely using the approximation (229) only from equilibrium statistics can be observed from the various numerical simulations [82]. As a further correction, we propose additional terms on top of (229) with a simple constant damping for all the spectral modes and an additional noise accordingly to make sure consistency in energy,

(230) $$Q_{M,k}^{add}=-D_{M}^{add}R_{M,k}+{\left(\Sigma_{M,k}^{add}\right)}^{2},\;D_{M}^{add}=diag\left\{d_{M}+i\omega_{M},d_{M}-i\omega_{M}\right\}.$$

The correction term in (230) is aimed to offer stabilizing effects in the marginal stable equilibrium form (229), and to offer further corrections in modeling the autocorrelation function that is important for the mixing rate in each spectral mode. Combining (229) and (230), the additional damping and noise corrections for the reduced-order flow vorticity model (224) are given in the following form

(231) $$D_{q,k}^{M}=-\frac{1}{2}Q_{F,eq,k}^{q,-}{\left(R_{k,eq}^{q}\right)}^{-1},\;\Sigma_{q,k}^{M}={\left(Q_{F,eq,k}^{q,+}+{\left(\Sigma_{M,k}^{add}\right)}^{2}\right)}^{1/2}.$$

Comparing with the exact true system (221), the reduced-order approximation is equivalent to replacing the nonlinear interaction terms with the judiciously calibrated damping and noise in consideration with both the equilibrium energy transfer mechanism and further sensitivity correction.

Now, we move to the second step. Here, we tune the undermined model parameters $\left(D_{M}^{add},\Sigma_{M}^{add}\right)$ to guarantee the imperfect stochastic model consistency in equilibrium statistics (the leading two moments) and autocorrelation functions. The procedure here is exactly the same as that in Section 6.3.1, where information theory developed in Section 2.5 is used for calibrating the autocorrelation function. Thus, we neglect the details here.

Finally, let us show a simple example for predicting the tracer statistics using the low-order model prediction. The example here has the same setup as one of the regimes considered in [81], that is, the high latitude atmosphere regime, where the parameters are given as follows:

(232) $$\begin{array}{cc} N=128,\;\beta =1,\;F=4, & \;U=0.1,\;r=0.2,\\ \nu =10^{-13},\;s=4,\;d_{T}=0.1 & \;\kappa =10^{-3},\;\alpha =1. \end{array}$$

Here, N=128 is the number of grid points in each direction. The true statistics are calculated by a pseudo-spectra code with 128×128×2 grid points in total. The zonal mean flow $\vec{U}=\left(U,-U\right)$ is taken as the same strength with opposite directions in the two layers. In the tracer simulations, for simplicity, we consider the mean gradient along y direction, that is to assume, $T=T^{\prime }+\alpha y$. This assumption is representative in many previous investigations [117,193,195]. The scale separation parameter ϵ in this example is chosen to be $\epsilon^{-1}=5$ such that intermittency is prominent. In the reduced-order model, we only compute the modes k≤M=10 in largest scales, compared with the true system resolution N=128.

**Figure 42:**
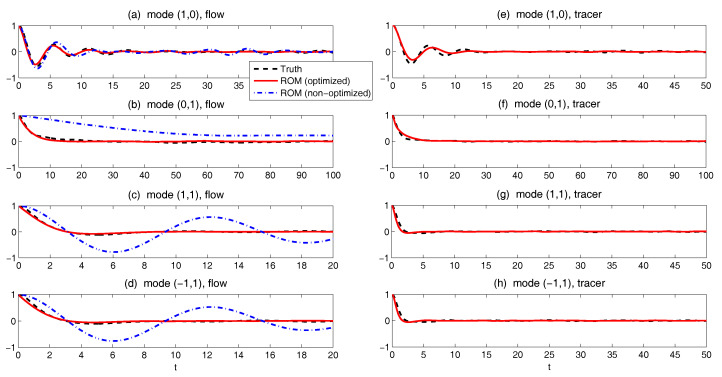
Prediction of the reduced-order autocorrelation functions in the flow (**a**–**d**) and tracer fields (**e**–**h**) in the first four most energetic modes in high latitude atmosphere regime with parameters $\left(\epsilon^{-1},d_{T}\right)=\left(5,0.1\right)$. The truth is shown in black dashed lines and the reduced-order model (ROM) prediction in red lines.

**Figure 43:**
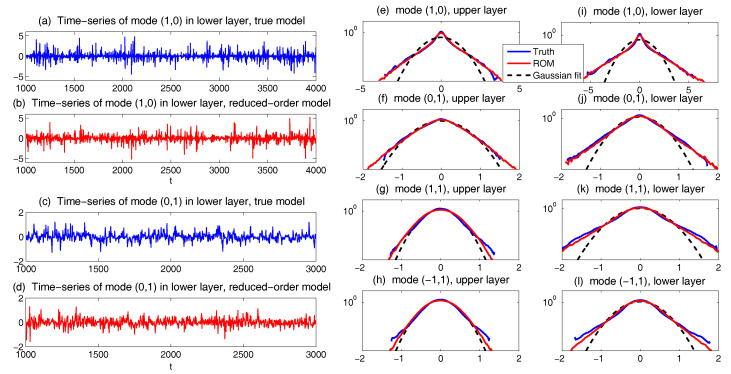
Prediction of tracer intermittency in high latitude atmosphere regime with parameters $\left(\epsilon^{-1},d_{T}\right)=\left(5,0.1\right)$. (**a**–**d**): the time-series for the first two leading modes (1,0) and (0,1) between true model and reduced-order model (ROM) results; (**e**–**l**): comparison of the PDFs in the first four modes between the truth in blue and reduced model prediction in red with the Gaussian fit in dashed black lines.

The autocorrelation functions of the first four leading modes (1,0),(0,1),(1,1) and (-1,1) in both the flow stream functions and the tracer fields are plotted in Figure 42. It is clear that for both the flow and tracer fields, the reduced order model with optimized parameters calibrated by information theory succeeds in capturing the autocorrelation function of the truth. As a comparison, equipped with the parameters with no additional corrections $d_{M}=0,\sigma_{M}=0$, the reduced order model has a huge bias in recovering the autocorrelation function of the flow field. Next, we test the prediction skill of the turbulent tracer statistics in the reduced-order model. As we have seen in Section 6.3.1 and the discussions above, the nonlinear advection in the tracer equation $v_{M}\cdot \nabla T_{M}$ is important for the final tracer statistical structure. The goal here is to see whether the intermittent and other features in the tracer field can be accurately predicted using only principal modes with largest variance in $v_{M}$ in calculating the nonlinear term. Figure 43 compares the representative time-series and tracer PDFs of the leading modes in statistical steady state. Despite only 0.6% of the modes being involved in the flow field, the fat-tails in the distribution functions of the tracer can be captured, and similar characteristic structures can be seen in the truth and reduced model time series. In fact, the high skill of recovering the non-Gaussian features is due to the fact that the advection term $v_{M}\cdot \nabla T_{M}$ is captured quite well even with such a crude truncation of the flow field. The results in Figure 42 and Figure 43 imply the skillful predictions using the reduced order model with the optimized parameters. In [82], the recovered tracer field using different truncation size *M* has been explored. It is important to note that with only the first two modes M=2 being included in calculating the nonlinear advection, larger errors appear due to the insufficient quantification for flow advection. The recovering skill of other statistical features such as the power spectrum and eddy diffusivity approximations for the tracers in this regime as well as the test examples in other regimes have also been systematically discussed in [82].

## 7. Conclusions

This research expository article discusses various important topics related to model error, information barriers, state estimation and prediction in complex multiscale systems. A recent information-theoretic framework is developed and summarized, which is applied together with other mathematical tools to study all these topics. It is also combined with novel reduced-order nonlinear modeling strategies for understanding and predicting complex multiscale systems. The contents of this article include the general mathematical framework and theory, effective numerical procedures, instructive qualitative models, and concrete models from climate, atmosphere and ocean science. The information-theoretic framework is developed in Section 2 and is applied to understand various information barriers in the presence of model error via instructive stochastic models. In Section 3, the information-theoretic framework is adopted to assess model error in state estimation and prediction with examples coming from both complex scalar models and spatially-extended multiscale turbulent systems. The advantage of the information-theoretic framework over the traditional path-wise measurements are illustrated. Section 4 deals with sensitivity and linear statistical response using the fluctuation–dissipation theorem. An efficient and effective algorithm in finding the most sensitive change directions using information theory is also included in this section. In Section 5, a novel framework of data-driven physics-constrained nonlinear stochastic models and predictions is developed and is applied to predicting the MJO which contains strong intermittent instabilities and extreme events. Section 6 includes the development of the new effective reduced-order models that involve higher order statistical features but nevertheless remain computationally efficient. These new models together with the information-optimization model calibration strategy are applied to predicting passive tracers extreme events.

The simple imperfect models used in Section 2 and Section 4 are all motivated from the strategies that are commonly used in practice for approximating extremely complicated systems such as GCMs. Therefore, the information barriers shown in this article clearly indicate the deficiency of these strategies and point out the directions of improving the imperfect models. The computationally efficient reduced-order modeling framework developed in Section 6 is promising in dealing with many complicated real-world issues. In particular, including higher order statistical features using the novel approach allows these new reduced-order models to capture the nonlinear evolution and non-Gaussian characteristics in both the dynamics and statistics. Therefore, these models are able to overcome those information barriers resulting from the linear tangential or Gaussian closure approximations as well as the ignorance of the cross-correlations between different modes or grid points. In addition to studying the spatially-extended systems associated with predicting passive tracers extreme events, the other applications of these low-order modeling strategies are good future directions. Note that these low-order modeling strategies combined with FDT can also be powerful tools to study the effective statistical control of complex turbulent dynamical systems [206]. On the other hand, although great efforts have been put in understanding the sources of model errors in data assimilation (or filtering), the representation error was nevertheless overlooked in the past. In Section 3, the issue of representation error is emphasized and some practical strategies have been proposed and tested. More systematic studies are required in this area as future works. It is also of great importance to study filtering and prediction as a whole and understand the model error and improved strategies for both procedures instead of focusing solely on the filtering part. In addition, as is briefly discussed in Section 3.5.2, combining the Euler and Lagrangian observations is another interesting topic in improving the skill of data assimilation and prediction of spatially-extended systems as well as quantifying the uncertainty reduction. Finally, it has been shown in Section 5 that the data-driven physics-constrained nonlinear stochastic modeling framework has several salient advantages over the purely data-driven non-parametric methods in terms of both understanding the underlying physics and obtaining skillful predictions. These advantages include a much shorter training phase, a systematic calibration strategy, gaining clear physical insights and reaching model robustness. Applying the data-driven physics-constrained nonlinear stochastic modeling framework to many other complex real-world problems is potentially important. Many related issues remain as future work.

## Figures and Tables

**Table 1:** Summary of the four setups in filtering the 3×3 system in (104). The four setups are: Full observations, full forecast model (F/F); partial observations, full forecast model (P/F); partial observations, reduced forecast model (P/R); and partial observations, reduced forecast model and tuned observational noise level with inflation (P/R tuned). Here, *√* means the strategy works for small, moderate and moderately large $\Delta_{obs}$. Small $\Delta_{obs}$ implies $\Delta_{obs}\leq 0.4$ which is roughly the decorrelation time of $u_{2}$ and $u_{3}$ in ϵ=0.1 regime. Moderate $\Delta_{obs}$ means $0.4\leq \Delta_{obs}\leq 1.2$ and moderately large $\Delta_{obs}$ is up to $\Delta_{obs}\leq 2$, which is nevertheless below the decorrelation time of $u_{1}$ since $u_{1}$ has a slow-varying time-periodic forcing.

	F/F	P/F	P/R	P/R Tuned
ϵ=0.1				
Filter $u_{1}$	*√*	*√*	*√*	*√*
Pred. $u_{1}$	*√*	*√*	*√*	*√*
Filter $u_{2},u_{3}$	small and moderate $\Delta_{obs}$	small and moderate $\Delta_{obs}$	N/A	N/A
Pred. $u_{2},u_{3}$	small and moderate $\Delta_{obs}$	small $\Delta_{obs}$	N/A	N/A
ϵ=1.0				
Filter $u_{1}$	*√*	small to moderate $\Delta_{obs}$	moderate $\Delta_{obs}$	small to moderate $\Delta_{obs}$
Pred. $u_{1}$	*√*	small to moderate $\Delta_{obs}$	moderate $\Delta_{obs}$	small to moderate $\Delta_{obs}$
Filter $u_{2},u_{3}$	small to moderate $\Delta_{obs}$	small $\Delta_{obs}$ for $u_{2}$	N/A	N/A
Pred. $u_{2},u_{3}$	small to moderate $\Delta_{obs}$ for $u_{2}$	small $\Delta_{obs}$ for $u_{2}$	N/A	N/A
	and small $\Delta_{obs}$ for $u_{3}$			

**Figure A1:**
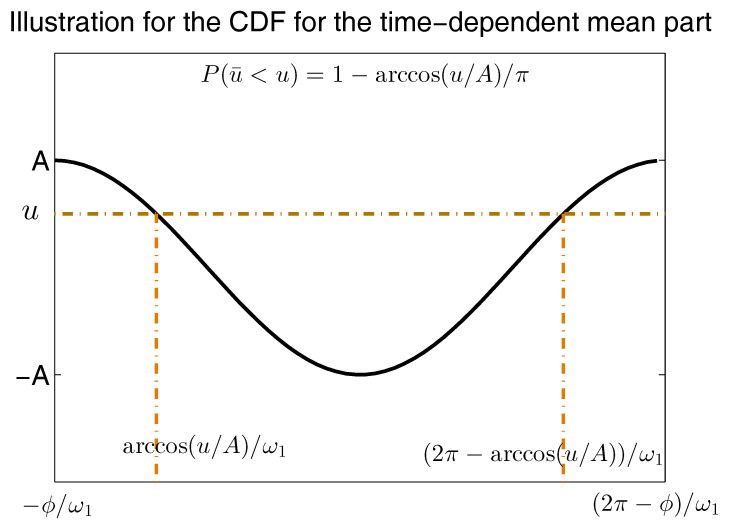
Illustration of computing the cumulative distribution function (CDF) of the deterministic mean $\bar{u}\left(t\right)$.

**Figure A2:**
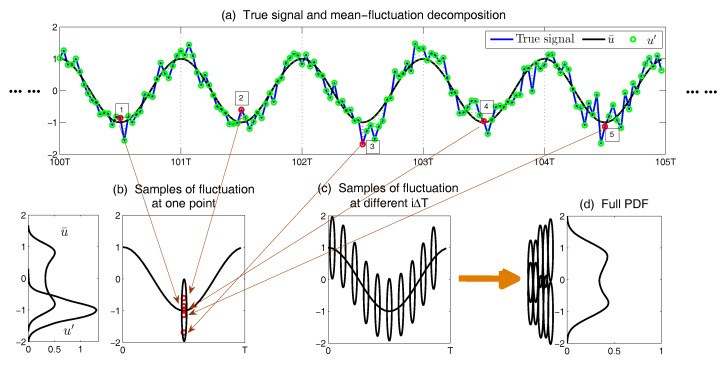
Illustration of the mean-fluctuation decomposition and the infinite Gaussian mixture PDF. (**a**): the true signal and its mean-fluctuation decomposition. Here, the signal within only five periods is shown; (**b**): the collection of the fluctuations at fixed time within each period, namely nT+iΔT with *i* fixed and n=1,…,N, which gives a Gaussian PDF in (A32). The left subpanel shows the non-Gaussian time-averaged PDF of the deterministic mean $\bar{u}\left(t\right)$ and the Gaussian PDF of $\$u^{\prime }\left(nT+i\Delta T\right)$ for a fixed *i*; (**c**): repeating (b) at different *i*; (**d**): the full PDF given by a Gaussian mixture from all the Gaussian PDFs in (c) with ΔT→0.

**Figure A3:**
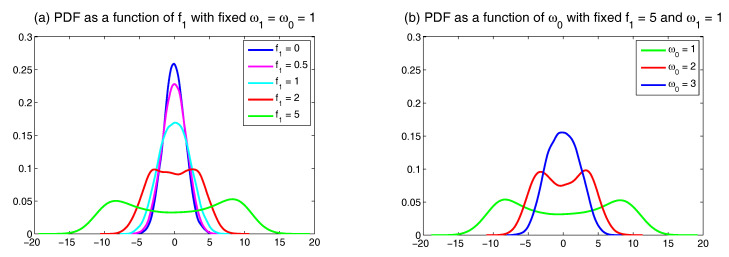
The PDF of *u* averaged over a single long trajectory. (**a**): the PDF as a function of the amplitude of time-periodic forcing $f_{1}$ in a resonant forcing setup $\omega_{1}=\omega_{0}=1$; (**b**): the PDF as a function of oscillation frequency $\omega_{0}$ with fixed $f_{1}=5$ and $\omega_{1}=1$.

## Appendix A. Derivations of Fisher Information from Relative Entropy

Here, we include the details of the derivations of Fisher information (149) and (150) from the relative entropy. Let’s consider the two PDFs $\pi_{\theta }\left(u\right)$ and $\pi_{\theta^{\prime }}\left(u\right)$ with $\theta^{\prime }=\theta +\delta \theta$ and δθ is a small increment. Applying the Talyor’s expansion to $\pi_{\theta }$ and $\ln\pi_{\theta }$ yields

(A1) $$\begin{array}{cc} \pi_{\theta^{\prime }} & =\pi_{\theta }+\nabla_{\theta }\pi_{\theta }\delta \theta +\frac{1}{2}\delta \theta^{T}\nabla_{\theta }^{2}\pi_{\theta }\delta \theta +O\left(\delta \theta^{3}\right),\\ \ln\pi_{\theta^{\prime }} & =\ln\pi_{\theta }+\frac{1}{\pi_{\theta }}\nabla_{\theta }\pi_{\theta }\delta \theta +\frac{1}{2}\delta \theta^{T}\left(-\frac{1}{\pi_{\theta }^{2}}{\left(\nabla_{\theta }\right)}^{2}+\frac{1}{\pi_{\theta }}\nabla_{\theta }^{2}\pi_{\theta }\right)\delta \theta +O\left(\delta \theta^{3}\right). \end{array}$$

With (A1) in hand, now we compute the relative entropy in (149):

(A2) $$\begin{array}{cc} P(\pi_{\theta^{\prime }},\pi_{\theta }) & =\int \pi_{\theta^{\prime }}\ln\frac{\pi_{\theta^{\prime }}}{\pi_{\theta }}\\ & =\int \pi_{\theta^{\prime }}\ln\pi_{\theta^{\prime }}-\int \pi_{\theta^{\prime }}\ln\pi_{\theta }\\ & =\int \left(\pi_{\theta }+\nabla_{\theta }\pi_{\theta }\delta \theta +\frac{1}{2}\delta \theta^{T}\nabla_{\theta }^{2}\pi_{\theta }\delta \theta \right)\left(\ln\pi_{\theta }+\frac{1}{\pi_{\theta }}\nabla_{\theta }\pi_{\theta }\delta \theta +\frac{1}{2}\delta \theta^{T}\left(-\frac{1}{\pi_{\theta }^{2}}{\left(\nabla_{\theta }\right)}^{2}+\frac{1}{\pi_{\theta }}\nabla_{\theta }^{2}\pi_{\theta }\right)\delta \theta \right)\\ & \;-\int \left(\pi_{\theta }+\nabla_{\theta }\pi_{\theta }\delta \theta +\frac{1}{2}\delta \theta^{T}\nabla_{\theta }^{2}\pi_{\theta }\delta \theta \right)\ln\pi_{\theta }+O\left(\delta \theta^{3}\right)\\ & =\int \pi_{\theta }\ln\pi_{\theta }+\nabla_{\theta }\pi_{\theta }\delta \theta +\frac{1}{2}\pi_{\theta }\delta \theta^{T}\left(-\frac{1}{\pi_{\theta }^{2}}{\left(\nabla_{\theta }\right)}^{2}+\frac{1}{\pi_{\theta }}\nabla_{\theta }^{2}\pi_{\theta }\right)\delta \theta +\nabla_{\theta }\pi_{\theta }\ln\pi_{\theta }\delta \theta +\delta \theta^{T}\frac{1}{\pi_{\theta }}{\left(\nabla_{\theta }\pi_{\theta }\right)}^{2}\delta \theta \\ & \;+\frac{1}{2}\ln\pi_{\theta }\delta \theta^{T}\nabla_{\theta }^{2}\pi_{\theta }\delta \theta -\pi_{\theta }\ln\pi_{\theta }-\nabla_{\theta }\pi_{\theta }\ln\pi_{\theta }\delta \theta -\frac{1}{2}\ln\pi_{\theta }\delta \theta^{T}\nabla_{\theta }^{2}\pi_{\theta }\delta \theta +O\left(\delta \theta^{3}\right)\\ & =\nabla_{\theta }\int \pi_{\theta }\delta \theta +\frac{1}{2}\delta \theta^{T}\left(\int \frac{1}{\pi_{\theta }}{\left(\nabla_{\theta }\pi_{\theta }\right)}^{2}\right)\delta \theta +\frac{1}{2}\delta \theta^{T}\left(\nabla_{\theta }^{2}\int \pi_{\theta }\right)\delta \theta +O\left(\delta \theta^{3}\right)\\ & =\frac{1}{2}\delta \theta^{T}\cdot \left(\int \frac{1}{\pi_{\theta }}{\left(\nabla_{\theta }\pi_{\theta }\right)}^{2}\right)\cdot \delta \theta +O\left(\delta \theta^{3}\right)\\ & =\frac{1}{2}\left(\int \frac{{\left(\delta \theta \cdot \nabla_{\theta }\pi_{\theta }\right)}^{2}}{\pi_{\theta }}\right)+O\left(\delta \theta^{3}\right), \end{array}$$

where we have made use of the fact that $\int \pi_{\theta }\equiv 1$ and therefore $\nabla_{\theta }\int \pi_{\theta }=0$. Some regularity assumptions are also required [207] such that the integral and gradient operator can be interchanged. Clearly, the final result is the Fisher information. Note that δθ here is λ in (150).

## Appendix B. Details of the Canonical Model for Low Frequency Atmospheric Variability

Here, we provide more details of the canonical model for low frequency atmospheric variability [116,170] with cubic nonlinearity and correlated additive and multiplicative (CAM) noise, which has been used in Section 4.3 and Section 6.2.

The model reads

(A3) $$\frac{dx}{dt}=\left(f+ax+bx^{2}-cx^{3}\right)+\left(A-Bx\right){\dot{W}}_{C}+\sigma {\dot{W}}_{A}.$$

The Fokker–Planck equation for the evolution of the PDF is given by

$$\frac{\partial \pi }{\partial t}=-\frac{\partial }{\partial x}\left[(f+ax+bx^{2}-cx^{3})\pi \right]+\frac{1}{2}\frac{\partial^{2}}{\partial x^{2}}\left[({\left(Bx-A\right)}^{2}+\sigma^{2})\pi \right].$$

For the case of nonzero correlated additive and multiplicative (CAM) noise, i.e., A≠0 and B≠0, we find the following equilibrium PDF:

(A4) $$\pi \left(x\right)=\frac{N_{0}}{{\left({\left(Bx-A\right)}^{2}+\sigma^{2}\right)}^{a_{1}}}e^{d\arctan\left(\frac{Bx-A}{\sigma }\right)}e^{\frac{-c_{1}x^{2}+b_{1}x}{B^{4}}},$$

where $N_{0}$ is a normalizing constant to make π(x) integrate to one, and the new parameters can be computed via

$$\begin{array}{c} a_{1}=1-\frac{-3A^{2}c+aB^{2}+2AbB+c\sigma^{2}}{B^{4}},\;b_{1}=2bB^{2}-4cAB,\;c1=cB^{2},\\ d=\frac{d_{1}}{\sigma }+d_{2}\sigma ,\;d_{1}=2\frac{A^{2}bB-A^{3}c+AaB^{2}+B^{3}f}{B^{4}},\;d_{2}=\frac{6cA-2bB}{B^{4}}. \end{array}$$

On the other hand, in a special case of additive noise only, i.e., A=B=0, we find the following invariant PDF

(A5) $$\pi \left(x\right)=N_{0}\exp\left(\frac{2}{\sigma^{2}}\left(fx+\frac{a}{2}x^{2}+\frac{b}{3}x^{3}-\frac{c}{4}x^{4}\right)\right).$$

## Appendix C. Augmented System for Prediction and Filtering Distributions

Here, we show the details of using augmented systems for studying the prediction/filtering state estimates compared to the truth as discussed in Section 3.2.

### Appendix C.1. Augmented System for Prediction

In light of the truth (67) and the prediction mean (69), the coupled evolution of the augmented state $X_{m}:={\left(u_{m},{\bar{u}}_{m+1|m}\right)}^{T}$ is given by

(A6) $$\left(\begin{array}{c} u_{m+1}\\ {\bar{u}}_{m+1|m} \end{array}\right)=\left(\begin{array}{cc} F & 0\\ K_{m}^{M}gF^{M} & \left(1-K_{m}^{M}g\right)F^{M} \end{array}\right)\left(\begin{array}{c} u_{m}\\ {\bar{u}}_{m|m-1} \end{array}\right)+\left(\begin{array}{c} \sigma_{m+1}\\ K_{m}^{M}F^{M}\sigma_{m}^{o} \end{array}\right)+\left(\begin{array}{c} F_{m+1}\\ F_{m+1}^{M} \end{array}\right).$$

The system in (A6) is a Gaussian system and therefore its behavior is completely characterized by its mean and variance. The evolution of the mean of (A6) is given by

(A7) $$E\left(X_{m}\right)=\left(\begin{array}{cc} F & 0\\ K_{m}^{M}gF^{M} & \left(1-K_{m}^{M}g\right)F^{M} \end{array}\right)E\left(X_{m-1}\right)+\left(\begin{array}{c} F_{m+1}\\ F_{m+1}^{M} \end{array}\right).$$

With the equilibrium mean of the perfect and imperfect model ${\bar{u}}_{eq}=\frac{F_{\infty }}{1-F}$ and ${\bar{u}}_{eq}^{M}=\frac{F_{\infty }^{M}}{1-F^{M}}$, the asymptotic mean of $X_{m}$ is given by

(A8) $$E\left(X_{\infty }\right)=\lim_{m\to \infty }E\left(X_{m}\right)=\left(\begin{array}{c} {\bar{u}}_{eq}\\ \frac{1}{\left(1\;-\;F^{M}\right)+F^{M}K_{\infty }^{M}g}\left(K_{\infty }^{M}gF^{M}{\bar{u}}_{eq}+\left(1\;-\;F^{M}\right){\bar{u}}_{eq}^{M}\right) \end{array}\right),$$

where the asymptotic Kalman gain $K_{\infty }^{M}$ is given by

$$K_{\infty }^{M}=\frac{1}{2g}\left(1-\frac{g^{2}r^{M}}{|F^{M}{|}^{2}r^{o}}-\frac{1}{|F^{M}{|}^{2}}+{\left[{\left(1-\frac{g^{2}r^{M}}{|F^{M}{|}^{2}r^{o}}-\frac{1}{|F^{M}{|}^{2}}\right)}^{2}+\frac{4g^{2}r^{M}}{|F^{M}{|}^{2}r^{o}}\right]}^{1/2}\right).$$

Clearly, the asymptotic mean of the prediction state is a linear combination of the equilibrium mean of original true model ${\bar{u}}_{eq}$ and that of the forecast model of the mean ${\bar{u}}_{eq}^{M}$. With (A8), the mean bias yields

(A9) $$\lim_{m\to \infty }E\left(u_{m+1}-{\bar{u}}_{m+1|m}\right)=\frac{1-F^{M}}{\left(1-F^{M}\right)+F^{M}K_{\infty }^{M}K_{\infty }^{M}g}\left({\bar{u}}_{eq}-{\bar{u}}_{eq}^{M}\right).$$

According to (A9), the asymptotic prediction mean is equal to the equilibrium mean of the perfect model if and only if the imperfect model has the same equilibrium mean as the perfect model, namely ${\bar{u}}_{eq}={\bar{u}}_{eq}^{M}$.

Next, we derive the covariance of the augmented system. Denote the operators $F^{P}$ and $R^{P}$ as

(A10) $$F^{P}=\left(\begin{array}{cc} F & 0\\ K_{m}^{M}gF^{M} & \left(1-K_{m}^{M}g\right)F^{M} \end{array}\right),\;R^{P}=\left(\begin{array}{cc} r & 0\\ 0 & {\left(K_{m}^{M}\right)}^{2}{\left|F^{M}\right|}^{2}r^{o} \end{array}\right).$$

Denote the covariance of $X_{m}$ by $C_{m}^{P}=Cov\left(X_{m},X_{m}\right)$, where

(A11) $$C_{m}^{P}=\left(\begin{array}{cc} Cov(u_{m},u_{m}) & Cov(u_{m},{\bar{u}}_{m|m-1})\\ Cov({\bar{u}}_{m|m-1},u_{m}) & Cov({\bar{u}}_{m|m-1},{\bar{u}}_{m|m-1}) \end{array}\right)\equiv \left(\begin{array}{cc} C_{(11)m}^{P} & C_{(12)m}^{P}\\ C_{(21)m}^{P} & C_{(22)m}^{P} \end{array}\right).$$

The evolution of the covariance matrix of the augmented system is given by

(A12) $$C_{m+1}^{P}=F_{m}^{P}C_{m}^{P}F_{m}^{P*}+R_{m}^{P},$$

and the components of the asymptotic limit $C_{\infty }^{P}$ are

(A13) $$\begin{array}{cc} C_{(11)\infty }^{P} & =\frac{r}{1-{\left|F\right|}^{2}},\;C_{(12)\infty }^{P}=\frac{FF^{M*}K_{\infty }^{M}gC_{(11)\infty }^{P}}{1-FF^{M*}\left(1-K_{\infty }^{M}g\right)},\;C_{(21)\infty }^{P}=C_{(12)\infty }^{P},\\ C_{(22)\infty }^{P} & =\frac{|F^{M}{|}^{2}}{1-|F^{M}{|}^{2}{\left(1-K_{\infty }^{M}g\right)}^{2}}\left(\left(g^{2}\left(K_{\infty }^{M}\right)C_{(11)\infty }^{P}+2K_{\infty }^{M}g\left(1-K_{\infty }^{M}g\right)Re\left(C_{(12)\infty }^{P}\right)\right)+{\left(K_{\infty }^{M}\right)}^{2}r^{o}\right). \end{array}$$

With direct calculation, the dynamics of the prediction variance $r_{m+1|m}$ is

(A14) $$r_{m+1|m}=|F^{M}{|}^{2}r_{m|m}+r^{M}={\left|F^{M}\right|}^{2}\left(1-K_{m}^{M}g\right)r_{m|m-1}+r^{M}.$$

Asymptotically, it is given by

(A15) $$r_{\infty }^{P}=|F^{M}{|}^{2}\left(1-K_{\infty }^{M}g\right)r_{\infty }^{P}+r^{M}={\left|F^{M}\right|}^{2}\frac{r^{o}r_{\infty }^{P}}{g^{2}r_{\infty }^{P}+r^{o}}+r^{M}=\frac{|F^{M}{|}^{2}r^{o}}{g}K_{\infty }^{M}+r^{M}.$$

### Appendix C.2. Augmented System for Filtering

The coupled evolution of the augmented state $Y_{m}:={\left(u_{m},{\bar{u}}_{m|m}\right)}^{T}$ is

(A16) $$\begin{array}{cc} \left(\begin{array}{c} u_{m+1}\\ {\bar{u}}_{m+1|m+1} \end{array}\right)= & \left(\begin{array}{cc} F & 0\\ K_{m+1}^{M}gF & \left(1-K_{m+1}^{M}g\right)F^{M} \end{array}\right)\left(\begin{array}{c} u_{m}\\ {\bar{u}}_{m|m} \end{array}\right)+\left(\begin{array}{c} \sigma_{m+1}\\ K_{m+1}^{M}\left(g\sigma_{m+1}+\sigma_{m+1}^{o}\right) \end{array}\right)\\ & \;\;+\left(\begin{array}{c} F_{m+1}\\ \left(1-K_{m+1}^{M}g\right)F_{m+1}^{M}+K_{m+1}^{M}gF_{m+1} \end{array}\right). \end{array}$$

Again, the Gaussian statistics of the augmented state $Y_{m}$ is fully characterized by its mean and covariance. The evolution of its mean is given by

(A17) $$E\left(Y_{m}\right)=\left(\begin{array}{cc} F & 0\\ K_{m+1}^{M}gF & \left(1-K_{m+1}^{M}g\right)F^{M} \end{array}\right)E\left(Y_{m-1}\right)+\left(\begin{array}{c} F_{m+1}\\ \left(1-K_{m+1}^{M}g\right)F_{m+1}^{M}+K_{m+1}^{M}gF_{m+1} \end{array}\right).$$

When m→∞, the asymptotic mean of $Y_{m}$ is

(A18) $$E\left(Y_{\infty }\right)=\lim_{m\to \infty }E\left(Y_{m}\right)=\left(\begin{array}{c} {\bar{u}}_{eq}\\ \frac{1}{\left(1-F^{M}\right)+F^{M}K_{\infty }^{M}g}\left(K_{\infty }^{M}g{\bar{u}}_{eq}+\left(1-F^{M}\right)\left(1-K_{\infty }^{M}g\right){\bar{u}}_{eq}^{M}\right) \end{array}\right),$$

and the deviation of analysis mean from the truth signal is therefore given as follows:

(A19) $$\lim_{m\to \infty }E\left(u_{m+1}-{\bar{u}}_{m+1|m+1}\right)=\frac{\left(1-F^{M}\right)\left(1-K_{\infty }^{M}g\right)}{\left(1-F^{M}\right)+F^{M}K_{\infty }^{M}g}\left({\bar{u}}_{eq}-{\bar{u}}_{eq}^{M}\right).$$

Next, the covariance of prediction mean is

$$C_{m+1}^{A}=F_{m}^{A}C_{m}^{A}F_{m}^{A*}+R_{m}^{A},$$

where the operator $F^{A}$ and $R^{A}$ are given respectively by

$$F_{m}^{A}=\left(\begin{array}{cc} F & 0\\ K_{m+1}^{M}gF & \left(1-K_{m+1}^{M}g\right)F^{M} \end{array}\right),\;R_{m}^{A}=\left(\begin{array}{cc} r & rgK_{m+1}^{M}\\ rgK_{m+1}^{M} & {\left(K_{m+1}^{M}\right)}^{2}\left(r^{o}+g^{2}r\right) \end{array}\right).$$

The elements of covariance matrix are

(A20) $$C_{m}^{A}=\left(\begin{array}{cc} Cov(u_{m},u_{m}) & Cov(u_{m},{\bar{u}}_{m|m-1})\\ Cov({\bar{u}}_{m|m-1},u_{m}) & Cov({\bar{u}}_{m|m-1},{\bar{u}}_{m|m-1}) \end{array}\right)\equiv \left(\begin{array}{cc} C_{(11)m}^{A} & C_{(12)m}^{A}\\ C_{(21)m}^{A} & C_{(22)m}^{A} \end{array}\right),$$

with the corresponding components of the asymptotic covariance $C_{\infty }^{A}$,

(A21) $$\begin{array}{c} C_{(11)\infty }^{A}=\frac{r}{1-{\left|F\right|}^{2}},\;C_{(12)\infty }^{A}=\frac{K_{\infty }^{M}{g(|F|}^{2}C_{(11)\infty }^{A}+r)}{1-FF^{M*}\left(1-K_{\infty }^{M}g\right)},\;C_{(21)\infty }^{A}=C_{(12)\infty }^{A*},\\ C_{(22)\infty }^{A}=\frac{g^{2}{\left(K_{\infty }^{M}\right)}^{2}{\left|F\right|}^{2}C_{(11)\infty }^{A}+2K_{\infty }^{M}g\left(1-K_{\infty }^{M}g\right)Re\left(FF^{M}C_{(12)\infty }^{A}\right)+{\left(K_{\infty }^{M}\right)}^{2}\left(r^{o}+g^{2}r\right)}{1-|F^{M}{|}^{2}{\left(1-K_{\infty }^{M}g\right)}^{2}}. \end{array}$$

Thus, direct calculations result in the dynamics of prediction variance $r_{m+1|m+1}$,

(A22) $$r_{m+1|m+1}=\left(1-K_{m+1}^{M}g\right)\left(\right|F^{M}{|}^{2}r_{m|m}+r^{M}).$$

As discussed in [15], the asymptotic analysis variance is

(A23) $$r_{\infty }^{A}=\frac{r^{o}}{g}K_{\infty }^{M}.$$

Finally, we compare the asymptotic prediction and filtering variance. We have the following conclusion:

(A24) $$r_{\infty }^{A}-r_{\infty }^{P}=\frac{r^{o}}{g}K_{\infty }^{M}-\frac{|F^{M}{|}^{2}r^{o}}{g}K_{\infty }^{M}-r^{M}=\left(1-\right|F^{M}{|}^{2})r_{\infty }^{P}-r^{M}<0.$$

The details are as follows. From [15], $r_{\infty }^{P}$ satisfies the equation

(A25) $${\left(r_{\infty }^{P}\right)}^{2}+\left(\frac{r^{M}}{|F^{M}{|}^{2}}+\frac{r^{o}}{g^{2}{\left|F^{M}\right|}^{2}}-\frac{r^{o}}{g^{2}}\right)r_{\infty }^{P}-\frac{r^{o}r^{M}}{g^{2}{\left|F^{M}\right|}^{2}}=0,$$

which has a positive and negative solution. For the equilibrium imperfect model variance, we have

(A26) $$\begin{array}{cc} {\left(\frac{r^{M}}{1-|F^{M}{|}^{2}}\right)}^{2}+\left(\frac{r^{M}}{|F^{M}{|}^{2}}+\frac{r^{o}}{g^{2}{\left|F^{M}\right|}^{2}}-\frac{r^{o}}{g^{2}}\right) & \frac{r^{M}}{1-|F^{M}{|}^{2}}-\frac{r^{o}r^{M}}{g^{2}{\left|F^{M}\right|}^{2}}\\ & =\frac{{\left(r^{M}\right)}^{2}}{1-|F^{M}{|}^{2}}\left(\frac{1}{1-|F^{M}{|}^{2}}+\frac{1}{|F^{M}{|}^{2}}\right)>0. \end{array}$$

Hence, the equilibrium imperfect model variance is larger than the asymptotic filtering variance. We have

(A27) $$r_{\infty }^{A}-r_{\infty }^{P}=\left(1-\right|F^{M}{|}^{2})r_{\infty }^{P}-r^{M}<0,$$

which indicates the filtering estimate has smaller uncertainty (variance) than the prediction.

## Appendix D. Possible Non-Gaussian PDFs of a Linear Model with Time-Periodic Forcing Based on the Sample Points in a Single Trajectory

Recall the complex scalar forced OU process in (97),

(A28) $$\frac{du}{dt}=\left(-\gamma +i\omega_{0}\right)u+f_{0}+f_{1}e^{i\omega_{1}t}+\sigma \dot{W},$$

where the evolution of the statistics is given by (99). When *t* is sufficiently large, the effect of initial value decays to zero. Thus, at any time instant *t* at the attractor, the PDF u(t) is Gaussian. However, the PDF computed by taking the time average for a long trajectory at the attractor based on may not be Gaussian if the time-periodic forcing $f_{1}\neq 0$. This can be easily seen in the examples in Figure 15. In other words, the system is not ergodic [108] when the time-periodic forcing is strong. Below, we provide a mathematical quantification of such behavior. We focus on the system at the attractor and ignore the contribution from the initial value. For simplicity, we also assume $f_{0}=0$ since this constant forcing only shifts the mean of the solution by a constant and won’t affect the non-Gaussian behavior in the time-averaged PDF. Therefore, the solution of (A28), according to (98), reduces to

(A29) $$u\left(t\right)=\frac{f_{1}e^{i\omega_{1}t}}{\gamma +i(-\omega_{0}+\omega_{1})}+\sigma \int_{t_{0}}^{t}e^{\left(-\gamma +i\omega_{0}\right)\left(t-s\right)}dW\left(s\right).$$

Clearly, the true signal u(t) can be decomposed into two parts: the deterministic time-periodic mean state $\bar{u}\left(t\right)$ and the fluctuation around the time-periodic mean state $u^{\prime }\left(t\right)$:

(A30) $$\begin{array}{cc} \bar{u}\left(t\right) & =\frac{f_{1}e^{i\omega_{1}t}}{\gamma +i(-\omega_{0}+\omega_{1})},\\ u^{\prime }\left(t\right) & =\sigma \int_{t_{0}}^{t}e^{\left(-\gamma +i\omega_{0}\right)\left(t-s\right)}dW\left(s\right). \end{array}$$

For the simplicity of illustration, in the following, we only consider the real part of the true signal. Thus, $\bar{u}\left(t\right)$ and $u^{\prime }\left(t\right)$ become

(A31) $$\begin{array}{cc} \bar{u}\left(t\right) & =A\cos\left(\omega_{1}t+\phi \right),\;\phi =\arg\frac{f_{1}}{\gamma +i(-\omega_{0}+\omega_{1})},\;A=\left|\frac{f_{1}}{\gamma +i(-\omega_{0}+\omega_{1})}\right|,\\ u^{\prime }\left(t\right) & =\frac{\sigma }{\sqrt{2}}\int_{t_{0}}^{t}e^{\left(-\gamma +i\omega_{0}\right)\left(t-s\right)}dW\left(s\right). \end{array}$$

As a simple illustration, we show in Panel (a) of Figure A1 the signal u(t) and its decomposition $\bar{u}\left(t\right)$ and $u^{\prime }\left(t\right)$ within one period.

With such a mean-fluctuation decomposition, the PDF based on the samples of the long trajectory can be computed in the following way. Say the total length of the trajectory is NT, where $T=2\pi /\omega_{1}$ is the period. Now, we use take $n_{o}$ grid points with uniform increment within one period, namely $nT,nT+\Delta T,nT+2\Delta T,\ldots ,nT+(n_{o}-1)\Delta T$, where $\Delta T=T/n_{o}$ and n=1,…,N. For different *n* and fixed *i*, the time-dependent mean $\bar{u}\left(nT+i\Delta T\right)$ is the same while $u^{\prime }\left(nT+i\Delta T\right)$ is different due to the randomness in the fluctuation. It is known from (A31) that the collection of the *n* points $u^{\prime }\left(nT+i\Delta T\right)$ with n=1,…,N and fixed *i* satisfies a Gaussian distribution $N(\mu_{i},R_{i})$ with

(A32) $$\begin{array}{c} \mu_{i}=\bar{u}\left(nT+i\Delta T\right),\;\mathrm{and}\;R_{i}=\langle u^{\prime }\left(nT+i\Delta T\right)u^{\prime *}\left(nT+i\Delta T\right)\rangle =\frac{\sigma^{2}}{4\gamma }, \end{array}$$

where the variance is computed from the second equation of (A31) and it has no dependence on *t*. Therefore, the PDF of u(t) is given by the summation of all the Gaussian distributions in (A32) for $i=1,\ldots ,n_{o}$ with $n_{o}\to \infty$. See Figure A2 for an illustration. Mathematically, this can be written as a convolution,

(A33) $$p\left(u\right)=\frac{1}{C}p_{\bar{u}}*p_{u^{\prime }}=\frac{1}{C}\int_{-\infty }^{\infty }p_{\bar{u}}\left(v\right)p_{u^{\prime }}\left(u-v\right)dv,$$

with *C* a normalized constant to guarantee $\int_{-\infty }^{\infty }p\left(u\right)du=1$. Here, $p_{u^{\prime }}\left(u-t\right)$ is the Gaussian PDF with mean and variance given by (A32), namely,

(A34) $$p_{u^{\prime }}\left(u-v\right)=\frac{1}{\sqrt{\pi \sigma^{2}/\left(2\gamma \right)}}\exp\left(-\frac{{\left(u-v\right)}^{2}}{\sigma^{2}/\left(2\gamma \right)}\right).$$

To compute $p_{\bar{u}}\left(u\right)$, we refer to Figure A1. Since $\bar{u}$ is bounded by -A and *A*, the support of $p_{\bar{u}}\left(u\right)$ is also within [-A,A]. Direct calculation of $p_{\bar{u}}\left(u\right)$ is difficult. Nevertheless, the cumulative distribution function (CDF) can be used, which facilitates the derivation of $p_{\bar{u}}\left(u\right)$. Now, consider the interval $\left[-\phi /\omega_{1},\left(2\pi -\phi \right)/\omega_{1}\right]$. In fact, for any u∈[-A,A], the CDF is given by

(A35) $$P\left(\bar{u}<u\right)=\left\{\begin{array}{c} 1-\frac{2}{\omega_{1}}\arccos\frac{u}{A}/\left(\frac{2\pi }{\omega_{1}}\right)=1-\frac{1}{\pi }\arccos\frac{u}{A},\;\left|u\right|\leq A,\\ 0,\;\;\;\;\;\;\;\;\;\;\;\;|u|>A. \end{array}\right.$$

The CDF in (A35) is easily derived by making use of the fact that the samples are uniformly distributed in $t\in [-\phi /\omega_{1},\left(2\pi -\phi \right)/\omega_{1}]$, which is of length $2\pi /\omega_{1}$. With the CDF (A35) in hand, the PDF $p_{\bar{u}}\left(t\right)$ is given by the derivative of the CDF. Therefore,

(A36) $$p_{\bar{u}}=\left\{\begin{array}{c} \frac{1}{A\pi }\frac{1}{\sqrt{1-{\left(u/A\right)}^{2}}},\;\left|u\right|\leq A,\\ 0,\;\;\;\;\;\;\;\;\;|u|>A. \end{array}\right.$$

Therefore, combining (A34) and (A36) leads to the calculation of (A33).

It is clear that the full PDF depends on the variation of the time-dependent mean $\bar{u}$, and the variance of the Gaussian PDF $p_{u^{\prime }}$ for the fluctuation part. If the variation of $\bar{u}$ is much smaller than the variance of $u^{\prime }$, then the full PDF is nearly Gaussian. With the increase of the variation of $\bar{u}$ and a fixed variance of $u^{\prime }$, then the full PDF becomes bimodal. Thus, the ratio of the bandwidths associated with $p_{\bar{u}}$ and $p_{u^{\prime }}$ can be used to quantify the Gaussianity of the full PDF. The bandwidth of $p_{\bar{u}}$ is

(A37) $$L_{\bar{u}}=2A=2\left|\frac{f_{1}}{\gamma +i(-\omega_{1}+\omega_{0})}\right|.$$

On the other hand, although there is no finite support of the Gaussian distribution $p_{u^{\prime }}$, the three-sigma rule of thumb [208] is always used as an empirical bandwidth to quantify the “range” of the Gaussian distribution, where the three sigma here means the three standard deviation from the mean of the Gaussian distribution that covers 99.73% of the values of $p_{u^{\prime }}$. Thus, the bandwidth of $p_{u^{\prime }}$ is

(A38) $$L_{u^{\prime }}=2\left(3\sqrt{\frac{\sigma^{2}}{4\gamma }}\right).$$

The ratio of (A37) and (A38) is given by

(A39) $$r:=\frac{L_{\bar{u}}}{L_{u^{\prime }}}=\frac{\left|\frac{f_{1}}{\gamma +i(-\omega_{1}+\omega_{0})}\right|}{3\sqrt{\frac{\sigma^{2}}{4\gamma }}}.$$

Figure A3 shows the full PDFs with different ratio *r*. In Panel (a), the time-periodic forcing $f_{1}$ increases from $f_{1}=0$ to $f_{1}=5$. When $f_{1}=0$, the PDF is Gaussian and the system is ergodic. When $f_{1}$ increases, $L_{\bar{u}}$ becomes larger while $L_{u^{\prime }}$ is fixed. Note that, in this example, $\omega_{1}=\omega_{0}$, which means the forcing is resonant and therefore the bandwidth $L_{\bar{u}}$ is sensitive to the change of forcing amplitude $f_{1}$. With $f_{1}>1$, the bimodality in the PDF becomes significant. In Panel (b), we fix $f_{1}=5,\omega_{1}=1$ and let $\omega_{0}$ change. With the resonant forcing $\omega_{0}=\omega_{1}=1$, the PDF is significantly bimodal but with a non-resonant forcing $\omega_{0}=3$, the PDF is nearly Gaussian.
